# Kinetic Treatments for Catalyst Activation and Deactivation Processes based on Variable Time Normalization Analysis

**DOI:** 10.1002/anie.201903878

**Published:** 2019-06-07

**Authors:** Alicia Martínez‐Carrión, Michael G. Howlett, Carla Alamillo‐Ferrer, Adam D. Clayton, Richard A. Bourne, Anna Codina, Anton Vidal‐Ferran, Ralph W. Adams, Jordi Burés

**Affiliations:** ^1^ The University of Manchester School of Chemistry Oxford Road Manchester M13 9PL UK; ^2^ Institute of Chemical Research of Catalonia (ICIQ) and The Barcelona Institute of Science and Technology (BIST) Av. Països Catalans 16 43007 Tarragona Spain; ^3^ University of Leeds Institute of Process Research and Development School of Chemistry & School of Chemical and Process Engineering Leeds LS2 9JT UK; ^4^ Bruker (UK) Ltd. Banner Lane Coventry CV4 9GH UK; ^5^ ICREA Passeig Lluïs Companys, 23 08010 Barcelona Spain

**Keywords:** catalyst activation, catalyst deactivation, concentration reaction profiles, kinetics, variable time normalization analysis

## Abstract

Progress reaction profiles are affected by both catalyst activation and deactivation processes occurring alongside the main reaction. These processes complicate the kinetic analysis of reactions, often directing researchers toward incorrect conclusions. We report the application of two kinetic treatments, based on variable time normalization analysis, to reactions involving catalyst activation and deactivation processes. The first kinetic treatment allows the removal of induction periods or the effect of rate perturbations associated with catalyst deactivation from kinetic profiles when the quantity of active catalyst can be measured. The second treatment allows the estimation of the activation or deactivation profile of the catalyst when the order of the reactants for the main reaction is known. Both treatments facilitate kinetic analysis of reactions suffering catalyst activation or deactivation processes.

Processes of catalyst activation and deactivation occur at the same time as the main reaction.^1^ As a consequence, the concentration of active catalyst^2^ varies throughout the course of the reaction, affecting the reaction's intrinsic kinetic profile. This perturbation of the kinetic profile adds a layer of complexity to its analysis. Often, this complication limits the quantitative kinetic analysis to those sections of the reaction with no significant variation of catalyst concentration. Herein, we describe two treatments that facilitate a quantitative analysis of reactions involving catalyst activation or deactivation processes. One or other of these treatments can be used, depending on the available information. We show the feasibility of both methods in the kinetic analysis of real reactions with severe changes of active catalyst concentration during the course of the reaction: a hydroformylation reaction catalyzed by a supramolecular rhodium complex^3^ and an aminocatalytic Michael reaction.^4^

The two kinetic treatments presented herein are based on the recently described variable time normalization analysis (VTNA).^5^ This analysis allows the removal of the kinetic effect of any component of a reaction from the temporal concentration profiles. Therefore, if both the concentration of active catalyst and the progress of the reaction can be measured simultaneously by any appropriate method, then the intrinsic profile of the main reaction can be obtained (Figure 1 a). The resulting reaction profile of this first kinetic treatment is much simpler to analyze than the original profile and facilitates the extraction of mechanistic information, such as the orders of reaction or intrinsic turnover frequency (TOF) of the catalyst. The second kinetic treatment presented herein uses the reaction progress profile and the orders of reaction to extract the catalyst activation or deactivation profile (Figure 1 b). This profile is informative of the pathways of catalyst activation and deactivation, and their kinetics. This knowledge can help to rationally modify reaction conditions to maximize the turnover number (TON) of the reaction.

**Figure 1:**
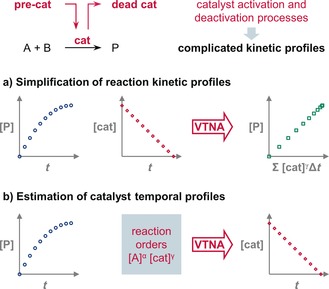
Variable time normalization analysis (VTNA) is a useful tool for the kinetic analysis of reactions with catalyst activation or deactivation processes.

The first data treatment allows the intrinsic reaction profile of a reaction altered by catalyst activation or deactivation processes to be uncovered. To demonstrate its potential, we chose two reactions with large changes in catalyst concentration. The first is an asymmetric hydroformylation catalyzed by a supramolecular rhodium complex, which requires three different units to come together to form an active catalyst: rhodium as active center, an enantiopure bisphosphite as ligand, and a rubidium salt to regulate the geometry of the catalyst (Figure 2).^3^ As the catalyst formation process is not immediate, the active catalyst concentration increases over the course of the reaction and the overall reaction profile shows a clear induction period in the formation of the product (Figure 2 a).

**Figure 2:**
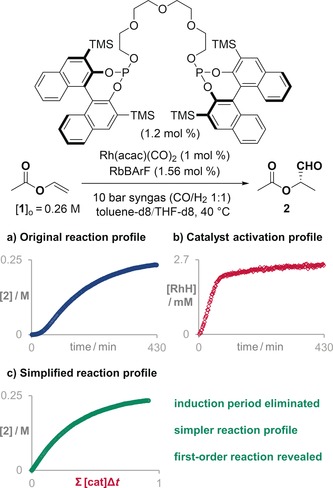
a) The original reaction profile of a hydroformylation reaction catalyzed by a supramolecular rhodium complex is corrected by b) the profile of the active catalyst to reveal c) a simple first‐order reaction profile. Key: acetylacetonate (acac), tetrakis[3,5‐bis(trifluoromethyl)phenyl]borate (BArF), tetrahydrofuran (THF), trimethylsilyl (TMS).

As the hydroformylation reaction is performed in a pressurized vessel with a constant supply of syngas, continuous monitoring of the evolution of the reaction components was challenging.^6^ To overcome this challenge, we used a Bruker InsightMR flow tube.^7^ This device continuously recirculates a small volume of the liquid reaction mixture through the reaction vessel and a modified NMR tube, enabling the on‐line monitoring of the reaction by NMR spectroscopy under these challenging reaction conditions. In this case, we were able to simultaneously monitor both the concentration of product and the amount of rhodium hydride of the assembled supramolecular complex, which is the resting state of the catalyst (**[RhH]** in Figure 2 b).^8^ The catalyst profile was used to normalize the time scale of the original progress reaction profile using VTNA.^8^ The resulting reaction profile (Figure 2 c) is much simpler than the original profile with no trace of any induction period, revealing the real first‐order profile of the original reaction. These observations imply that, under the chosen reaction conditions, the olefin–hydride insertion is the rate‐determining step.^3^, ^9^

The second reaction we investigated is the enantioselective aminocatalytic Michael addition of aldehyde to *trans*‐β‐nitrostyrene (Figure 3). This reaction is generally performed with 10–20 mol % catalyst loading^4^ because of the deactivation processes that the catalyst can suffer. Indeed, when the reaction was run at high substrate concentration and as little as 0.5 mol % catalyst, most of the catalyst was deactivated before the reaction reached completion (Figure 3 a). As a consequence, the reaction profile is curved with an apparent overall order close to one.^8^ The overlap of signals of several deactivated catalytic species in the NMR spectra made the quantification of active catalyst impossible during the last part of the reaction (Figure 3 b). Nevertheless, when the measured amount of active catalyst was used to normalize the time scale of the original reaction, the kinetic profile became an almost perfect straight line (Figure 3 c), indicative of an overall zero‐order reaction. This overall reaction order is in agreement with previous mechanistic studies performed at higher catalyst loadings.^10^ Moreover, the slope of the resulting straight line is approximately the TOF of the reaction—in this case 1.86 min^−1^.

**Figure 3:**
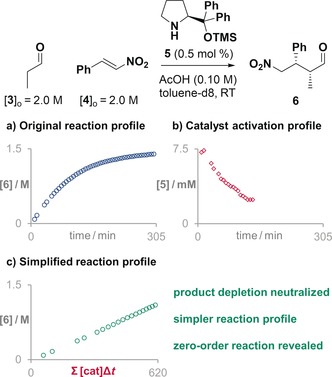
a) The apparent first order of the original reaction profile of an aminocatalyzed Michael addition is corrected by b) the profile of active catalyst to reveal c) a zero‐order reaction profile.

The second kinetic treatment for reactions with activation or deactivation processes serves to estimate the concentration profile of active catalyst when it cannot be measured during the reaction. The amount of active catalyst is estimated by deconvolving its effect on the shape of the reaction profile using VTNA. The VTNA subtracts the kinetic effect of a component from the reaction profile when its concentration to the power of the correct order of the reaction is used to normalize the time scale.^5^ When the time scale is normalized by all the kinetically relevant components of the reaction, whose concentrations change during the reaction, the reaction profile becomes a straight line. Therefore, the amount of catalyst can be estimated by maximizing the linearity of the resulting VTNA profile. This process can be easily performed by many algorithms accessible to all chemists. For generality, we have used the universally available Microsoft Excel add‐in Solver.^11^

To check the viability of our method, we chose the aforementioned reactions, as it was possible to compare the estimated profiles of active catalysts with the measured profiles. In the case of the hydroformylation reaction, there is a clear induction period in the original reaction profile (Figure 4 a). This phenomenon is indicative of the amount of active catalyst building up, so the only constraint imposed on Solver was that the amount of active catalyst could not decrease with time.^8^ To avoid any bias in the solution, the search started from values of 0 % of active catalyst at all time points. The solution automatically found by Solver provided a straight line with R^2^=0.99995 for the progress reaction profile when the time was normalized against the concentration of starting material **1** and that of variable active catalyst (Figure 4 b). As the only evaluation parameter is the straightness of the VTNA plot, the profile of estimated active catalyst has the correct shape but not necessarily the correct magnitude. Therefore, the obtained profile is better presented as a percentage of active catalyst (Figure 4 c). The estimated profile for the activation of the catalyst (red curve in Figure 4 c) is in reasonable agreement with the measured profile (blue curve in Figure 4 c). The small discrepancy after the induction period could be a consequence of measuring the hydride of the catalyst only instead of the total amount of potential catalytic species. Indeed, the estimated profile seems more likely, as the very slow formation of the active catalyst in the last section is not consistent with the profile for a standard kinetic behavior.

**Figure 4:**
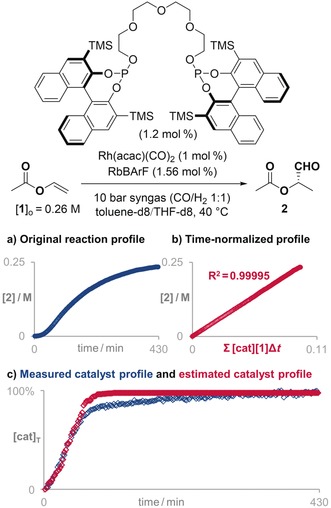
a) The original reaction progress profile becomes b) an almost perfect straight line when applying the VTNA using the estimated catalyst profile. c) The estimated profile of catalyst activation (red points) overlays perfectly with the measured profile (blue points).

The aminocatalytic Michael addition does not reach completion because of catalyst deactivation during the reaction (Figure 5 a). Both same‐excess experiments^12^ and a second addition of fresh catalyst proved that the incomplete reaction was a consequence of catalyst deactivation.^8^ The profile of deactivation was estimated using Solver, starting from 100 % of catalyst at all time points and with the only restriction being that the amount of catalyst could not increase with time. The solution found by Solver converts the original reaction profile (Figure 5 a) into a straight line with R^2^=0.999995 (Figure 5 b) when the time scale is normalized by the amount of estimated active catalyst. The profile of estimated active catalyst against time (red curve in Figure 5 c) is in good agreement with the measured profile (blue curve in Figure 5 c), showing that the approach also works well for estimation of deactivation profiles. Moreover, the estimated profile is able to provide information for the last part of the reaction, when it was impossible to confidently measure the amount of active catalyst experimentally.^8^

**Figure 5:**
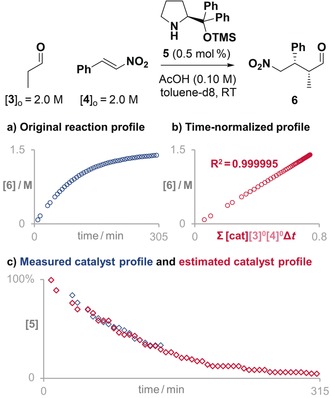
a) The original reaction progress profile becomes b) an almost perfect straight line when applying the VTNA using the estimated catalyst profile. c) The estimated profile of catalyst deactivation (red points) agrees with the measured profile (blue points).

The elucidation of the catalyst deactivation profile for this Michael reaction is possible without knowing the specific deactivation pathways. Nevertheless, we also performed extensive studies using NMR spectroscopy and mass spectrometry to elucidate them. We investigated the desilylation of the aminocatalyst and the interactions of each of the aminocatalytic intermediates with all the reaction components. The structural and kinetic studies^8^ have shown the presence of several deactivation pathways occurring simultaneously; the most important ones being the reactions of the transient zwitterionic iminium nitronate **7** with either propanal (**3**) or *trans*‐*β*‐nitrostyrene (**4**), and the reaction of the catalyst **5** with the side product **10**. The resulting 6‐membered ring cycles (**8**, **9**, and **11**) are stable and inactive, and therefore trap the catalyst out of the catalytic cycle (Figure 6).

**Figure 6:**
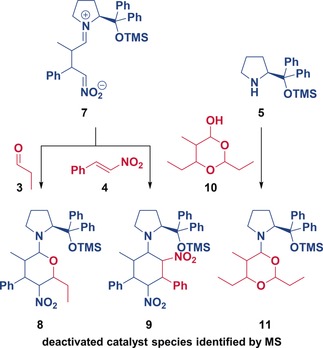
The main pathways of catalyst deactivation are the reactions of the transient zwitterionic iminium nitronate **7** with either propanal (**3**) or *trans*‐β‐nitrostyrene (**4**) and the reaction of the catalyst **5** with the side product **10**. Key: mass spectrometry (MS).

There are some caveats to keep in mind when using VTNA to estimate the temporal profile of active catalyst. The first is that the values of the resulting catalyst profile are relative because the evaluation of the best profiles is based only on the R^2^ value of the resulting VTNA plot. This means that there is an infinite number of profiles with the same shape but different magnitudes that would yield a straight line with an identical R^2^ value. Therefore, the solution found by Solver should be treated as a percentage of the maximum amount of active catalyst at each time, and if a concentration profile is desired, the concentration of active catalyst at one time point must be known. Another caveat is that, if the order of the substances whose concentration is changing during the reaction is not accurate, the VTNA plot, and therefore the estimated profile of active catalyst, will be affected. To minimize this effect, the order of kinetically relevant reactants and catalyst should be known before applying the method.

In conclusion, VTNA is a powerful tool for the treatment of reaction profiles altered by activation and deactivation processes. It serves to simplify the profiles of reactions with variable catalyst concentrations by normalization using the instantaneous catalyst concentration measured throughout the reaction. Also, it provides a satisfactory estimation of the percentages of activated or deactivated catalyst during the reaction by identifying the best linearization of the variable time normalized reaction profile. Both treatments render good results when applied to experimental data—as proven by their application to different reactions. These treatments are useful to facilitate the kinetic analysis of catalytic reactions and obtain accurate and useful mechanistic information.

## Conflict of interest

The authors declare no conflict of interest.

## Supporting information

As a service to our authors and readers, this journal provides supporting information supplied by the authors. Such materials are peer reviewed and may be re‐organized for online delivery, but are not copy‐edited or typeset. Technical support issues arising from supporting information (other than missing files) should be addressed to the authors.

Supplementary

## References

[1] R. H. Crabtree , Chem. Rev. 2015, 115, 127. 25493420 10.1021/cr5004375

[2] Active catalyst refers only to on-cycle catalytic species. Therefore, the presented methods are applicable to catalytic systems regardless of the reversible or irreversible nature of the off-cycle processes.

[3] A. Vidal-Ferran , I. Mon , A. Bauzà , A. Frontera , L. Rovira , Chem. Eur. J. 2015, 21, 11417. 26119347 10.1002/chem.201501441

[4] Y. Hayashi , H. Gotoh , T. Hayashi , M. Shoji , Angew. Chem. Int. Ed. 2005, 44, 4212; 10.1002/anie.200500599 15929151

[5a] J. Burés , Angew. Chem. Int. Ed. 2016, 55, 16084; 10.1002/anie.201609757 27885777

[5b] J. Burés , Angew. Chem. Int. Ed. 2016, 55, 2028; 10.1002/anie.201508983 PMC4797368 26749539

[5c] J. Burés , D.-T. C. Nielsen , Chem. Sci. 2019, 10, 348. 30746083 10.1039/c8sc04698k PMC6335952

[6a] A. C. Brezny , C. R. Landis , J. Am. Chem. Soc. 2017, 139, 2778; 28125782 10.1021/jacs.6b12533

[6b] N. J. Beacha , S. M. M. Knappa , C. R. Landis , Rev. Sci. Instrum. 2015, 86, 104101. 26520969 10.1063/1.4932676

[7a] D. A. Foley , E. Bez , A. Codina , K. L. Colson , M. Fey , R. Krull , D. Piroli , M. T. Zell , B. L. Marquez , Anal. Chem. 2014, 86, 12008; 25375410 10.1021/ac502300q

[7b] http://www.bruker.com/InsightMR, accessed 27 Oct **2018**.

[8] See the Supporting Information.

[9] Rhodium Catalyzed Hydroformylation (Eds.: P. W. N. M. van Leeuwen, C. Claver), Kluwer Academic Publishers, Dordrecht, 2000, pp. 70–72.

[10a] J. Burés , A. Armstrong , A. D. G. Blackmond , J. Am. Chem. Soc. 2011, 133, 8822; 21568340 10.1021/ja203660r

[10b] K. Patora-Komisarska , M. Benohoud , H. Ishikawa , D. Seebach , Y. Hayashi , Helv. Chim. Acta 2011, 94, 719;

[10c] J. Burés , A. Armstrong , A. D. G. Blackmond , J. Am. Chem. Soc. 2012, 134, 6741; 22452319 10.1021/ja300415t

[10d] J. Burés , A. Armstrong , A. D. G. Blackmond , J. Am. Chem. Soc. 2012, 134, 14264; 10.1021/ja300415t 22452319

[10e] D. Seebach , X. Sun , M.-O. Ebert , W. B. Schweizer , N. Purkayastha , A. K. Beck , J. Duschmalé , H. Wennemers , T. Mukaiyama , M. Benohoud , Y. Hayashi , M. Reiher , Helv. Chim. Acta 2013, 96, 799;

[10f] J. Burés , A. Armstrong , A. D. G. Blackmond , Acc. Chem. Res. 2016, 49, 214. 26830669 10.1021/acs.accounts.5b00394

[11] S. Walsh , D. Diamond , Talanta 1995, 42, 561. 18966265 10.1016/0039-9140(95)01446-i

[12] D. G. Blackmond , Angew. Chem. Int. Ed. 2005, 44, 4302; 10.1002/anie.200462544 15997457

