# Supplementary material for: Kinetic Treatments for Catalyst Activation and Deactivation Processes based on Variable Time Normalization Analysis
Source: Angew Chem Int Ed Engl. 2019 Jun 7;58(30):10189–93. doi: 10.1002/anie.201903878 (PMC6772004; doi:10.1002/anie.201903878)
Supplement: Supplementary file 1 — Supplementary [file ANIE-58-10189-s001.pdf]

Angewandte Chemie
Eine Zeitschrift der Gesellschaft Deutscher Chemiker

Supporting Information

Kinetic Treatments for Catalyst Activation and Deactivation Processes based on Variable Time Normalization Analysis

Alicia Martínez-Carrión, Michael G. Howlett, Carla Alamillo-Ferrer, Adam D. Clayton, Richard A. Bourne, Anna Codina, Anton Vidal-Ferran, Ralph W. Adams,* and Jordi Burés**

anie_201903878_sm_miscellaneous_information.pdf

Author Contributions

A.M. Investigation: Lead; Writing—Original Draft: Supporting; Writing—Review & Editing: Equal
M.H. Investigation: Lead; Writing—Original Draft: Supporting; Writing—Review & Editing: Equal
C.A. Investigation: Lead; Writing—Original Draft: Supporting; Writing—Review & Editing: Equal
A.C. Investigation: Supporting; Resources: Equal; Writing—Review & Editing: Equal
R.B. Funding acquisition: Lead; Supervision: Lead; Writing—Review & Editing: Equal
A.C. Funding acquisition: Supporting; Methodology: Supporting; Resources: Lead; Writing—Original Draft: Supporting; Writing—Review & Editing: Equal
A.V. Conceptualization: Supporting; Funding acquisition: Lead; Investigation: Supporting; Supervision: Lead; Writing—Original Draft: Supporting; Writing—Review & Editing: Equal
R.A. Conceptualization: Supporting; Investigation: Supporting; Resources: Lead; Supervision: Lead; Writing—Original Draft: Supporting; Writing—Review & Editing: Equal
J.B. Conceptualization: Lead; Data curation: Lead; Formal analysis: Lead; Funding acquisition: Lead; Investigation: Lead; Methodology: Lead; Resources: Lead; Supervision: Lead; Writing—Original Draft: Lead; Writing—Review & Editing: Equal.

Table of Contents:

1. General remarks S-2
2. Hydroformylation reaction (Figure 2 and 4) S-2
    2.1. Experimental setup S-2
    2.2. Preparation of the precursors of the catalyst S-3
    2.3. Hydroformylation procedure S-3
    2.4. NMR spectra S-4
    2.5. Kinetic data (Figure 2 and 4) S-8
3. Aminocatalytic Michael addition (Figure 3, 5 and 6) S-19
    3.1. Experimental setup S-19
    3.2. NMR spectra S-20
    3.3. Kinetic data (Figure 3 and 5) S-23
    3.4. Study of catalyst deactivation pathways (Figure 6) S-27
4. References S-65

1. General remarks

NMR spectra were recorded on a Bruker AVII 500 MHz spectrometer, Bruker AVIII HD 400 MHz spectrometer with BBO prodigy probe and a Bruker AVIII HD 800 spectrometer. ^{1}H-NMR chemicals shifts (δ) are quoted in ppm relative to residual solvent peaks (Toluene-d8: 2.08 ppm, $CDCl_3$: 7.26 ppm, THF-d8: 1.72 ppm). ^{13}C-NMR chemicals shifts (δ) are quoted in ppm relative to residual solvent peaks (Toluene-d8: 20.43 ppm, $CDCl_3$: 77.16 ppm, THF-d8: 25.31 ppm).

HPLC were recorded on a Thermo Scientific UHPLC Dionex Ultimate 3000. Mass Spec were recroded in an Exactive Plus EMR (Extended Mass Range) Orbitrap using HESI method in positive mode.

2. Hydroformylation reaction

2.1. Experimental setup

Hydroformylation reactions were carried out in a 25 mL 316SS autoclave from Berghof equipped with inlet and outlet sample lines. Sample circulation was carried out using a HPLC pump (JASCO-PU-1580). The temperature of the reaction mixture was stabilized and kept constant with a Cole-Parmer Polystat Refrigerated Circulating Bath. The hydroformylation mixture was circulated into the spectrometer using an InsightMR flow tube (4 meters) from Bruker. The syngas cylinder (CO/H_2 1:1 ratio) and the pressure regulator was purchased from BOC Group. Fluorinated ethylene propylene (FEP) tubing was used ((IDEX-Upchurch Product #1692: FEP Capillary 1/32" OD (0.80 mm) x 0.016" ID (0.405 mm))) and the length of tubing connecting the reactor and pump to the spectrometer was minimized. The FEP tubing is a non-reactive material and offers chemical and mechanical stability (up to 50 °C, up to 121 bar) along with low gas permeability and good flexibility. Fittings used in the system were stainless steel from Swagelok or Upchurch PEEK (polyetherether ketone) from Tecknocroma.

All the equipment was positioned 0.5 m outside the 5 Gauss line of the spectrometer's magnet.

The high-pressure flow system configuration used for hydroformylation reactions is shown in Fig. S1. A 25 mL Berghof autoclave equipped with inlet and outlet sample lines (blue lines) was used. The reaction mixture was circulated from the reactor to the HPLC pump, then to the InsightMR flow tube and back to the reactor employing FEP tubing as sample lines. The temperature was kept constant throughout the whole system (*i.e.*, reactor, spectrometer and InsightMR flow tube). The reactor was connected to the high-pressure cylinder using stainless tubing (black lines), Swagelok fittings and valves. The design of the system allowed both the pressurization with different gases (N_2 and syngas) and depressurization by connecting the system to an exhaust. Due to the toxicity and flammability of syngas, CO and H_2 detectors were placed in the vicinity of the reactor, syngas cylinder and exhaust.

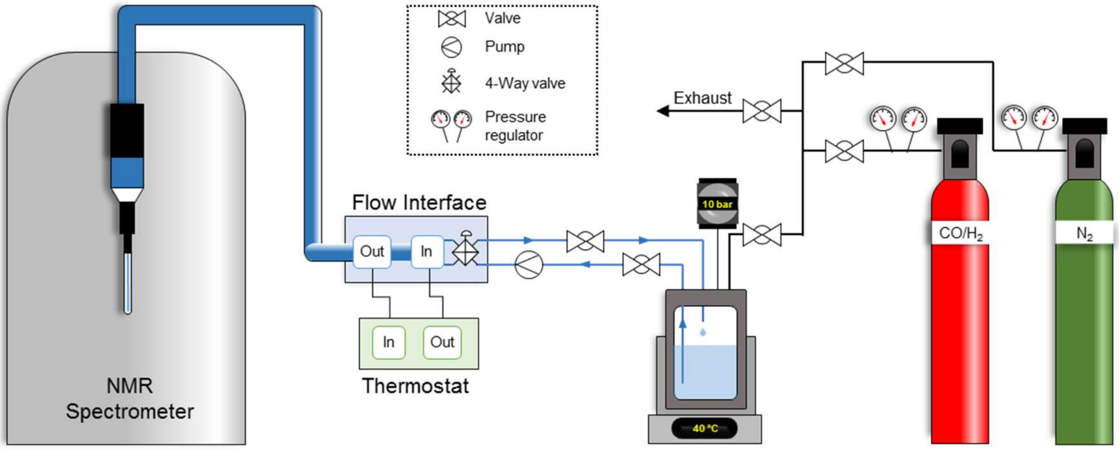

Figure S1. Experimental setup for on-line monitoring of high-pressure reactions.

2.2. Preparation of the precursors of the catalyst

Bisphosphite ligand **L1** and RbBArF were synthesized following the procedures described in the literature and their spectroscopic data were in agreement with those reported. [1],[2]

2.3. Hydroformylation procedure

In an argon-filled glove box, the following solutions were prepared:

<u>Solution A</u>: ligand **L1** (52 mg, 46.8 µmol), RbBArF (58 mg, 60.8 µmol) in 450 µL of THF-d8, $[Rh(acac)(CO)_2]$ (10 mg, 39 µmol) and toluene-d8 were mixed until a total volume of 10 mL.

<u>Solution B</u>: vinyl acetate **1** (336 mg, 3.9 mmol), dodecane (199 mg, 1.17 mmol) and toluene-d8 were mixed until a total volume of 5 mL.

For the hydroformylation reactions, the corresponding temperature value (40 °C) was set in the thermostat, NMR spectrometer and heating plate. The system was purged three times with 2 bar of N_2. With a positive flow of N_2 (1 bar) in the reactor, the sample inlet valve was opened and solution A was injected followed by solution B. The stirring rate was set at 800 rpm. The reaction mixture was circulated under nitrogen at 5 mL/min for 5 min. NMR spectra were recorded while flowing under nitrogen atmosphere. The system was purged three times with 10 bar of syngas (CO/H_2 1:1 ratio) and the syngas cylinder was left open during the reaction monitoring. Once the experiment was finished, the syngas cylinder was closed and the reactor was depressurized. The thermostat, NMR spectrometer and heating plate were switched off, the crude mixture was removed from the reactor and the sample lines were cleaned. For sample lines cleaning, acetone was injected at 5 mL/min followed by air flushing until complete dryness.

Enantioselectivity was determined by GC analysis, performed on an Agilent 6890N equipped with a FID detector using chiral stationary phases. GC analysis of the crude mixture confirmed the already reported regio- and enantioselectivity.[1]

2.4. NMR spectra

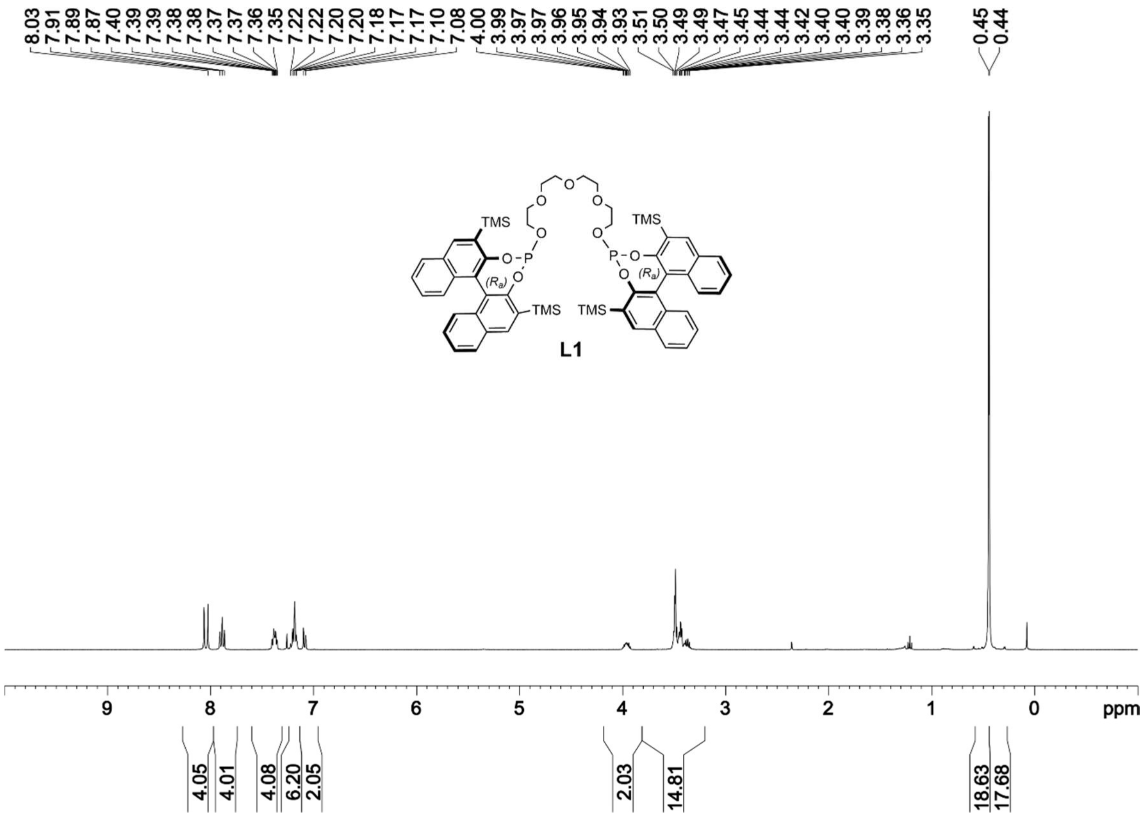

Figure S2. ^{1}H NMR spectrum (500 MHz, $CDCl_3$) of ligand **L1**.

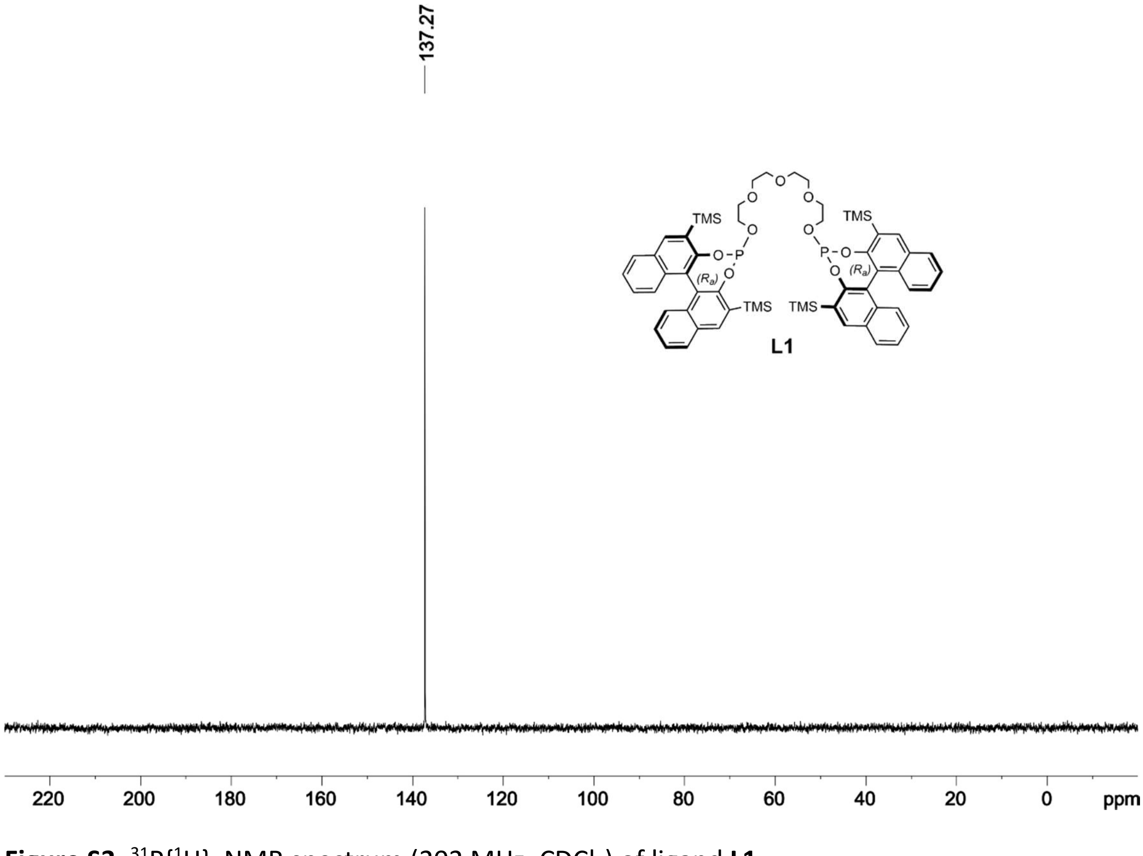

Figure S3. ^{31}P{^{1}H} NMR spectrum (202 MHz, $CDCl_3$) of ligand **L1**.

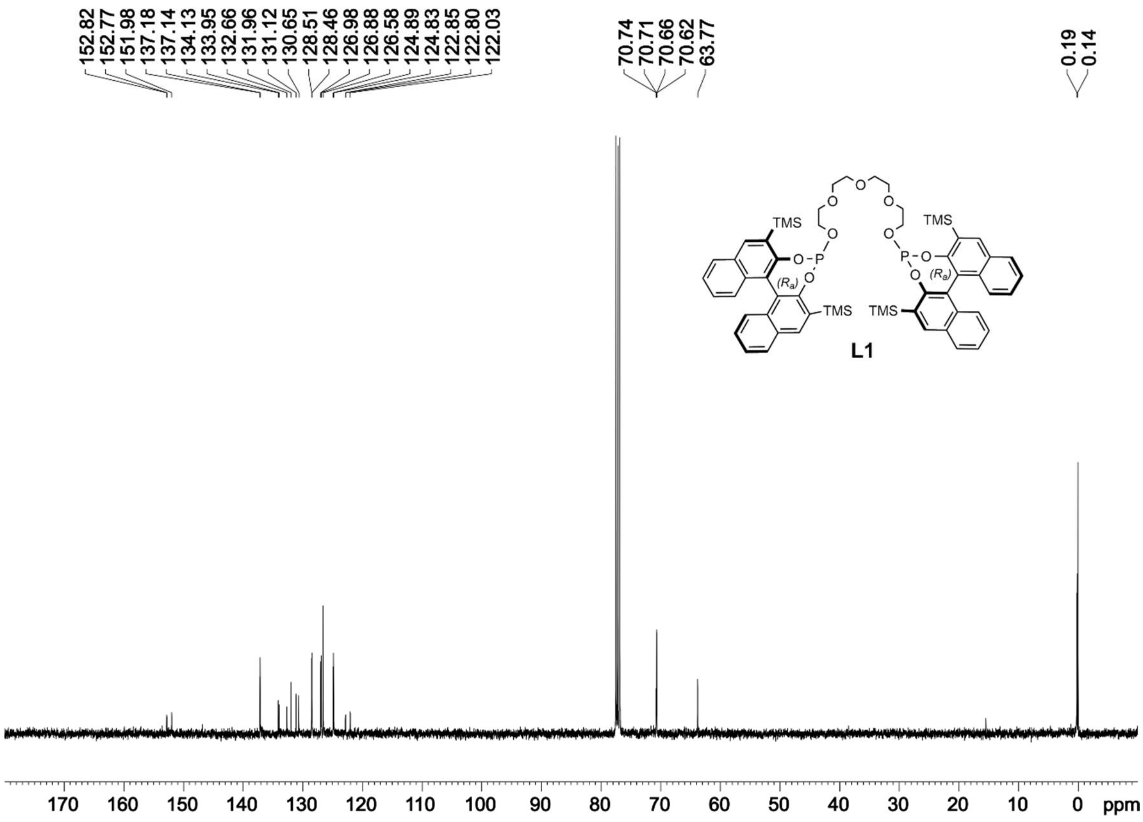

Figure S4. $^{13}C\{^1H\}$ NMR spectrum (125 MHz, $CDCl_3$) of ligand **L1**.

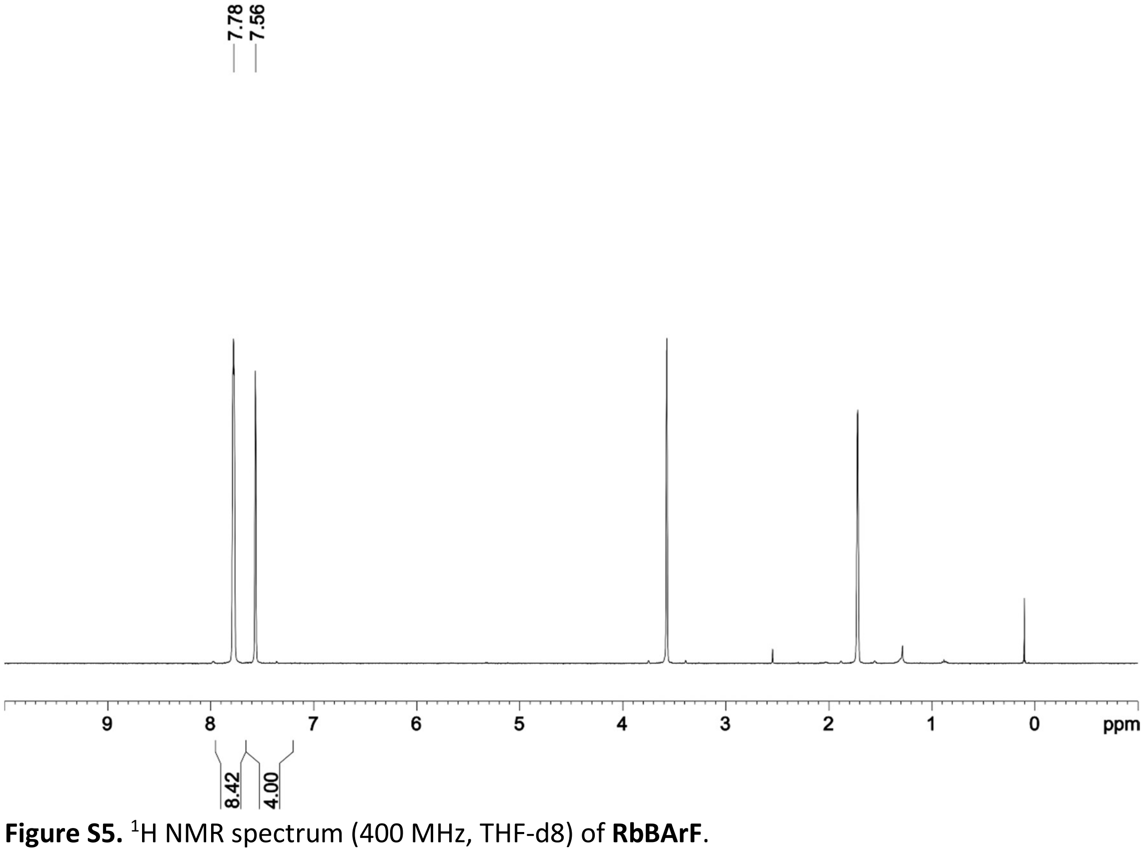

Figure S5. ^1H NMR spectrum (400 MHz, THF-d8) of **RbBArF**.

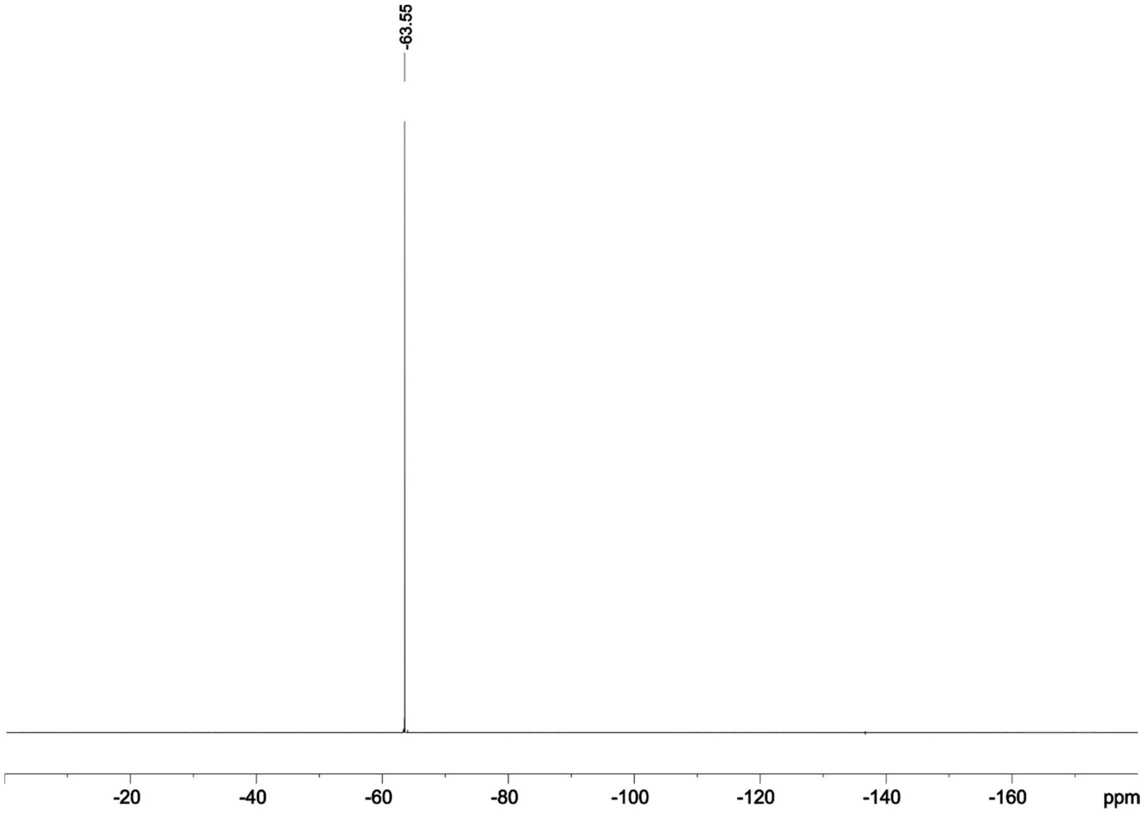

Figure S6. $^{19}F\{^{1}H\}$ NMR spectrum (376 MHz, THF-d8) of **RbBArF**.

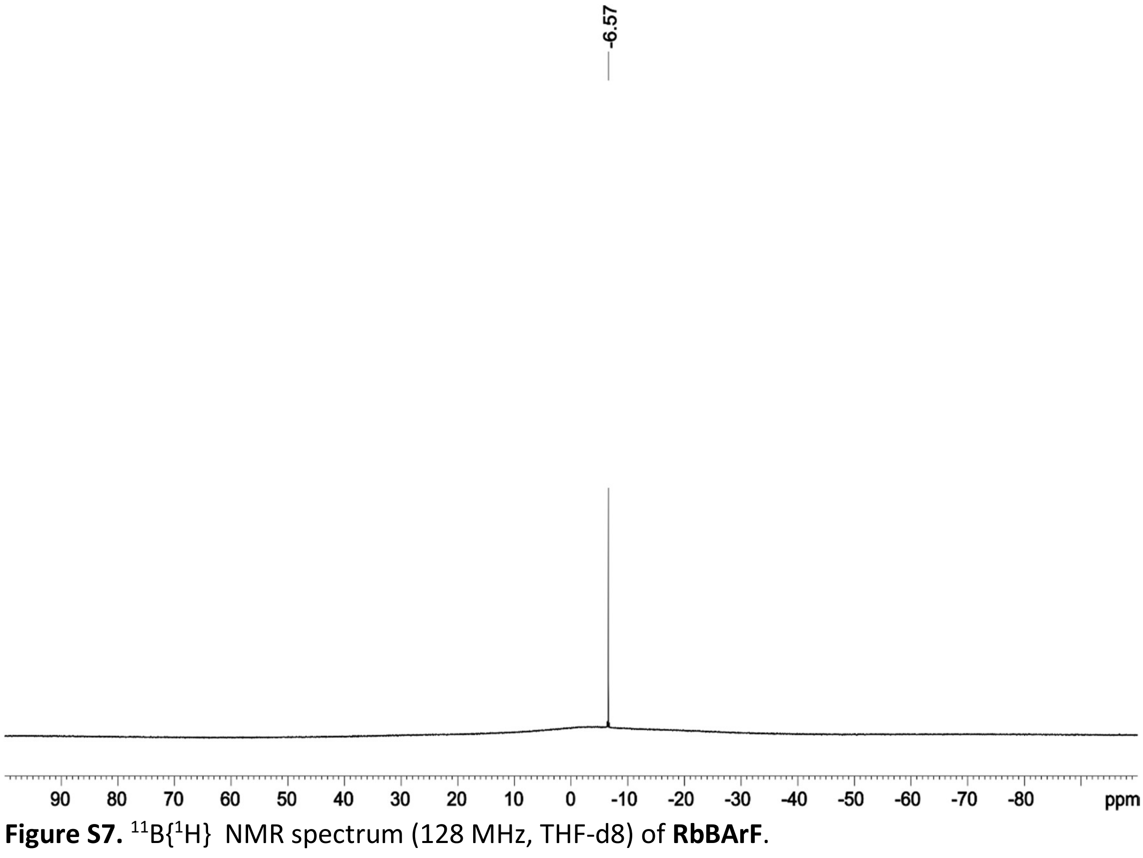

Figure S7. $^{11}B\{^{1}H\}$ NMR spectrum (128 MHz, THF-d8) of **RbBArF**.

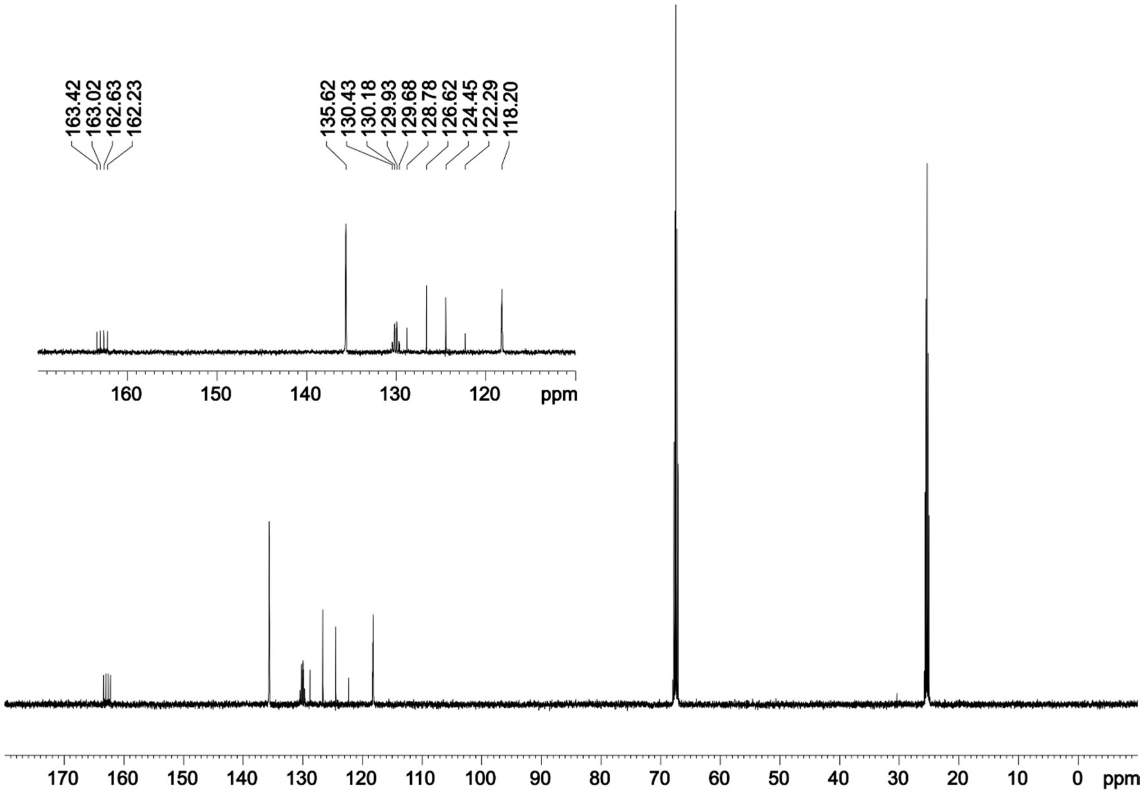

Figure S8. $^{13}C\{^{1}H\}$ NMR spectrum (101 MHz, THF-d8) of **RbBArF**.

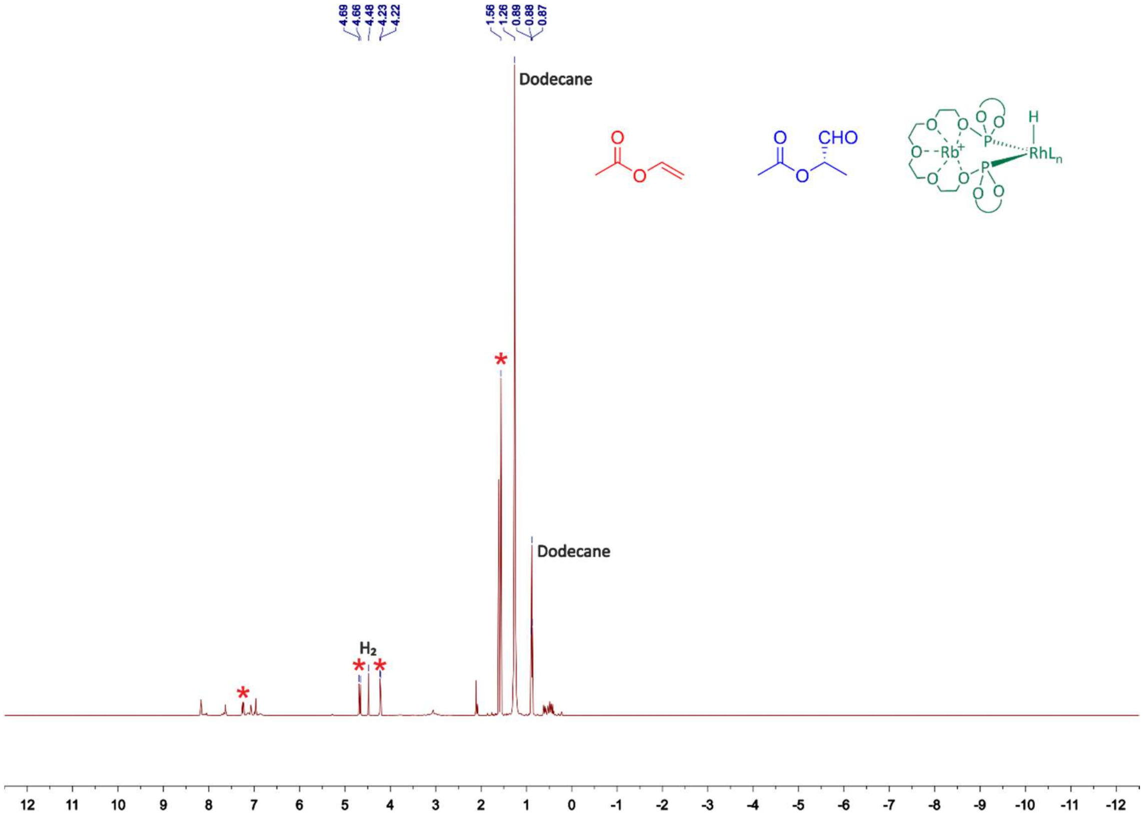

Figure S9. ^{1}H NMR spectrum (500 MHz, toluene-d8) of the hydroformylation reaction after 10 minutes.

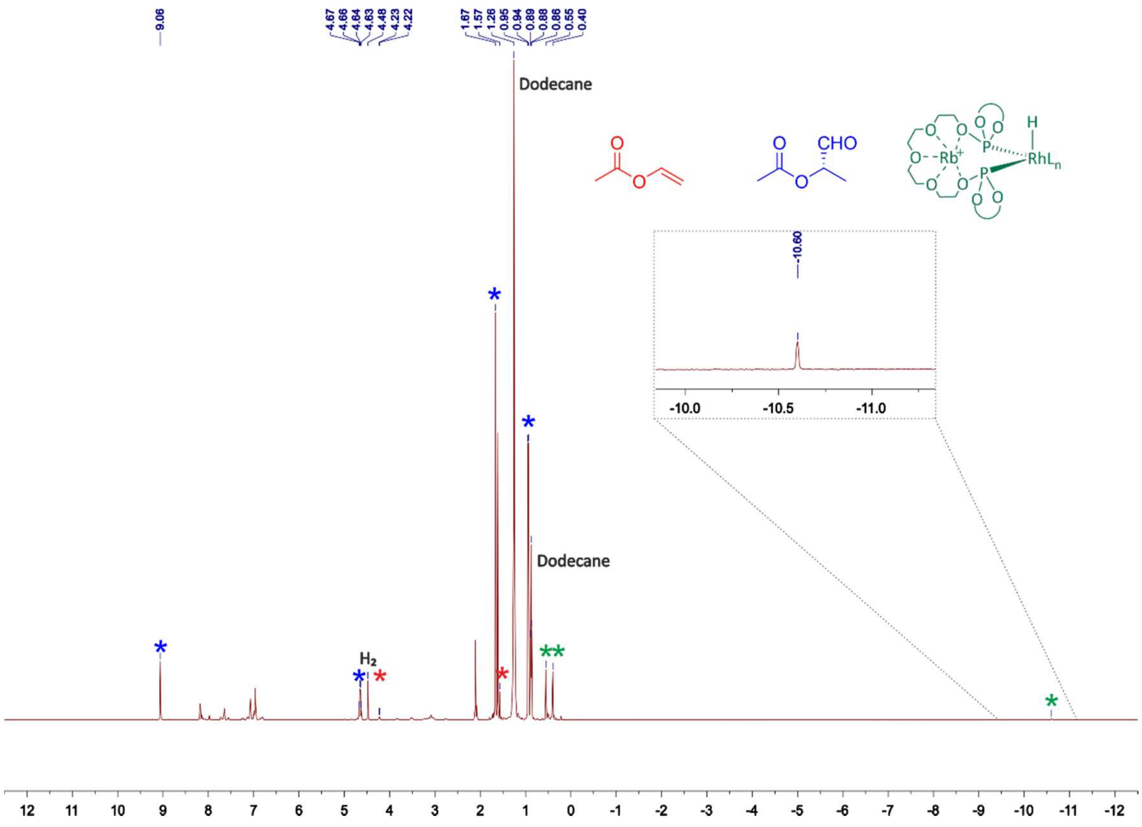

Figure S10. ^1H NMR spectrum (500 MHz, toluene-d8) of the hydroformylation reaction after 426 minutes.

2.5. Kinetic data (Figure 2 and 4)

Figure 2a and 2b of the manuscript show respectively the concentration of product and catalyst obtained from the integration of consecutive ^1H-NMR spectra. The numerical values for both figures are shown in Table S1.

Figure 2c shows the reaction profile corrected by the amount of active catalyst measured as a hydride of the supramolecular rhodium complex at each time point. The time values shown in Table S1 have been calculated using the VTNA formula:

$$normalized\ time = \sum_{i=1}^{n} \left(\frac{[\mathrm{RhH}]_i + [\mathrm{RhH}]_{i-1}}{2} \right)^1 (t_i - t_{i-1})$$

Table S1. Data for Figure 2.

time / min	[P] / M	[RhH] / mM	[SM]=[SM]$_o$-[P] / M	VTNA
2.150	0.000357	0.006481	0.259643	0.000014
4.283	0.000568	0.062713	0.259432	0.000088
6.417	0.000298	0.051924	0.259702	0.000210
8.567	0.000985	0.150575	0.259015	0.000428
10.700	0.001087	0.166071	0.258913	0.000765
12.850	0.001456	0.260574	0.258544	0.001224

14.983	0.001521	0.324562	0.258479	0.001848
17.133	0.002191	0.377941	0.257809	0.002603
19.267	0.002524	0.449942	0.257476	0.003487
21.400	0.003352	0.572287	0.256648	0.004577
23.550	0.003955	0.631517	0.256045	0.005871
25.683	0.004546	0.676197	0.255454	0.007266
27.833	0.005455	0.777741	0.254545	0.008829
29.967	0.006412	0.896483	0.253588	0.010615
32.100	0.007683	0.884364	0.252317	0.012514
34.250	0.009242	1.021546	0.250758	0.014563
36.383	0.010007	1.138909	0.249993	0.016868
38.533	0.012146	1.177914	0.247854	0.019358
40.667	0.013443	1.251882	0.246557	0.021950
42.817	0.015104	1.344928	0.244896	0.024742
44.950	0.017124	1.457053	0.242876	0.027730
47.100	0.018905	1.457003	0.241095	0.030863
49.233	0.021093	1.527969	0.238907	0.034047
51.367	0.022968	1.584891	0.237032	0.037367
53.517	0.025094	1.676899	0.234906	0.040874
55.650	0.027647	1.729469	0.232353	0.044507
57.800	0.029931	1.749759	0.230069	0.048247
59.933	0.032300	1.877047	0.227700	0.052116
62.083	0.034772	1.892712	0.225228	0.056168
64.217	0.037141	1.906997	0.222859	0.060221
66.367	0.039678	1.952342	0.220322	0.064370
68.500	0.042305	1.966563	0.217695	0.068550
70.633	0.044899	2.017056	0.215101	0.072800
72.783	0.047556	2.054739	0.212444	0.077177
74.917	0.050393	2.052759	0.209607	0.081558
77.067	0.052960	2.075148	0.207040	0.085996
79.200	0.055157	2.081308	0.204843	0.090429
81.333	0.057943	2.099322	0.202057	0.094888
83.483	0.060690	2.125603	0.199310	0.099430
85.617	0.063263	2.098041	0.196737	0.103936
87.767	0.065631	2.143164	0.194369	0.108495
89.900	0.068296	2.145193	0.191704	0.113069
92.050	0.070555	2.156997	0.189445	0.117694
94.183	0.073456	2.153356	0.186544	0.122292
96.333	0.075474	2.108371	0.184526	0.126873
98.467	0.078088	2.183690	0.181912	0.131451

100.600	0.079923	2.135618	0.180077	0.136058
102.750	0.082403	2.181242	0.177597	0.140699
104.883	0.084941	2.168216	0.175059	0.145338
107.033	0.087076	2.155352	0.172924	0.149986
109.167	0.089235	2.209565	0.170765	0.154642
111.317	0.092106	2.176811	0.167894	0.159358
113.450	0.093527	2.165135	0.166473	0.163989
115.583	0.095973	2.230476	0.164027	0.168678
117.733	0.097846	2.194052	0.162154	0.173434
119.867	0.100485	2.202985	0.159515	0.178124
122.017	0.102237	2.213808	0.157763	0.182872
124.150	0.103985	2.212425	0.156015	0.187594
126.300	0.106077	2.191602	0.153923	0.192328
128.433	0.108398	2.204654	0.151602	0.197017
130.583	0.110128	2.204077	0.149872	0.201757
132.717	0.111861	2.220534	0.148139	0.206476
134.850	0.114196	2.242340	0.145804	0.211237
137.000	0.116354	2.215293	0.143646	0.216029
139.133	0.117874	2.222584	0.142126	0.220762
141.283	0.120037	2.306519	0.139963	0.225631
143.417	0.121543	2.250504	0.138457	0.230492
145.550	0.123050	2.252581	0.136950	0.235295
147.700	0.125374	2.251389	0.134626	0.240137
149.833	0.127127	2.260964	0.132873	0.244950
151.983	0.128497	2.267592	0.131503	0.249818
154.117	0.130581	2.292608	0.129419	0.254683
156.267	0.132050	2.242682	0.127950	0.259558
158.400	0.133727	2.273747	0.126273	0.264375
160.550	0.135180	2.266756	0.124820	0.269257
162.683	0.137242	2.254386	0.122758	0.274079
164.817	0.138715	2.269060	0.121285	0.278904
166.967	0.140692	2.276345	0.119308	0.283790
169.100	0.142299	2.291865	0.117701	0.288663
171.250	0.143960	2.282368	0.116040	0.293580
173.383	0.145503	2.297578	0.114497	0.298466
175.533	0.146776	2.302777	0.113224	0.303411
177.667	0.148873	2.342390	0.111127	0.308366
179.800	0.150139	2.336172	0.109861	0.313356
181.950	0.151782	2.338902	0.108218	0.318382
184.083	0.153003	2.313500	0.106997	0.323345

186.233	0.154432	2.270176	0.105568	0.328272
188.367	0.156017	2.353802	0.103983	0.333204
190.517	0.157841	2.345430	0.102159	0.338256
192.650	0.158346	2.337109	0.101654	0.343251
194.783	0.159595	2.339901	0.100405	0.348240
196.933	0.160933	2.357443	0.099067	0.353289
199.067	0.161709	2.343412	0.098291	0.358303
201.217	0.163275	2.309745	0.096725	0.363306
203.350	0.164548	2.333950	0.095452	0.368259
205.500	0.165805	2.366876	0.094195	0.373312
207.633	0.167151	2.354671	0.092849	0.378349
209.767	0.168543	2.422389	0.091457	0.383444
211.917	0.169119	2.352762	0.090881	0.388577
214.050	0.170561	2.366078	0.089439	0.393611
216.200	0.171992	2.373630	0.088008	0.398706
218.333	0.172892	2.335911	0.087108	0.403730
220.483	0.174221	2.311668	0.085779	0.408726
222.617	0.175369	2.298956	0.084631	0.413644
224.750	0.176829	2.426153	0.083171	0.418684
226.900	0.177750	2.324879	0.082250	0.423791
229.033	0.178858	2.373821	0.081142	0.428803
231.183	0.179752	2.411065	0.080248	0.433947
233.317	0.181182	2.407153	0.078818	0.439086
235.467	0.181935	2.388840	0.078065	0.444242
237.600	0.183135	2.341245	0.076865	0.449287
239.750	0.184299	2.381038	0.075701	0.454364
241.883	0.185060	2.345446	0.074940	0.459405
244.017	0.186140	2.372371	0.073860	0.464438
246.167	0.187256	2.383025	0.072744	0.469550
248.300	0.188415	2.416708	0.071585	0.474670
250.450	0.188978	2.352474	0.071022	0.479796
252.583	0.189702	2.349117	0.070298	0.484811
254.717	0.190784	2.386360	0.069216	0.489863
256.867	0.191679	2.405502	0.068321	0.495014
259.000	0.192614	2.376003	0.067386	0.500114
261.150	0.193541	2.400904	0.066459	0.505249
263.283	0.194629	2.451125	0.065371	0.510425
265.433	0.195387	2.434252	0.064613	0.515677
267.600	0.196083	2.431846	0.063917	0.520948
269.733	0.196910	2.423850	0.063090	0.526128

271.883	0.197671	2.431066	0.062329	0.531347
274.017	0.198591	2.404978	0.061409	0.536505
276.150	0.199222	2.456083	0.060778	0.541690
278.300	0.200447	2.388598	0.059553	0.546898
280.433	0.200859	2.423224	0.059141	0.552031
282.583	0.201801	2.443747	0.058199	0.557263
284.717	0.202213	2.392027	0.057787	0.562421
286.867	0.203172	2.364342	0.056828	0.567534
289.000	0.203882	2.409773	0.056118	0.572627
291.150	0.204548	2.466708	0.055452	0.577869
293.283	0.204977	2.417643	0.055023	0.583079
295.433	0.205658	2.408473	0.054342	0.588267
297.567	0.206498	2.447222	0.053502	0.593446
299.717	0.207362	2.484563	0.052638	0.598748
301.850	0.208179	2.487277	0.051821	0.604051
304.000	0.208613	2.463801	0.051387	0.609374
306.133	0.209628	2.448693	0.050372	0.614614
308.283	0.209960	2.487182	0.050040	0.619920
310.417	0.210577	2.410350	0.049423	0.625144
312.550	0.211308	2.452700	0.048692	0.630331
314.700	0.211829	2.462045	0.048171	0.635614
316.833	0.212710	2.446185	0.047290	0.640850
318.983	0.213131	2.429901	0.046869	0.646092
321.117	0.213754	2.443023	0.046246	0.651289
323.250	0.214297	2.446746	0.045703	0.656505
325.400	0.214922	2.480027	0.045078	0.661801
327.533	0.215684	2.432884	0.044316	0.667042
329.683	0.216625	2.471916	0.043375	0.672314
331.817	0.216868	2.472383	0.043132	0.677588
333.967	0.217630	2.447244	0.042370	0.682877
336.100	0.218103	2.429895	0.041897	0.688079
338.233	0.218636	2.457033	0.041364	0.693292
340.383	0.219466	2.427080	0.040534	0.698542
342.517	0.219929	2.491924	0.040071	0.703789
344.667	0.220535	2.464392	0.039465	0.709117
346.800	0.220942	2.407909	0.039058	0.714314
348.933	0.221967	2.468146	0.038033	0.719516
351.083	0.222135	2.457182	0.037865	0.724810
353.217	0.223008	2.433621	0.036992	0.730027
355.367	0.223170	2.443044	0.036830	0.735270

357.500	0.223722	2.477595	0.036278	0.740518
359.650	0.223767	2.512934	0.036233	0.745883
361.783	0.224226	2.434309	0.035774	0.751160
363.917	0.224596	2.454163	0.035404	0.756375
366.067	0.224852	2.540064	0.035148	0.761743
368.200	0.225271	2.524973	0.034729	0.767146
370.350	0.225361	2.553587	0.034639	0.772605
372.483	0.226064	2.468867	0.033936	0.777963
374.633	0.226124	2.377353	0.033876	0.783172
376.767	0.226746	2.496774	0.033254	0.788372
378.917	0.227128	2.442120	0.032872	0.793681
381.050	0.227325	2.459780	0.032675	0.798910
383.200	0.227922	2.493495	0.032078	0.804234
385.333	0.228451	2.521839	0.031549	0.809584
387.467	0.228700	2.482888	0.031300	0.814922
389.617	0.229053	2.434806	0.030947	0.820209
391.750	0.229429	2.496186	0.030571	0.825469
393.900	0.229940	2.413707	0.030060	0.830747
396.033	0.230397	2.478858	0.029603	0.835965
398.183	0.230478	2.440024	0.029522	0.841253
400.317	0.230754	2.495482	0.029246	0.846518
402.450	0.230974	2.502520	0.029026	0.851849
404.600	0.231158	2.440775	0.028842	0.857163
406.733	0.231378	2.514522	0.028622	0.862449
408.883	0.231777	2.523651	0.028223	0.867865
411.033	0.232300	2.502749	0.027700	0.873268
413.167	0.232485	2.515137	0.027515	0.878621
415.300	0.232825	2.486234	0.027175	0.883955
417.450	0.233121	2.473302	0.026879	0.889287
419.583	0.233611	2.516704	0.026389	0.894609
421.733	0.233848	2.513270	0.026152	0.900017
423.867	0.233809	2.535449	0.026191	0.905402
426.000	0.233458	2.543003	0.026542	0.910819
428.150	0.233575	2.494818	0.026425	0.916235

Figure 4a shows the original temporal profile of the concentration of product obtained from the integration of consecutive ^{1}H-NMR spectra and the corresponding data is shown in Table S2.

Figure 4b shows the linearized VTNA profile resulting from the normalization of the time scale by the concentration of starting material **1** (vinyl acetate) and the concentration of estimated active catalyst. All the corresponding numerical data is shown in Table S2.

Figure 4c shows the comparison of the estimated temporal profile of percentage of catalyst and the original concentration measured from the ^{1}H-NMR spectra. The values for the estimated concentration profile were found using Solver. In this case, we imposed the condition that the concentration of catalyst could not decrease during the reaction. To do so in Excel, the concentration of catalyst at a given time was defined as the concentration in the previous time plus a given increment. Specifically, these increment values represented the 'Variable Cells' changed by Solver in order to maximize the R^2 (SQR function: square of the Pearson product moment correlation coefficient through data points (concentration of product, normalized time)) of the resulting VTNA profile shown in Figure 4b.

Table S2. Data for Figure 4.

**time / min**	**[P] / M**	estimated [active cat] / mM	% estimated active catalyst	% measured RhH	VTNA using estimated [active catalyst]
2.150	0.000357	0.001256	0.05	0.25	0.000000
4.283	0.000568	0.033431	1.31	2.46	0.000010
6.417	0.000298	0.141336	5.53	2.03	0.000058
8.567	0.000985	0.186178	7.29	5.90	0.000149
10.700	0.001087	0.189802	7.43	6.50	0.000253
12.850	0.001456	0.236077	9.24	10.20	0.000372
14.983	0.001521	0.320082	12.53	12.71	0.000525
17.133	0.002191	0.362538	14.20	14.80	0.000714
19.267	0.002524	0.434342	17.01	17.62	0.000933
21.400	0.003352	0.474824	18.59	22.41	0.001183
23.550	0.003955	0.502241	19.67	24.73	0.001452
25.683	0.004546	0.569087	22.29	26.48	0.001744
27.833	0.005455	0.629314	24.64	30.46	0.002073
29.967	0.006412	0.769377	30.13	35.11	0.002452
32.100	0.007683	0.958992	37.55	34.63	0.002918
34.250	0.009242	1.021600	40.01	40.00	0.003454
36.383	0.010007	1.175246	46.02	44.60	0.004040
38.533	0.012146	1.185522	46.43	46.13	0.004672
40.667	0.013443	1.251378	49.00	49.02	0.005315
42.817	0.015104	1.406698	55.09	52.67	0.006017
44.950	0.017124	1.489075	58.31	57.06	0.006770
47.100	0.018905	1.569862	61.48	57.06	0.007566
49.233	0.021093	1.613991	63.20	59.84	0.008381
51.367	0.022968	1.649038	64.58	62.07	0.009209
53.517	0.025094	1.753467	68.67	65.67	0.010072
55.650	0.027647	1.822837	71.38	67.73	0.010964
57.800	0.029931	1.916824	75.06	68.52	0.011893
59.933	0.032300	1.983209	77.66	73.51	0.012845
62.083	0.034772	2.167532	84.88	74.12	0.013856

64.217	0.037141	2.218107	86.86	74.68	0.014904
66.367	0.039678	2.280643	89.31	76.45	0.015975
68.500	0.042305	2.286054	89.52	77.01	0.017042
70.633	0.044899	2.290337	89.69	78.99	0.018099
72.783	0.047556	2.296412	89.93	80.46	0.019153
74.917	0.050393	2.374631	92.99	80.39	0.020204
77.067	0.052960	2.394171	93.76	81.26	0.021272
79.200	0.055157	2.403436	94.12	81.51	0.022326
81.333	0.057943	2.414251	94.54	82.21	0.023371
83.483	0.060690	2.454987	96.14	83.24	0.024422
85.617	0.063263	2.456780	96.21	82.16	0.025459
87.767	0.065631	2.457195	96.23	83.93	0.026492
89.900	0.068296	2.457389	96.23	84.01	0.027504
92.050	0.070555	2.467363	96.62	84.47	0.028513
94.183	0.073456	2.467605	96.63	84.33	0.029503
96.333	0.075474	2.472968	96.84	82.57	0.030488
98.467	0.078088	2.477392	97.02	85.51	0.031456
100.600	0.079923	2.480014	97.12	83.63	0.032413
102.750	0.082403	2.481087	97.16	85.42	0.033367
104.883	0.084941	2.481106	97.16	84.91	0.034300
107.033	0.087076	2.482383	97.21	84.40	0.035228
109.167	0.089235	2.493052	97.63	86.53	0.036140
111.317	0.092106	2.494176	97.67	85.25	0.037048
113.450	0.093527	2.494818	97.70	84.79	0.037938
115.583	0.095973	2.494818	97.70	87.35	0.038817
117.733	0.097846	2.494818	97.70	85.92	0.039692
119.867	0.100485	2.494818	97.70	86.27	0.040548
122.017	0.102237	2.494818	97.70	86.69	0.041399
124.150	0.103985	2.494818	97.70	86.64	0.042234
126.300	0.106077	2.494818	97.70	85.82	0.043065
128.433	0.108398	2.494818	97.70	86.34	0.043878
130.583	0.110128	2.494818	97.70	86.31	0.044687
132.717	0.111861	2.494818	97.70	86.96	0.045480
134.850	0.114196	2.494818	97.70	87.81	0.046262
137.000	0.116354	2.494818	97.70	86.75	0.047038
139.133	0.117874	2.494818	97.70	87.04	0.047799
141.283	0.120037	2.494818	97.70	90.32	0.048555
143.417	0.121543	2.494818	97.70	88.13	0.049296
145.550	0.123050	2.494818	97.70	88.21	0.050029
147.700	0.125374	2.494818	97.70	88.17	0.050758

149.833	0.127127	2.494818	97.70	88.54	0.051469
151.983	0.128497	2.494818	97.70	88.80	0.052178
154.117	0.130581	2.494818	97.70	89.78	0.052873
156.267	0.132050	2.494818	97.70	87.82	0.053563
158.400	0.133727	2.494818	97.70	89.04	0.054240
160.550	0.135180	2.494818	97.70	88.77	0.054913
162.683	0.137242	2.494818	97.70	88.28	0.055572
164.817	0.138715	2.494818	97.70	88.86	0.056221
166.967	0.140692	2.494818	97.70	89.14	0.056866
169.100	0.142299	2.494818	97.70	89.75	0.057497
171.250	0.143960	2.494818	97.70	89.38	0.058124
173.383	0.145503	2.494818	97.70	89.97	0.058738
175.533	0.146776	2.494818	97.70	90.18	0.059348
177.667	0.148873	2.494818	97.70	91.73	0.059945
179.800	0.150139	2.494818	97.70	91.49	0.060533
181.950	0.151782	2.494818	97.70	91.59	0.061118
184.083	0.153003	2.494818	97.70	90.60	0.061691
186.233	0.154432	2.494818	97.70	88.90	0.062261
188.367	0.156017	2.494818	97.70	92.18	0.062819
190.517	0.157841	2.494818	97.70	91.85	0.063372
192.650	0.158346	2.494818	97.70	91.52	0.063914
194.783	0.159595	2.494818	97.70	91.63	0.064452
196.933	0.160933	2.494818	97.70	92.32	0.064987
199.067	0.161709	2.494818	97.70	91.77	0.065512
201.217	0.163275	2.494818	97.70	90.45	0.066035
203.350	0.164548	2.494818	97.70	91.40	0.066546
205.500	0.165805	2.494818	97.70	92.69	0.067055
207.633	0.167151	2.494818	97.70	92.21	0.067553
209.767	0.168543	2.494818	97.70	94.86	0.068043
211.917	0.169119	2.494818	97.70	92.14	0.068532
214.050	0.170561	2.494818	97.70	92.66	0.069012
216.200	0.171992	2.494818	97.70	92.95	0.069488
218.333	0.172892	2.494818	97.70	91.48	0.069954
220.483	0.174221	2.494818	97.70	90.53	0.070418
222.617	0.175369	2.494818	97.70	90.03	0.070871
224.750	0.176829	2.494818	97.70	95.01	0.071318
226.900	0.177750	2.494818	97.70	91.04	0.071761
229.033	0.178858	2.494818	97.70	92.96	0.072196
231.183	0.179752	2.494818	97.70	94.42	0.072629
233.317	0.181182	2.494818	97.70	94.27	0.073052

235.467	0.181935	2.494818	97.70	93.55	0.073473
237.600	0.183135	2.494818	97.70	91.68	0.073885
239.750	0.184299	2.494818	97.70	93.24	0.074294
241.883	0.185060	2.494818	97.70	91.85	0.074695
244.017	0.186140	2.494818	97.70	92.90	0.075091
246.167	0.187256	2.494818	97.70	93.32	0.075484
248.300	0.188415	2.494818	97.70	94.64	0.075868
250.450	0.188978	2.494818	97.70	92.12	0.076251
252.583	0.189702	2.494818	97.70	91.99	0.076627
254.717	0.190784	2.494818	97.70	93.45	0.076998
256.867	0.191679	2.494818	97.70	94.20	0.077367
259.000	0.192614	2.494818	97.70	93.05	0.077728
261.150	0.193541	2.494818	97.70	94.02	0.078087
263.283	0.194629	2.494818	97.70	95.99	0.078438
265.433	0.195387	2.494818	97.70	95.33	0.078787
267.600	0.196083	2.494818	97.70	95.23	0.079134
269.733	0.196910	2.494818	97.70	94.92	0.079472
271.883	0.197671	2.494818	97.70	95.20	0.079808
274.017	0.198591	2.494818	97.70	94.18	0.080138
276.150	0.199222	2.494818	97.70	96.18	0.080463
278.300	0.200447	2.494818	97.70	93.54	0.080786
280.433	0.200859	2.494818	97.70	94.89	0.081101
282.583	0.201801	2.494818	97.70	95.70	0.081416
284.717	0.202213	2.494818	97.70	93.67	0.081725
286.867	0.203172	2.494818	97.70	92.59	0.082032
289.000	0.203882	2.494818	97.70	94.37	0.082333
291.150	0.204548	2.494818	97.70	96.60	0.082632
293.283	0.204977	2.494818	97.70	94.68	0.082926
295.433	0.205658	2.494818	97.70	94.32	0.083219
297.567	0.206498	2.494818	97.70	95.83	0.083506
299.717	0.207362	2.494818	97.70	97.30	0.083791
301.850	0.208179	2.494818	97.70	97.40	0.084069
304.000	0.208613	2.494818	97.70	96.48	0.084346
306.133	0.209628	2.494818	97.70	95.89	0.084616
308.283	0.209960	2.494818	97.70	97.40	0.084886
310.417	0.210577	2.494818	97.70	94.39	0.085150
312.550	0.211308	2.494818	97.70	96.05	0.085412
314.700	0.211829	2.494818	97.70	96.42	0.085671
316.833	0.212710	2.494818	97.70	95.79	0.085925
318.983	0.213131	2.494818	97.70	95.16	0.086178

321.117	0.213754	2.494818	97.70	95.67	0.086426
323.250	0.214297	2.494818	97.70	95.82	0.086670
325.400	0.214922	2.494818	97.70	97.12	0.086914
327.533	0.215684	2.494818	97.70	95.27	0.087152
329.683	0.216625	2.494818	97.70	96.80	0.087387
331.817	0.216868	2.494818	97.70	96.82	0.087617
333.967	0.217630	2.494818	97.70	95.84	0.087846
336.100	0.218103	2.494818	97.70	95.16	0.088071
338.233	0.218636	2.494818	97.70	96.22	0.088292
340.383	0.219466	2.494818	97.70	95.05	0.088512
342.517	0.219929	2.494818	97.70	97.59	0.088726
344.667	0.220535	2.494818	97.70	96.51	0.088940
346.800	0.220942	2.494818	97.70	94.30	0.089149
348.933	0.221967	2.494818	97.70	96.65	0.089354
351.083	0.222135	2.494818	97.70	96.22	0.089557
353.217	0.223008	2.494818	97.70	95.30	0.089757
355.367	0.223170	2.494818	97.70	95.67	0.089955
357.500	0.223722	2.494818	97.70	97.02	0.090149
359.650	0.223767	2.494818	97.70	98.41	0.090344
361.783	0.224226	2.494818	97.70	95.33	0.090535
363.917	0.224596	2.494818	97.70	96.11	0.090725
366.067	0.224852	2.494818	97.70	99.47	0.090914
368.200	0.225271	2.494818	97.70	98.88	0.091100
370.350	0.225361	2.494818	97.70	100.00	0.091286
372.483	0.226064	2.494818	97.70	96.68	0.091468
374.633	0.226124	2.494818	97.70	93.10	0.091650
376.767	0.226746	2.494818	97.70	97.78	0.091829
378.917	0.227128	2.494818	97.70	95.63	0.092006
381.050	0.227325	2.494818	97.70	96.33	0.092181
383.200	0.227922	2.494818	97.70	97.65	0.092354
385.333	0.228451	2.494818	97.70	98.76	0.092524
387.467	0.228700	2.494818	97.70	97.23	0.092691
389.617	0.229053	2.494818	97.70	95.35	0.092858
391.750	0.229429	2.494818	97.70	97.75	0.093021
393.900	0.229940	2.494818	97.70	94.52	0.093184
396.033	0.230397	2.494818	97.70	97.07	0.093343
398.183	0.230478	2.494818	97.70	95.55	0.093501
400.317	0.230754	2.494818	97.70	97.72	0.093658
402.450	0.230974	2.494818	97.70	98.00	0.093813
404.600	0.231158	2.494818	97.70	95.58	0.093968

406.733	0.231378	2.494818	97.70	98.47	0.094121
408.883	0.231777	2.494818	97.70	98.83	0.094273
411.033	0.232300	2.494818	97.70	98.01	0.094423
413.167	0.232485	2.494818	97.70	98.49	0.094570
415.300	0.232825	2.494818	97.70	97.36	0.094716
417.450	0.233121	2.494818	97.70	96.86	0.094861
419.583	0.233611	2.494818	97.70	98.56	0.095003
421.733	0.233848	2.494818	97.70	98.42	0.095144
423.867	0.233809	2.494818	97.70	99.29	0.095283
426.000	0.233458	2.494818	97.70	99.59	0.095423
428.150	0.233575	2.494818	97.70	97.70	0.095565

3. Aminocatalytic Michael reaction

3.1. Experimental setup

The Michael reaction was setup at room temperature in standard NMR tubes from stock solutions prior to data acquisition within the NMR spectrometer. To a sample of freshly vacuum distilled propanal (**3**) (2.40 M) and *trans-β*-nitrostyrene (**4**) (2.40 M) in toluene-d8 (total volume 0.50 mL), prepared from an accurate stock solution, was added a pyrazine/toluene-d8 reference capillary for pre-shimming to enable high quality data collection. The capillary was then retrieved before addition of 0.10 mL of a stock solution of proline-derived catalyst **5** ((*S*)-(–)-*α*,*α*-diphenyl-2-pyrrolidinemethanol trimethylsilyl ether, 0.06 M) and acetic acid (0.60 M) in toluene-d8.

Initial reaction concentrations: **[3]$_o$** = **[4]$_o$ =** 2.00 M, **[5]$_o$** = 0.01 M (0.5 mol%), **[AcOH]$_o$** = 0.10 M (5.0 mol%). The sample tube was then shaken thoroughly and the reference capillary returned to the tube before loading into the NMR spectrometer and the kinetic data collected using an automated shim-acquisition sequence. The timing between the catalyst addition and the start of the acquisition sequence was noted for use in the kinetic calculations. Spectra were collated and product **6** and catalyst concentrations measured by peak integration within the limits of the data (see NMR spectra below).

3.2. NMR spectra

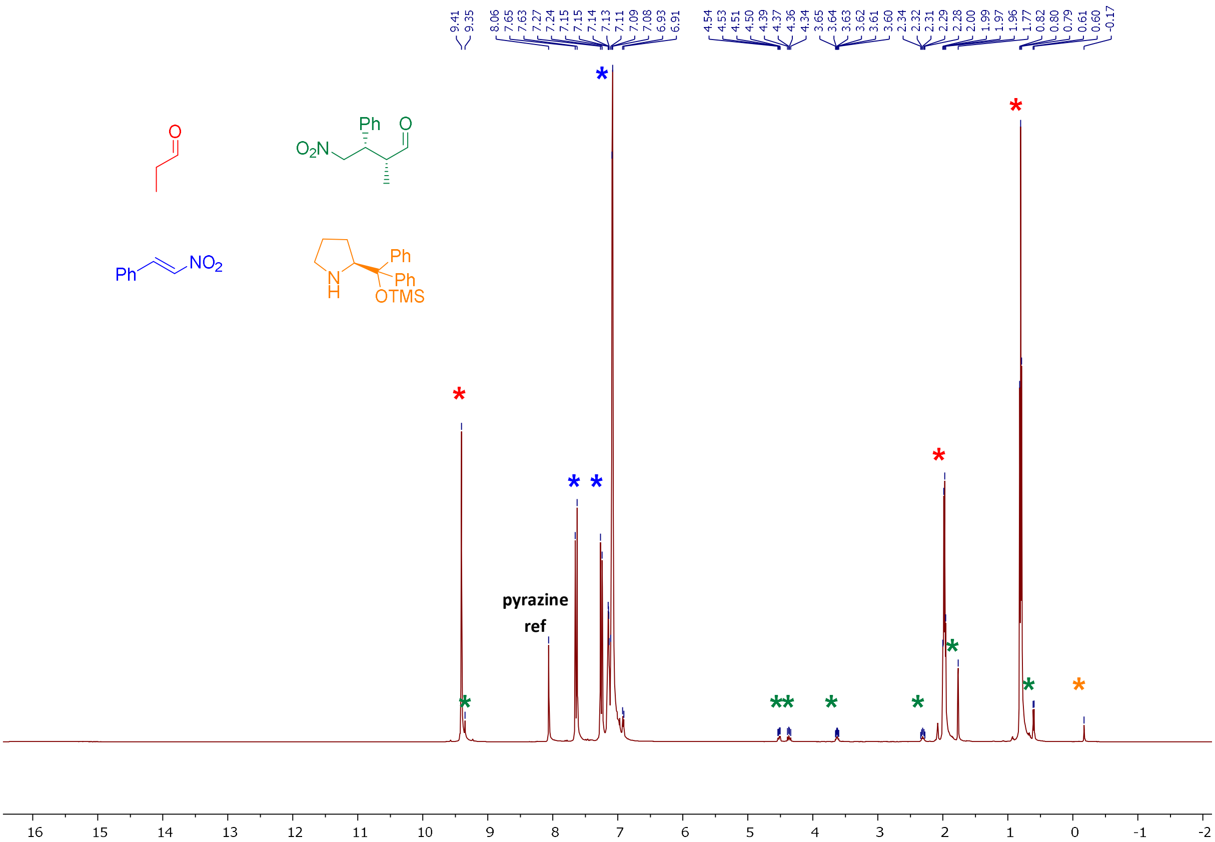

Figure S11. ^{1}H NMR spectrum (500 MHz, toluene-d8) of the Michael reaction after 8 minutes.

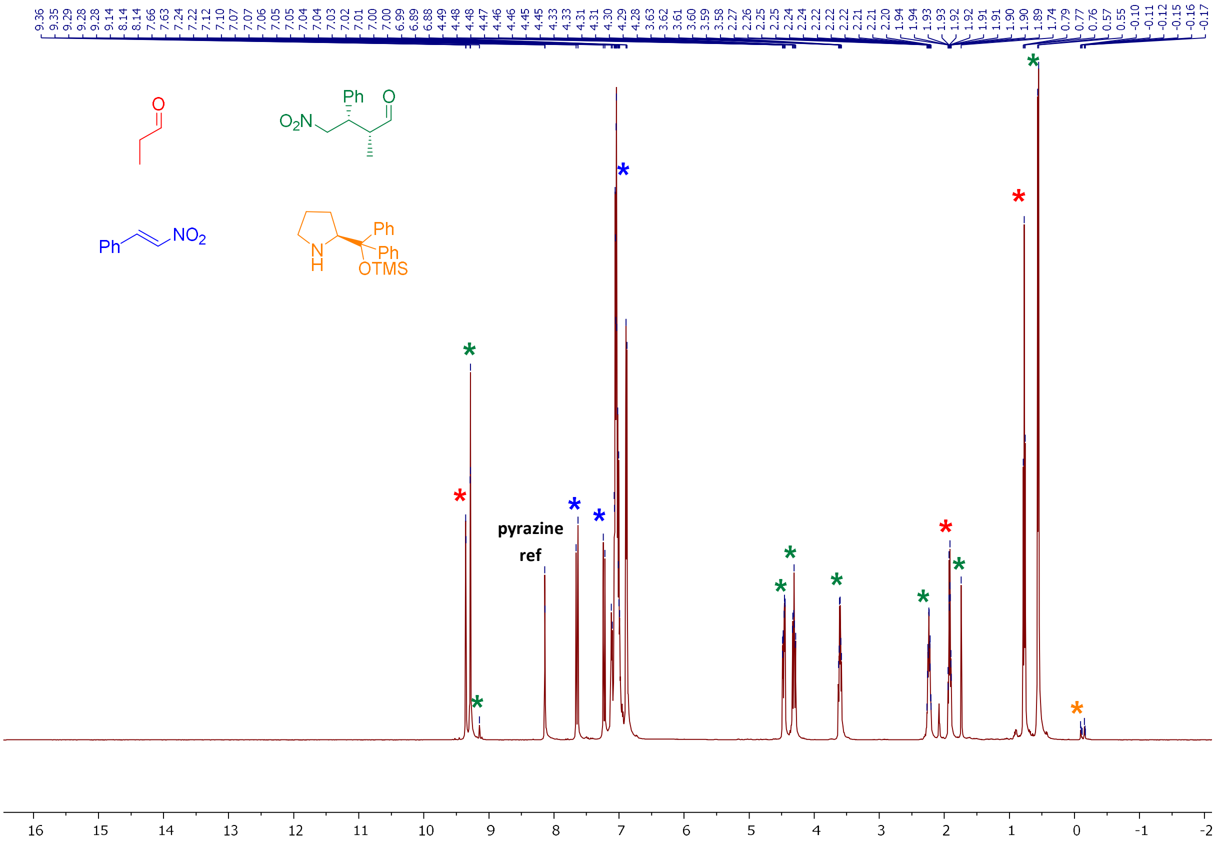

Figure S12. ^{1}H NMR spectrum (500 MHz, toluene-d8) of the Michael reaction after 198 mins.

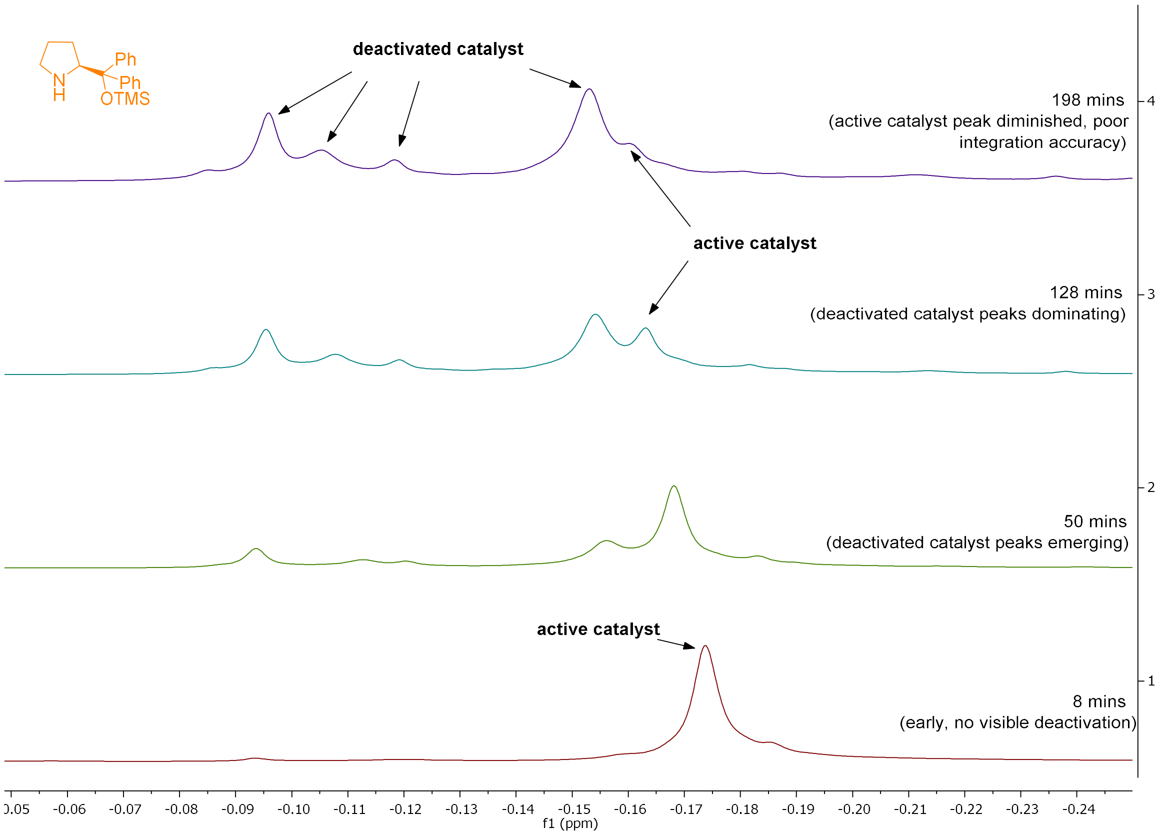

Figure S13. Stacked ^{1}H NMR spectra (500 MHz, toluene-d8) of the Michael reaction showing loss of active catalyst.

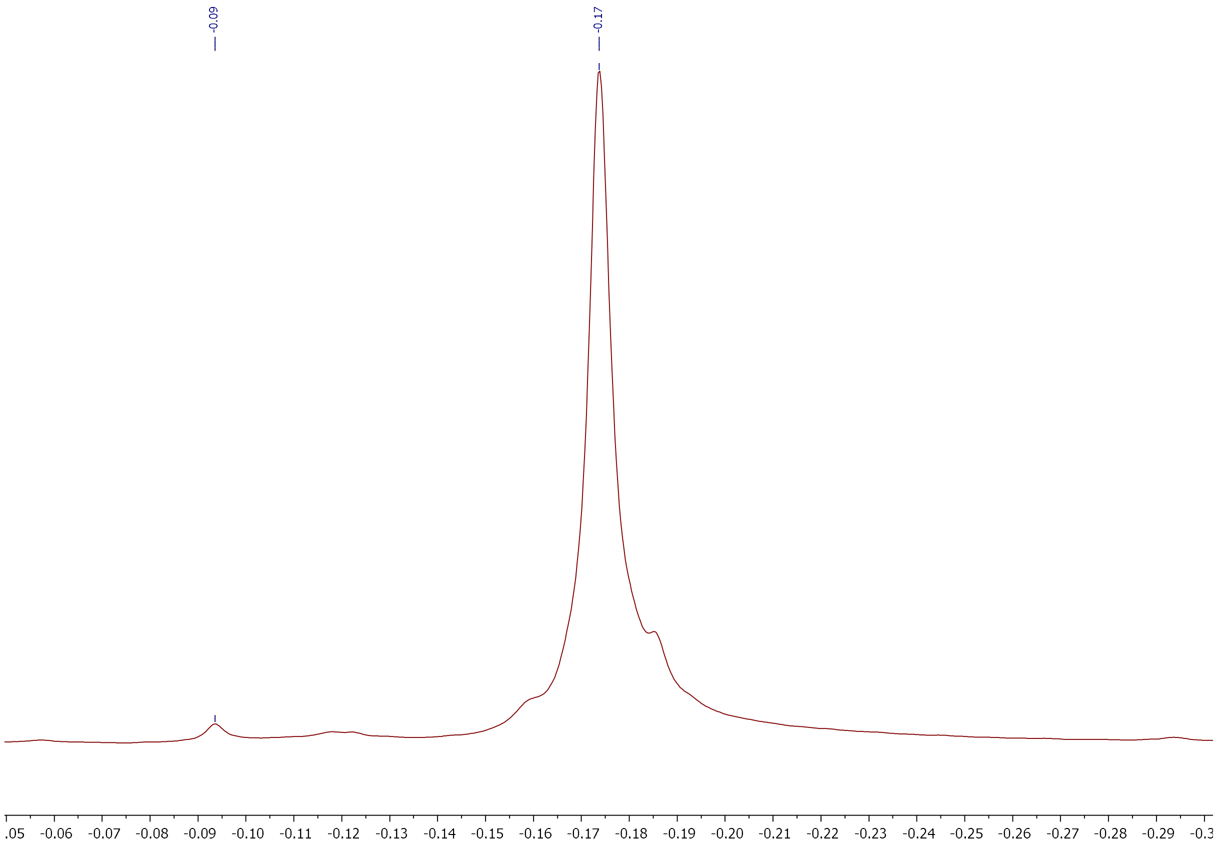

Figure S14. ^{1}H NMR spectrum (500 MHz, toluene-d8) of the catalyst $Si(CH_3)_3$ peak (8 mins).

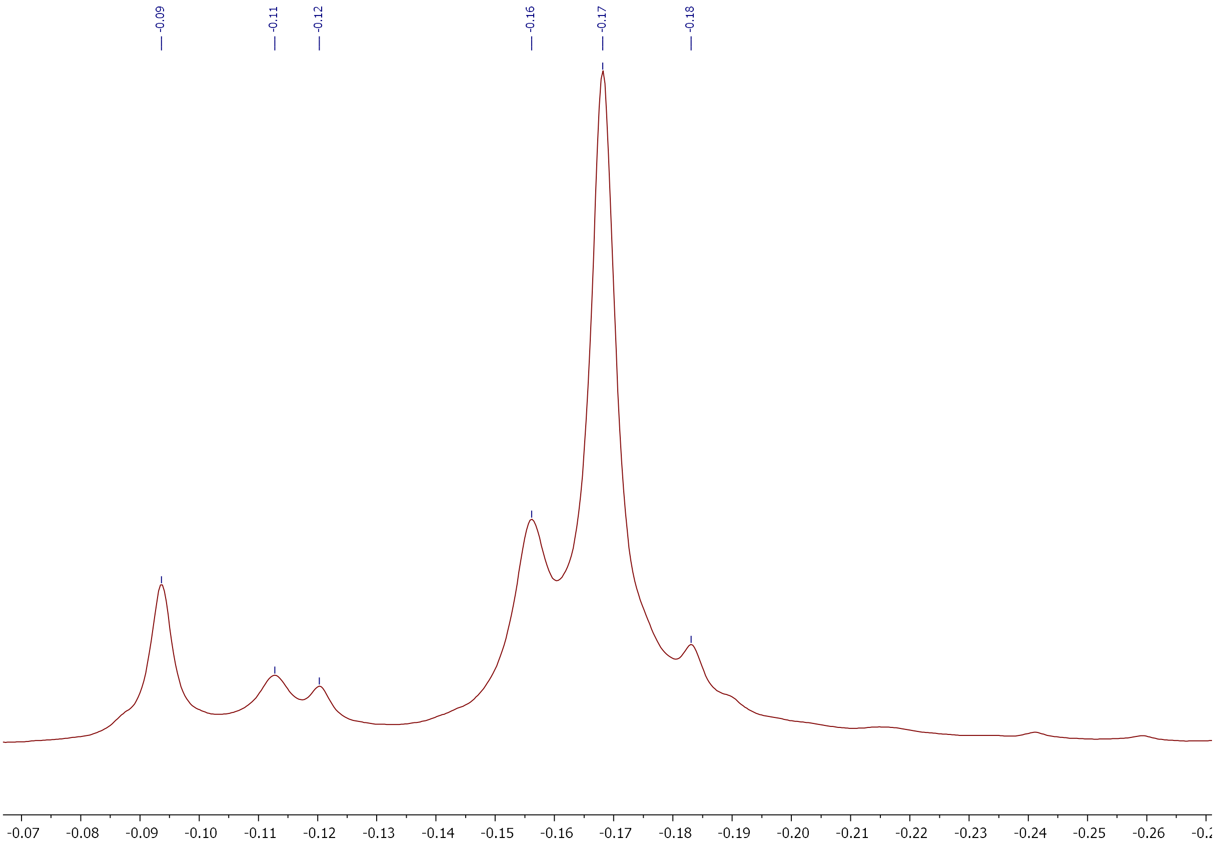

Figure S15. ^{1}H NMR spectrum (500 MHz, toluene-d8) of the catalyst $Si(CH_3)_3$ peak (50 mins).

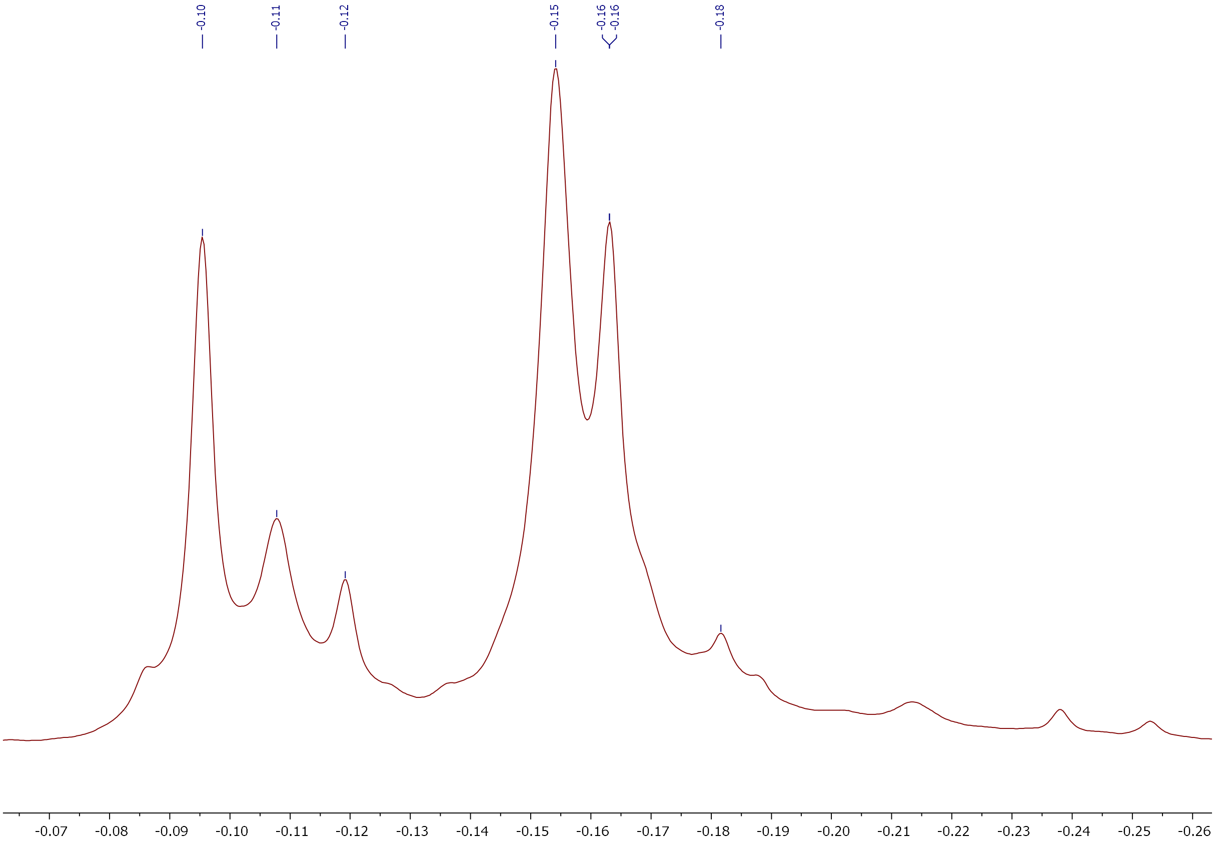

Figure S16. ^{1}H NMR spectrum (500 MHz, toluene-d8) of the catalyst $Si(CH_3)_3$ peak (128 mins).

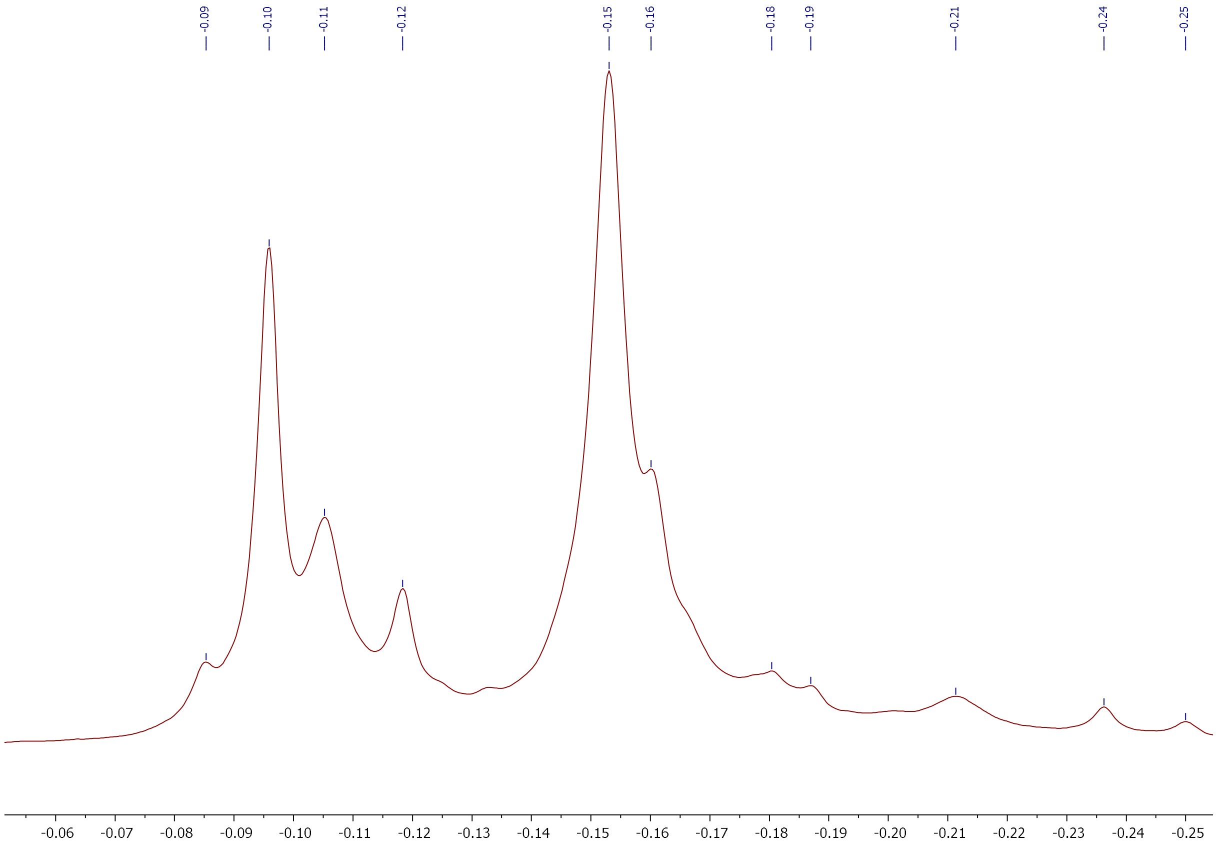

Figure S17. ^{1}H NMR spectrum (500 MHz, toluene-d8) of the catalyst $Si(CH_3)_3$ peak (198 mins).

3.3. Kinetic data (Figure 3 and 5)

Figures 3a and 3b of the manuscript show respectively the concentration of product and catalyst obtained from the integration of consecutive ^{1}H-NMR spectra. The numerical values for both figures are shown in Table S3.

Figure 3c shows the reaction profile corrected by the amount of active catalyst measured at each time point. The time values shown in Table S3 have been calculated using the VTNA formula:

$$\textit{normalized time} = \sum_{i=1}^{n} \left(\frac{[\text{active catalyst}]_i + [\text{active catalyst}]_{i-1}}{2} \right)^1 (t_i - t_{i-1})$$

Table S3. Data for Figure 3.

time / min	[P] / M	[active catalyst] / mM	VTNA
8.570	0.0858	7.1692	0.0614
14.480	0.1637	7.3482	0.1043
32.670	0.3733	6.0642	0.2263
38.580	0.4341	5.5163	0.2605
50.430	0.5486	5.0053	0.3229
56.350	0.6065	4.5086	0.3510
62.270	0.6567	4.3886	0.3774

68.180	0.7041	4.0947	0.4025
74.120	0.7542	3.9517	0.4263
80.050	0.7985	3.7453	0.4492
85.980	0.8385	3.6853	0.4712
91.900	0.8765	3.4993	0.4925
97.830	0.9121	3.3003	0.5126
103.770	0.9489	2.8859	0.5310
109.700	0.9820	2.7834	0.5478
115.630	1.0130	2.6001	0.5638
121.570	1.0400	2.4126	0.5787
127.500	1.0671	2.4084	0.5930
133.430	1.0938	2.4142	0.6073
139.370	1.1157	-	-
145.280	1.1377	-	-
151.200	1.1594	-	-
157.120	1.1767	-	-
163.050	1.1938	-	-
168.980	1.2112	-	-
174.900	1.2255	-	-
180.830	1.2397	-	-
186.750	1.2538	-	-
192.670	1.2628	-	-
198.600	1.2760	-	-
204.530	1.2829	-	-
210.470	1.2934	-	-
216.400	1.3059	-	-
222.320	1.3138	-	-
228.250	1.3258	-	-
234.180	1.3306	-	-
240.120	1.3396	-	-
246.050	1.3446	-	-
251.980	1.3528	-	-
257.920	1.3596	-	-
263.830	1.3632	-	-
269.770	1.3701	-	-
275.700	1.3737	-	-
281.630	1.3778	-	-
287.550	1.3850	-	-
293.480	1.3893	-	-
299.400	1.3943	-	-

305.320	1.3981	-	-
311.250	1.4023	-	-

Figure 5a shows the original temporal profile of the concentration of product obtained from the integration of consecutive ^{1}H-NMR spectra and the corresponding data is shown in Table S4.

Figure 5b shows the linearized VTNA profile resulting from the normalization of the time scale by the concentration of estimated active catalyst. All the corresponding numerical data is shown in Table S4.

Figure 5c shows the comparison of the estimated temporal profile of percentage of catalyst and the original concentration measured from the ^{1}H-NMR spectra. The values for the estimated concentration profile were found using Solver. In this case, we imposed the condition that the concentration of catalyst could not increase during the reaction. To do so in Excel, the concentration of catalyst at a given time was defined as the concentration in the previous time minus a given increment. Specifically, these increment values represented the 'Variable Cells' changed by Solver in order to maximize the R^2 (SQR function: square of the Pearson product moment correlation coefficient through data points (concentration of product, normalized time)) of the resulting VTNA profile shown in Figure 5c.

Table S4. Data for Figure 5.

time / min	[P] / M	estimated [active cat] / mM	% estimated active catalyst	% measured active catalyst	VTNA using estimated [active catalyst]
8.570	0.0858	7.1692	71.69	71.69	0.0614
14.480	0.1637	6.4756	64.76	73.48	0.1018
32.670	0.3733	5.5021	55.02	60.64	0.2107
38.580	0.4341	5.0648	50.65	55.16	0.2419
50.430	0.5486	5.0648	50.65	50.05	0.3019
56.350	0.6065	4.7706	47.71	45.09	0.3311
62.270	0.6567	4.2293	42.29	43.89	0.3577
68.180	0.7041	4.2293	42.29	40.95	0.3827
74.120	0.7542	4.2162	42.16	39.52	0.4078
80.050	0.7985	3.6969	36.97	37.45	0.4312
85.980	0.8385	3.3459	33.46	36.85	0.4521
91.900	0.8765	3.1862	31.86	34.99	0.4715
97.830	0.9121	3.1862	31.86	33.00	0.4903
103.770	0.9489	3.1094	31.09	28.86	0.5090
109.700	0.9820	2.8056	28.06	27.83	0.5266
115.630	1.0130	2.4796	24.80	26.00	0.5423
121.570	1.0400	2.3702	23.70	24.13	0.5567
127.500	1.0671	2.3702	23.70	24.08	0.5707
133.430	1.0938	2.1213	21.21	24.14	0.5840
139.370	1.1157	1.9158	19.16	-	0.5960

145.280	1.1377	1.9158	19.16	-	0.6073
151.200	1.1594	1.7161	17.16	-	0.6181
157.120	1.1767	1.4955	14.95	-	0.6276
163.050	1.1938	1.4955	14.95	-	0.6365
168.980	1.2112	1.3931	13.93	-	0.6450
174.900	1.2255	1.2548	12.55	-	0.6529
180.830	1.2397	1.2548	12.55	-	0.6603
186.750	1.2538	1.0063	10.06	-	0.6670
192.670	1.2628	0.8951	8.95	-	0.6726
198.600	1.2760	0.8930	8.93	-	0.6779
204.530	1.2829	0.8930	8.93	-	0.6832
210.470	1.2934	0.8930	8.93	-	0.6885
216.400	1.3059	0.8930	8.93	-	0.6938
222.320	1.3138	0.8930	8.93	-	0.6991
228.250	1.3258	0.7262	7.26	-	0.7039
234.180	1.3306	0.6094	6.09	-	0.7079
240.120	1.3396	0.6094	6.09	-	0.7115
246.050	1.3446	0.6094	6.09	-	0.7151
251.980	1.3528	0.6094	6.09	-	0.7187
257.920	1.3596	0.4611	4.61	-	0.7219
263.830	1.3632	0.4434	4.43	-	0.7246
269.770	1.3701	0.4434	4.43	-	0.7272
275.700	1.3737	0.4434	4.43	-	0.7298
281.630	1.3778	0.4434	4.43	-	0.7325
287.550	1.3850	0.4434	4.43	-	0.7351
293.480	1.3893	0.4434	4.43	-	0.7377
299.400	1.3943	0.3922	3.92	-	0.7402
305.320	1.3981	0.3532	3.53	-	0.7424
311.250	1.4023	0.3359	3.36	-	0.7444

3.4. *Study of catalyst deactivation pathways*

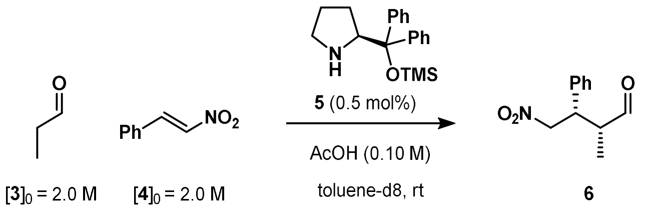

Michael addition of propanal (**3**) to *trans-β*-nitrostyrene (**4**) is generally performed with 10-20 mol% catalyst loading due to catalyst deactivation processes. When the reaction was run at 0.5 mol% of catalyst **5**, the reaction failed to reach completion, which could be due to catalyst deactivation.

3.4.1. *Proof of catalyst deactivation*

In order to prove that catalyst deactivation was the reason for the uncompleted reaction, we performed three different experiments: same excess, same excess with product added and extra addition of fresh catalyst.

The **same excess** reaction ([**3**]$_0$ = [**4**]$_0$ = 0.6 M) is quicker than the **standard reaction** ([**3**]$_0$ = [**4**]$_0$ = 2.0 M) at the concentration of [**3**] = 0.6 M. This difference in reactivity could be due to product inhibition or catalyst deactivation. To discern between these two possibilities we ran a **same excess reaction with product added** ([**3**]$_0$ = [**4**]$_0$ = 0.6 M, [**6**] = 1.135 M). The **same excess reaction with product added** is not just quicker than the standard reaction when [**3**] = 0.6 M, but it is also quicker than the **same excess** reaction. This proves that the reaction, rather than suffering product inhibition, is accelerated by the presence of product. Therefore, the reason why the **standard reaction** at [**3**] = 0.6 M was slower than the **same excess** reaction should be catalyst deactivation.

We also confirmed catalyst deactivation by adding 0.5 mol% of fresh catalyst to the **standard reaction** once it was not progressing anymore. The reaction immediately recovered a good rate until the complete consumption of starting material (**catalyst added**).

	[**3**]$_0$	[**4**]$_0$	[**6**]$_0$
**standard reaction**	2.0 M	2.0 M	-
**same excess reaction**	0.6 M	0.6 M	-
**same excess reaction with product 6 added**	0.6 M	0.6 M	1.135 M
**catalyst added**	2.0 M	2.0 M	-

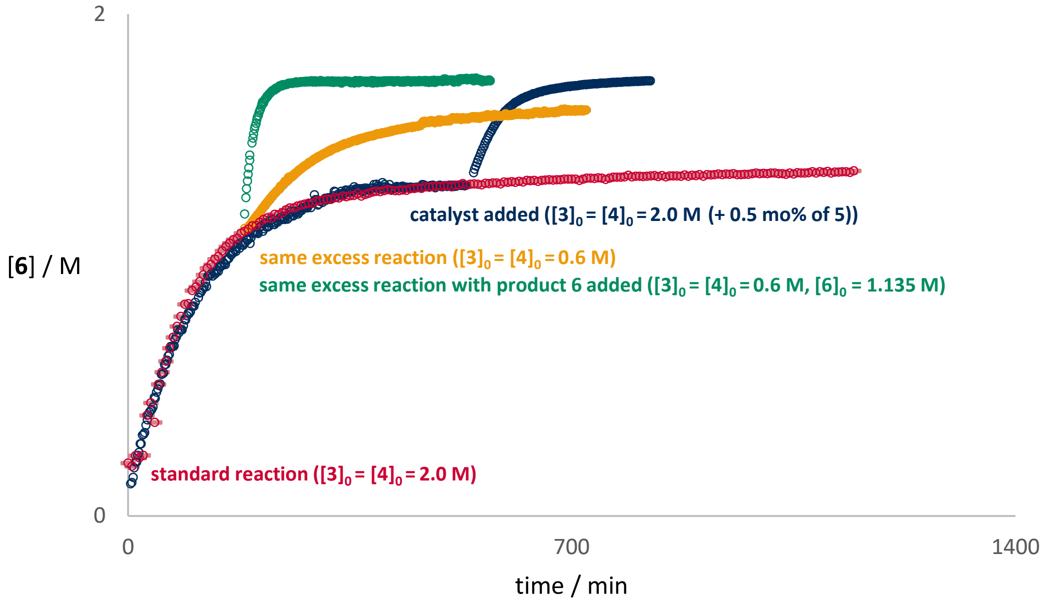

Figure S18. Kinetic profiles for the Michael reaction under different reaction conditions.

*3.4.2. Deactivation of catalyst **5** by desilylation*

A well-known mechanism of deactivation of the Jørgensen-Hayashi catalyst **5** is the cleavage of O–Si bond.[3] The desilylation of the catalyst would yield aminoalcohol **12**, which could be present at the end of the reaction as it is or as a product of reaction with propanal (**3**), *trans-β*-nitrostyrene (**4**) or product **6**.

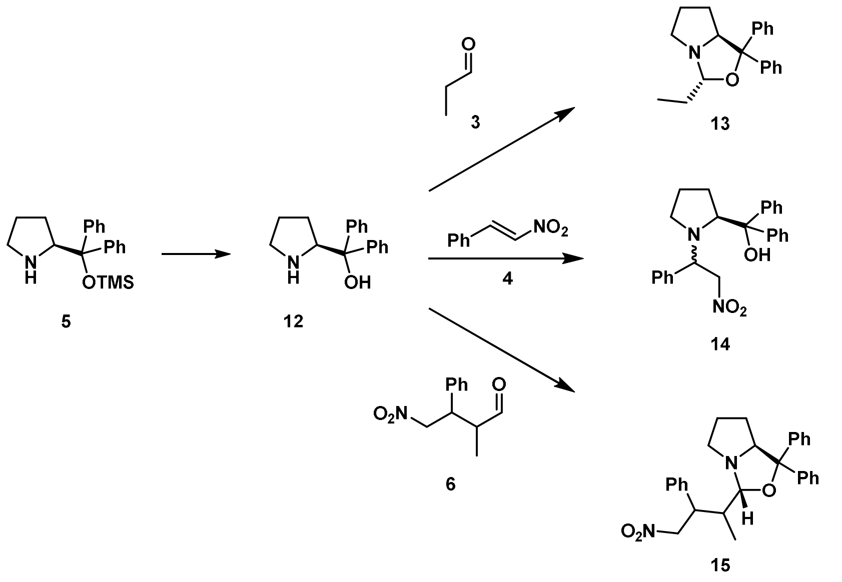

- **Free aminoalcohol 12**

The Michael reaction is run under acidic conditions ($[AcOH]_o$ = 0.1 M) and becomes more acidic over time due to the formation of product **6**. Therefore, if aminoalcohol **12** is present at the end of the reaction it could be protonated. However, the presence of aminoalcohol **12** in significant amounts in the reaction crude was discarded based on the absence of the characteristic peaks of H2 and H5 in the HSQC of the crude of the reaction (**Figure S20**).

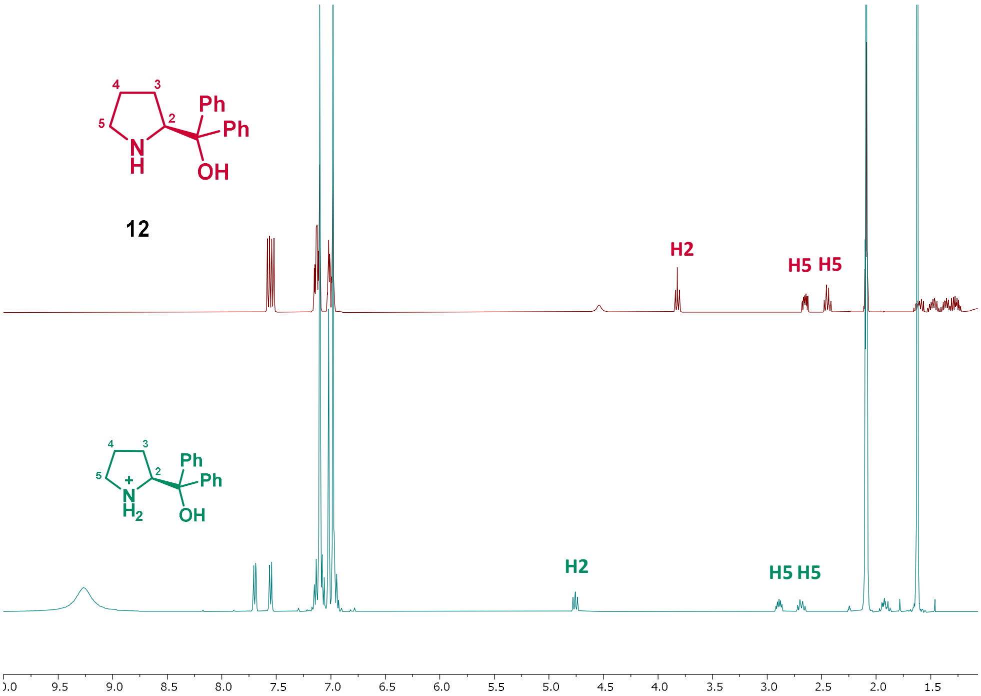

Figure S19. ^{1}H NMR spectrum (400 MHz, toluene-d8) of protonated aminoalcohol **12** compared to non-protonated aminoalcohol **12**.

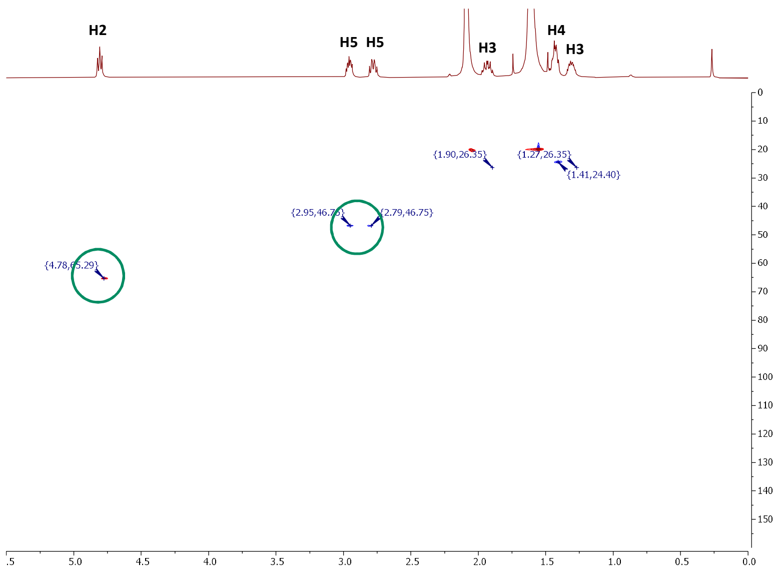

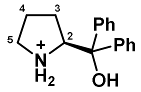

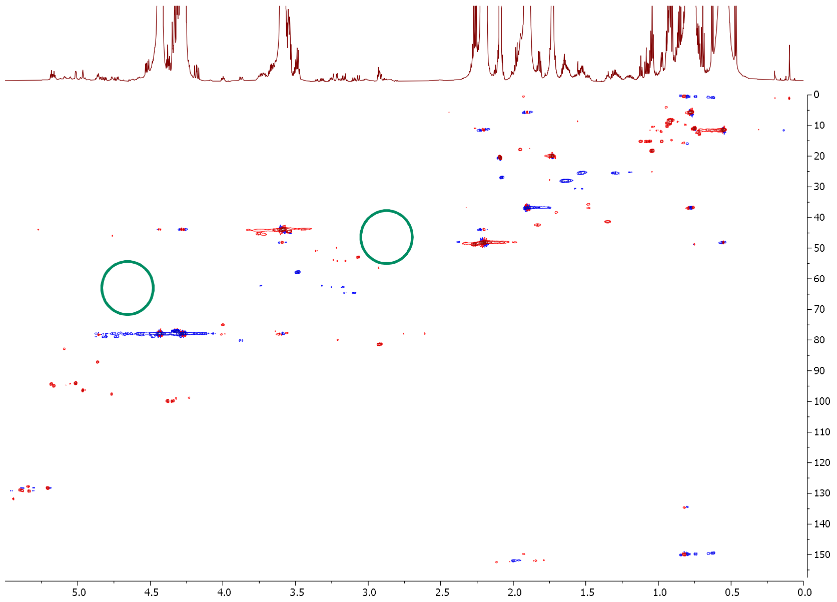

Michael reaction using standard conditions after 5 h (almost all the catalyst is deactivated).

Figure S20. HSQC spectrum (500 MHz, toluene-d8) of protonated aminoalcohol **12** and Michael reaction after 5 h.

- **Reaction of aminoalcohol 12 with propanal (3) and product 6: formation of oxazolidinones**

Aminoalcohol **12** could form oxazolidinones **13**[4] and/or **15** by reacting with propanal (**3**) or product **6**, respectively. We have characterised both oxazolidinones **13** and **15** by NMR spectroscopy. Aminoalcohol **12** (1.52 mg, 0.5 mol%) was mixed separately in two NMR tubes with propanal (**3**) (69.70 mg, 1.20 mmol) and with reaction product **6** (248.00 mg, 1.20 mmol) in toluene-d8 (0.6 mL).

The presence of significant amounts of the oxazolidinones **13** and **15** in the reaction crude after catalyst deactivation was discarded based on the comparison of NMR spectroscopic data of the oxazolidinones and the crude of the reaction. The main indicator was the absence of the characteristic H1, H2 and H5 peaks of the oxazolidinones **13** and **15** in the HSQC spectrum of the reaction mixture containing the deactivated catalyst (**Figure S21**).

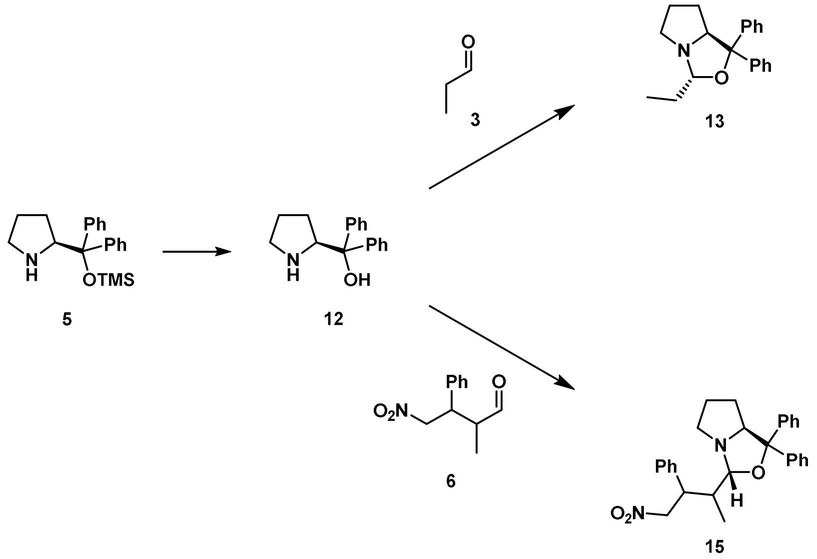

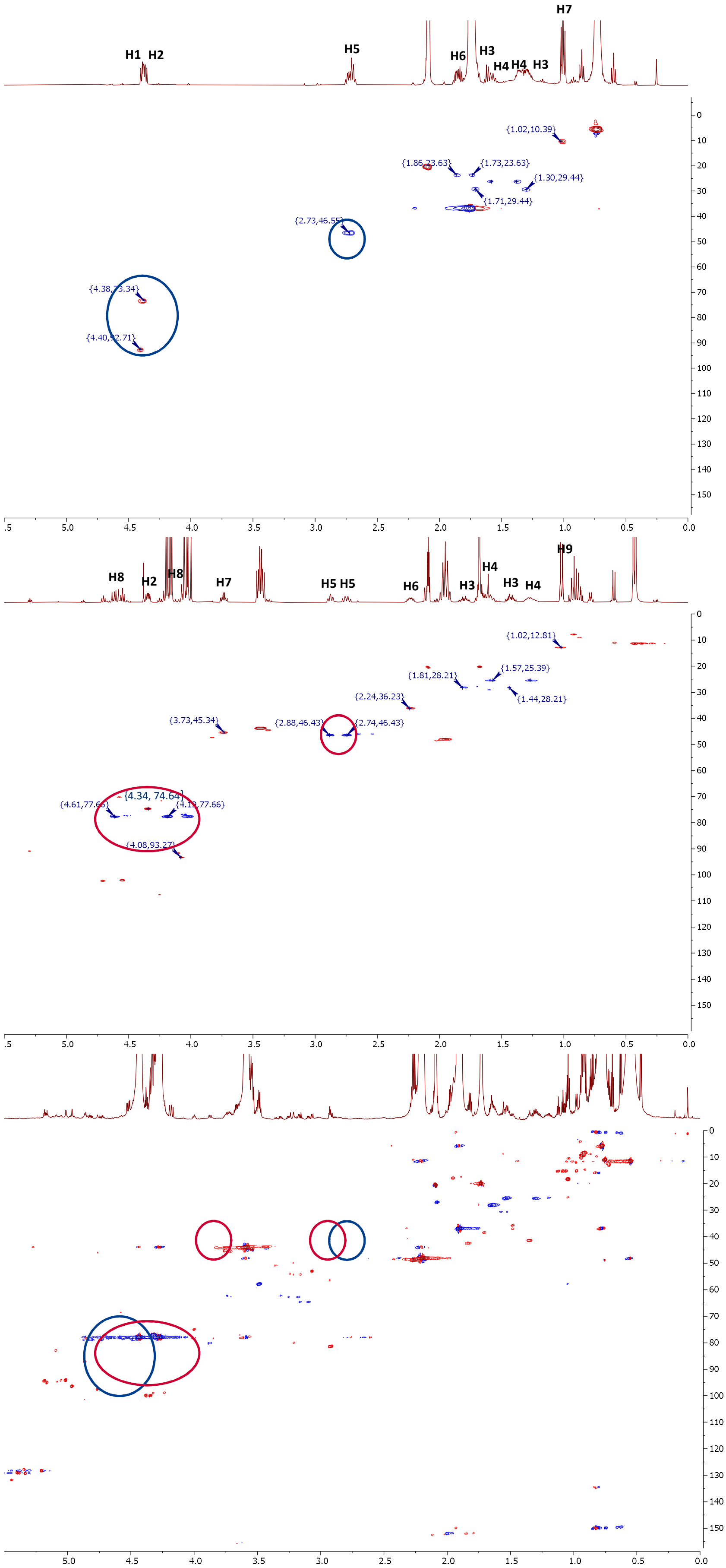

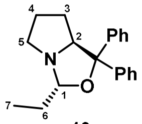

13

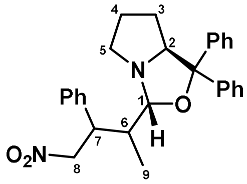

15

Michael reaction using standard conditions after 5 h (almost all the catalyst is deactivated).

Figure S21. HSQC spectrum (400 MHz, toluene-d8) of oxazolidinones **13**, **15** and crude.

- **Reaction of aminoalcohol 12 with *trans-β*-nitrostyrene (4)**

Aminoalcohol **12** could also form compound **14** by reacting with *trans-β*-nitrostyrene (**4**). Formation of **14** was only observed in the absence of AcOH.

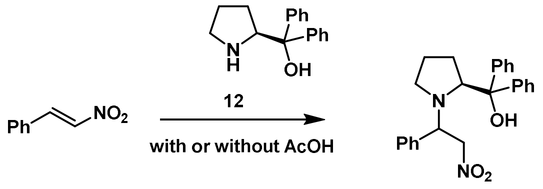

4 14

Aminoalcohol **12** (1.52 mg, 0.5 mol%) was added separately to two NMR tubes with *trans-β*-nitrostyrene (**4**) (18.00 mg, 0.12 mmol), one in the absence of AcOH and *trans-β*-nitrostyrene (**4**) (18.00 mg, 0.12 mmol), and one with AcOH (3.60 mg, 0.06 mmol) in toluene-d8 (0.6 mL).

In the presence of AcOH, only protonated aminoalcohol **12** was observed (**Figure S22**).

In the absence of AcOH, the addition product **14** was formed (**Figure S23**).

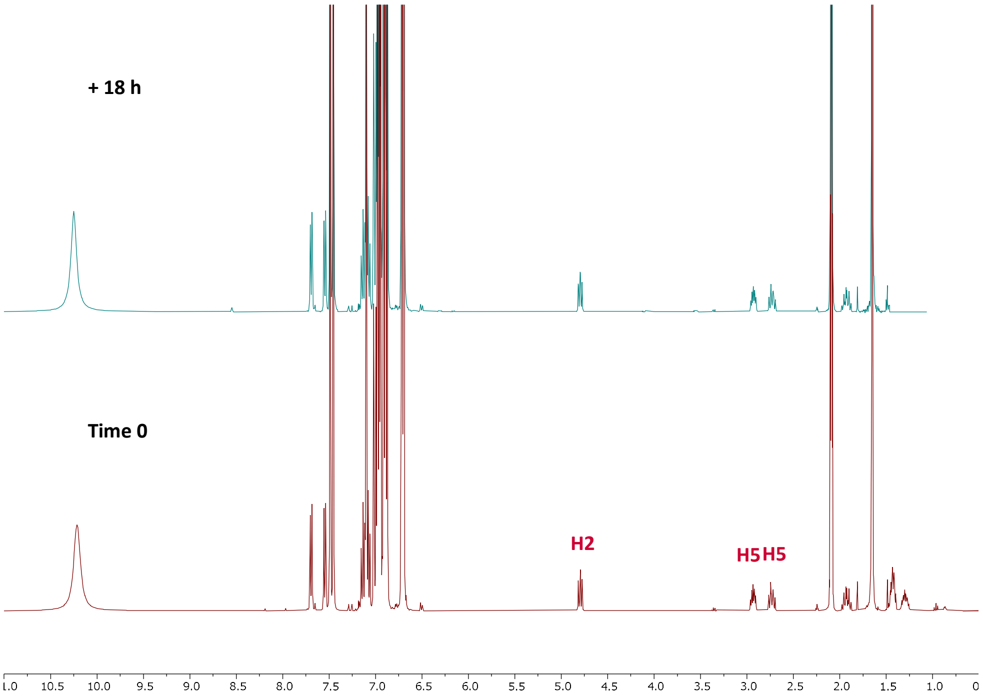

Figure S22. ^1H NMR spectrum (400 MHz, toluene-d8) of reaction of aminoalcohol **12** with *trans-β*-nitrostyrene (**4**) in the presence of AcOH immediately after mixing (time 0) and after 18 h.

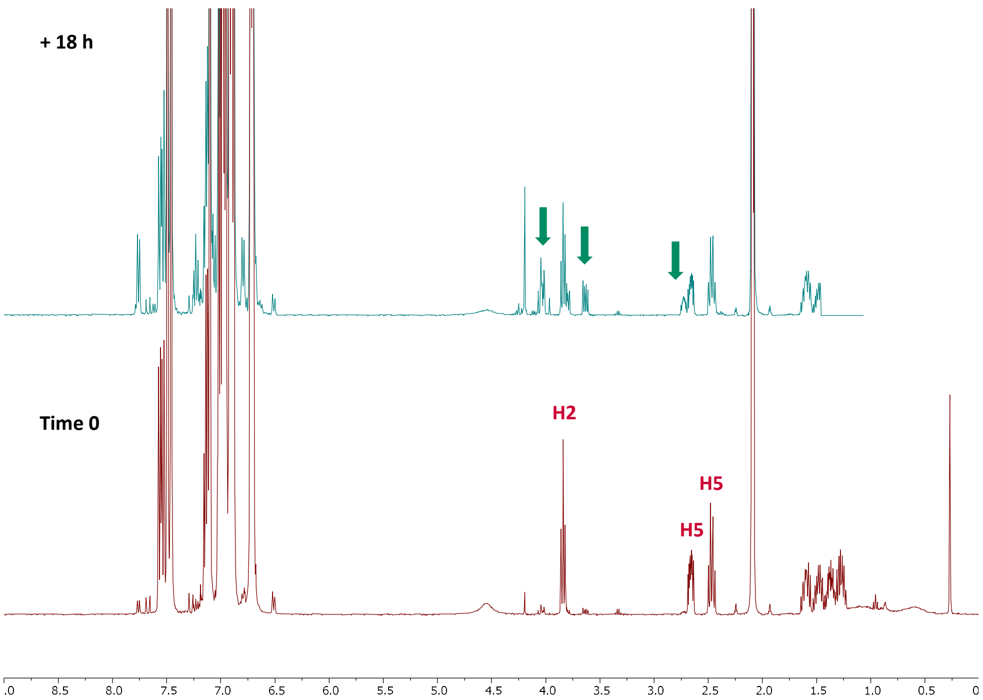

Figure S23. ^{1}H NMR spectrum (400 MHz, toluene-d8) of reaction of aminoalcohol **12** with *trans*-β-nitrostyrene (**4**) in the absence of AcOH immediately after mixing (time 0) and after 18 h.

Characterisation of products formed by reacting aminoalcohol 12 with *trans-β*-nitrostyrene (4)

Reaction of aminoalcohol **12** with *trans-β*-nitrostyrene (**4**) in toluene-d8 resulted in the formation of new signals in the ^1H NMR spectrum. The signals of the newly formed species were characterised by 2D NMR spectroscopy including COSY, HSQC and HMBC. The HMBC cross-peaks H6-C5 and H6-C2 imply that the addition to *trans-β*-nitrostyrene (**4**) proceeds through nucleophilic attack by the N atom of the aminoalcohol **12**.

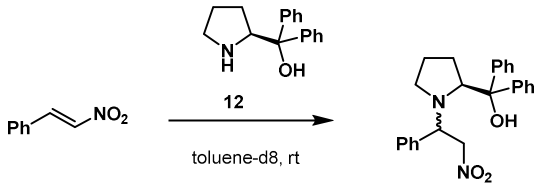

4 **14**

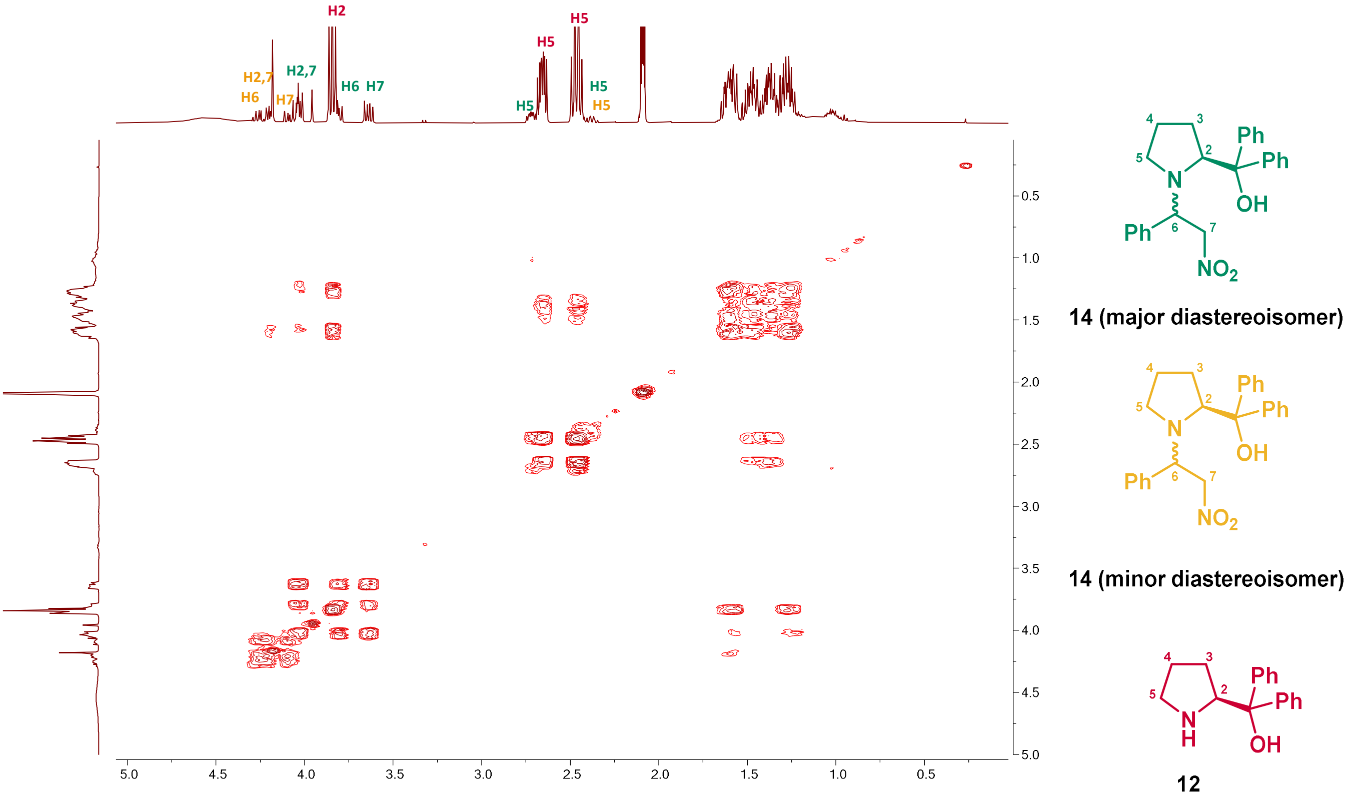

Figure S24. COSY spectrum (400 MHz, toluene-d8) used to characterise **14**.

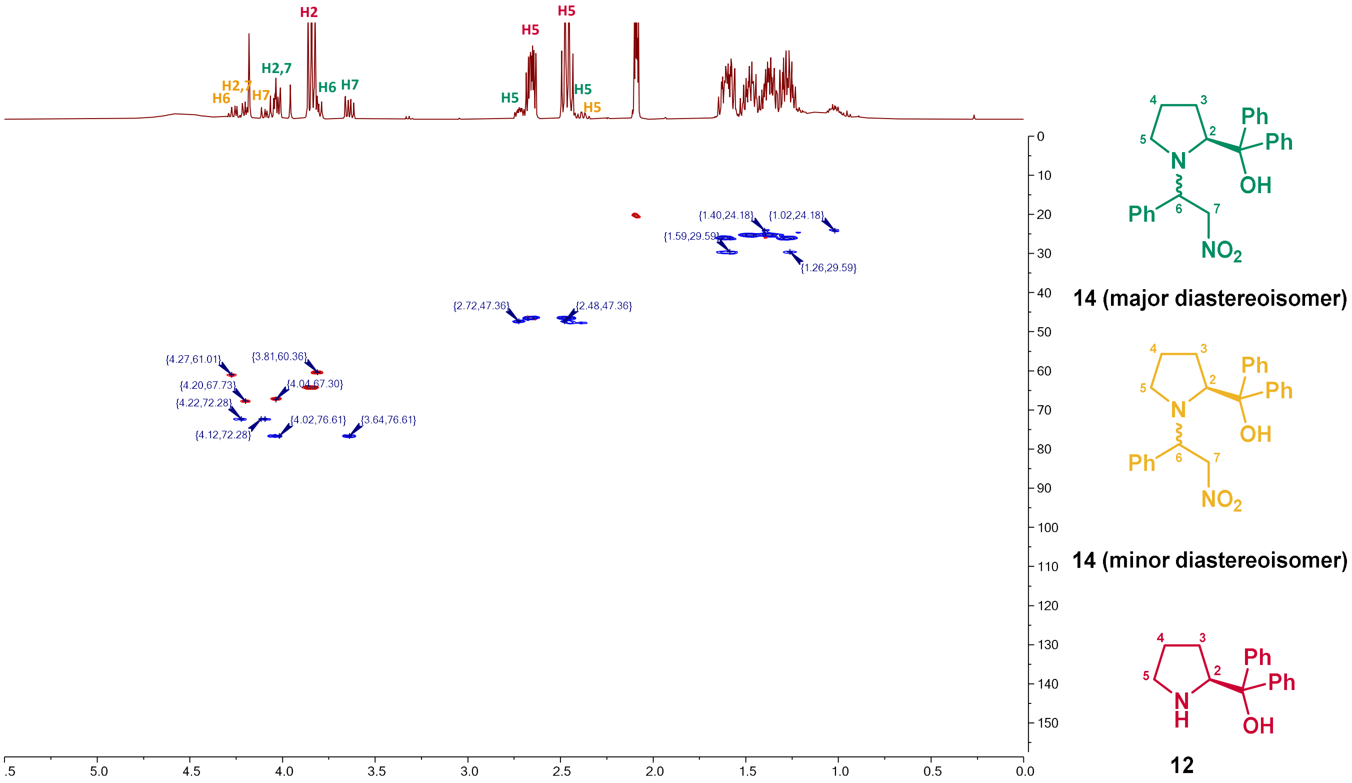

Figure S25. HSQC spectrum (400 MHz, toluene-d8) used to characterise **14**.

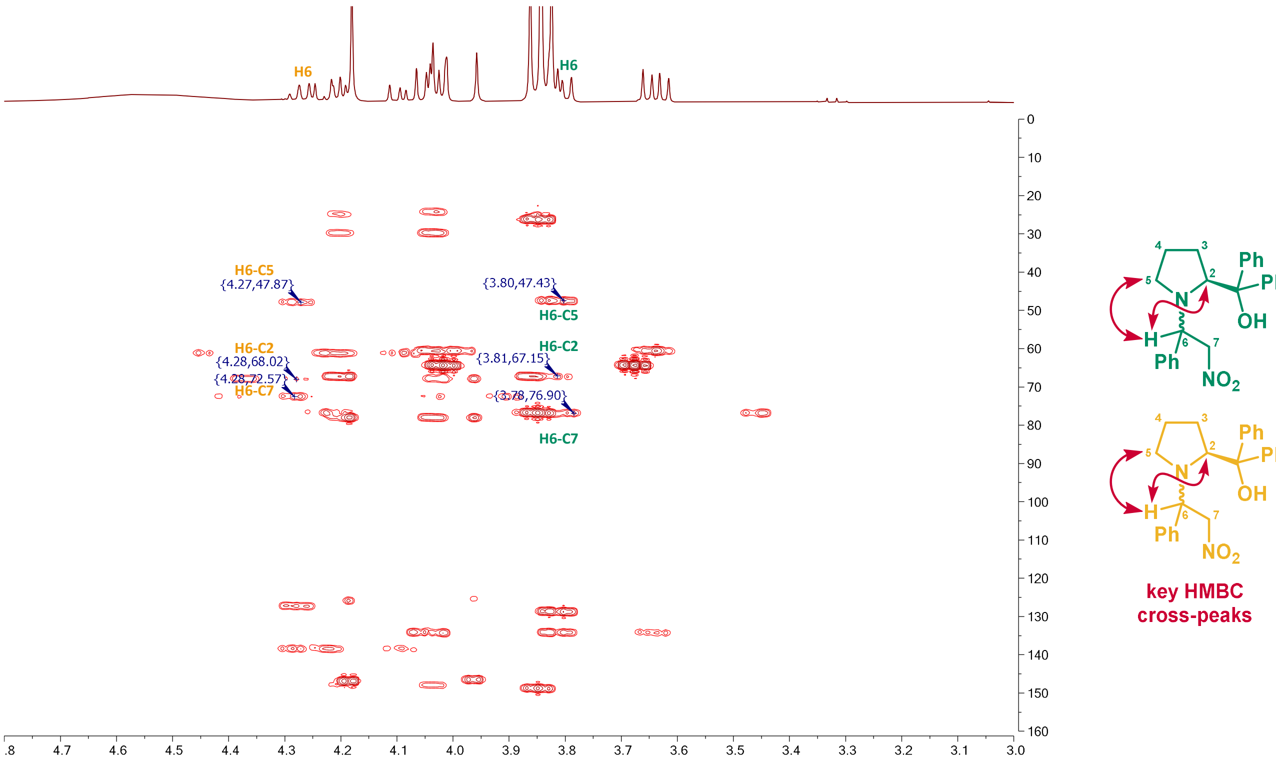

Figure S26. HSQC spectrum (400 MHz, toluene-d8) used to characterise **14**.

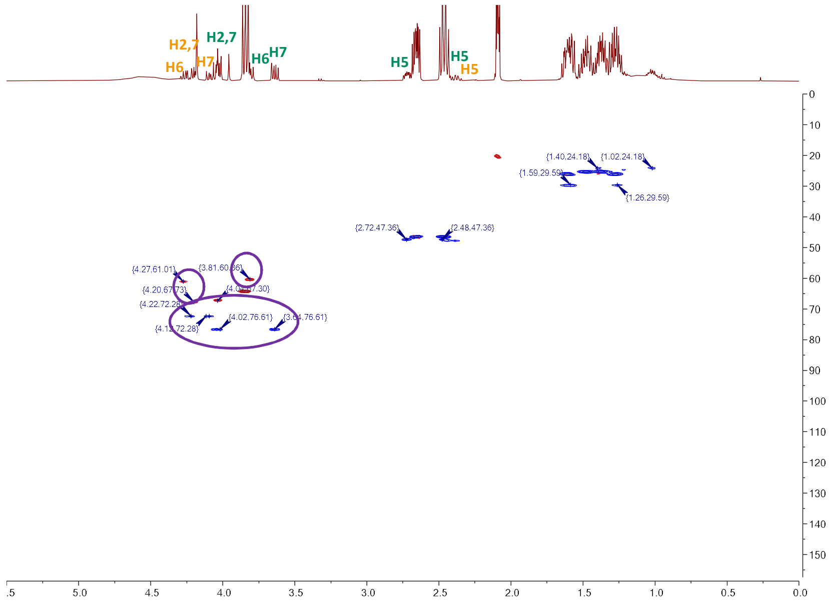

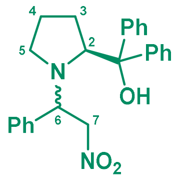

14 (major diastereoisomer)

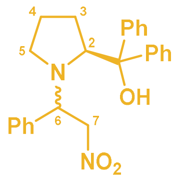

14 (minor diastereoisomer)

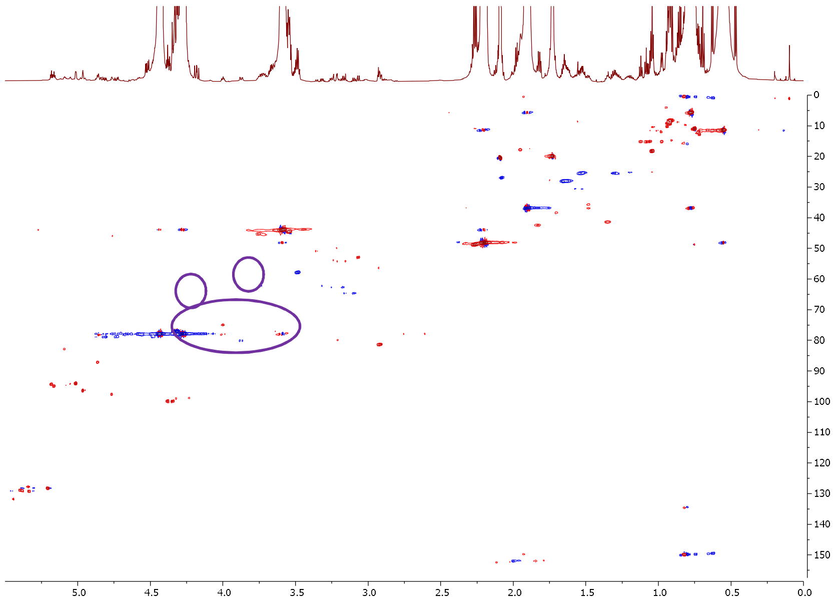

Michael reaction using standard conditions after 5 h (almost all the catalyst is deactivated).

Figure S27. Comparison of HSQC spectrum (500 MHz, toluene-d8) of deactivated catalyst **14** and the crude reaction mixture.

- **Addition of aminoalcohol 12 to the crude reaction at the end of the standard reaction**

In addition to the previous experiments, we also added extra aminoalcohol **12** to the crude reaction mixture after 5 h (when all catalyst was deactivated). Aminoalcohol **12** (1.52 mg, 0.5 mol%) was added to a solution of *trans-β*-nitrostyrene (**4**) (178.80 mg, 1.20 mmol) and propanal (**3**) (69.60 mg, 1.20 mmol) with AcOH (3.60 mg, 0.06 mmol) in toluene-d8 (0.6 mL).

When extra aminoalcohol **12** (1.52 mg, 0.5 mol%) was added to the crude reaction (with the deactivated catalytic species) none of the peaks observed in the crude reaction mixture increased and two new multiplets appeared. These two facts discarded the desilylation as a significant pathway of catalyst deactivation.

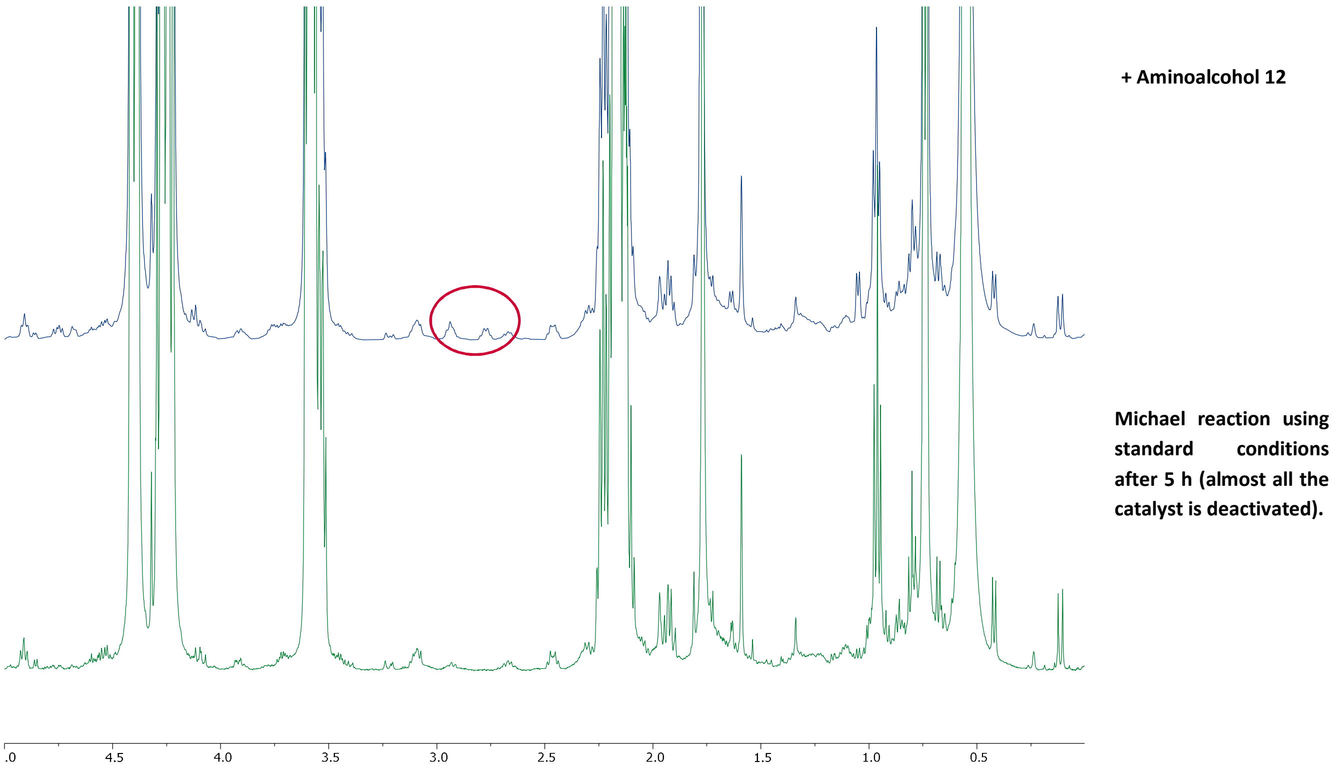

Figure S28. ^{1}H NMR spectrum (400 MHz, toluene-d8) after the addition of aminoalcohol **12** at the end of the standard reaction.

3.4.3.Deactivation pathways without desilylation of the catalyst

The catalyst can get deactivated by forming inactive, stable out-cycle species. These species could be formed by reaction of any on-cycle catalytic species with the wrong reaction partner. These could be due to the reaction of:

a) Catalyst **5** with *trans-β*-nitrostyrene (**4**).

b) The enamine of propanal with propanal (**3**) (self-condensation) or product **6** (crossed aldol).

c) The enamine of product with propanal (**3**), *trans-β*-nitrostyrene (**4**) or product **6**.

We have investigated these possible deactivation reactions by pre-mixing the corresponding reaction components and, in the case of appearance of new species, by testing their catalytic activity under reaction conditions.

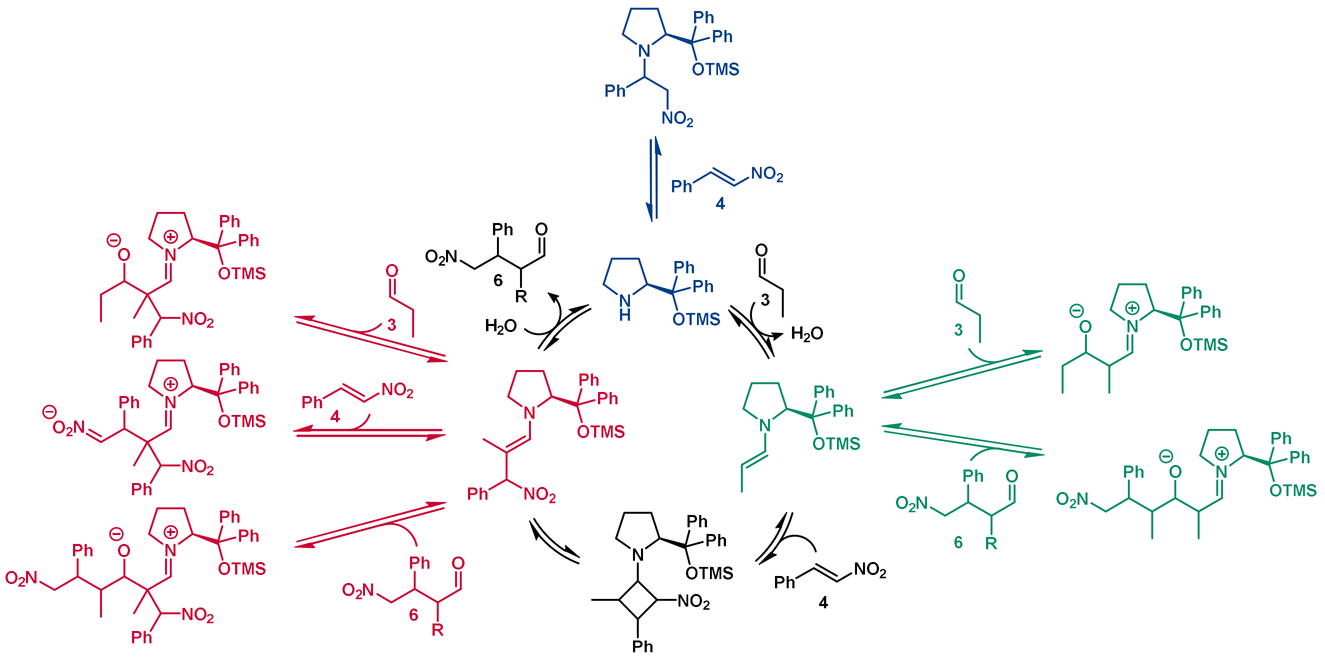

- **Catalyst 5 + *trans-β*-nitrostyrene (4)**

Catalyst **5** (1.92 mg, 0.5 mol%) was mixed separately in two NMR tubes with *trans-β*-nitrostyrene (**4**) (18.00 mg, 0.12 mmol), one in the absence of AcOH and *trans-β*-nitrostyrene (**4**) (18.00 mg, 0.12 mmol), and one with AcOH (3.60 mg, 0.06 mmol) in toluene-d8 (0.6 mL).

In the presence of AcOH, only protonated catalyst **5** was observed (**Figure S29**).

In the absence of AcOH, no reaction occurred (**Figure S30**).

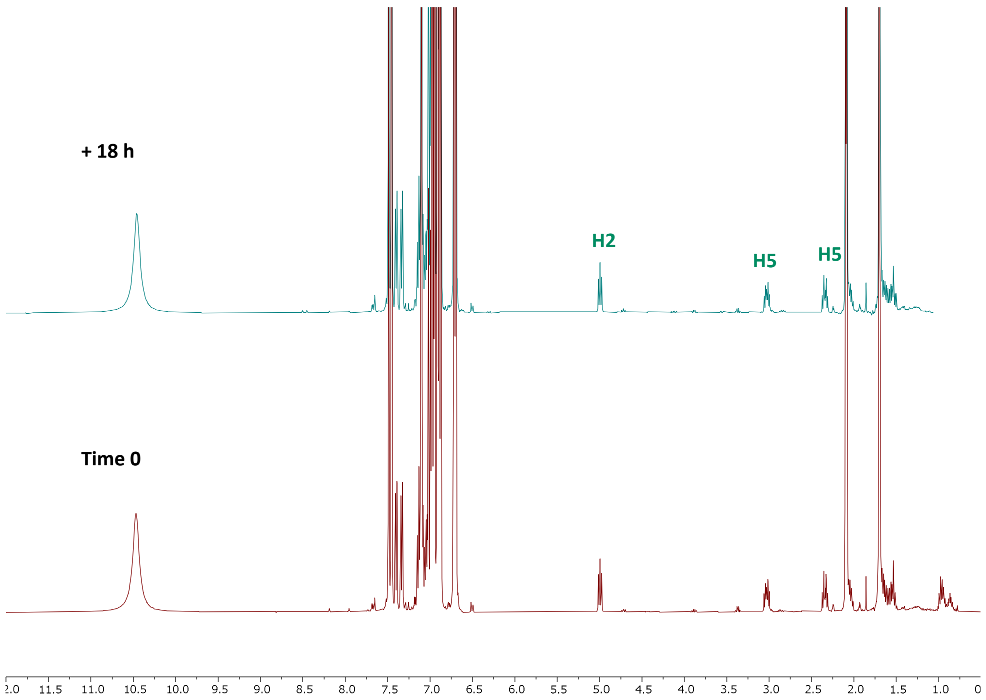

Figure S29. ^{1}H NMR spectrum (400 MHz, toluene-d8) of reaction of catalyst **5** with *trans-β*-nitrostyrene (**4**) in the presence of AcOH immediately after mixing (time 0) and after 18 h.

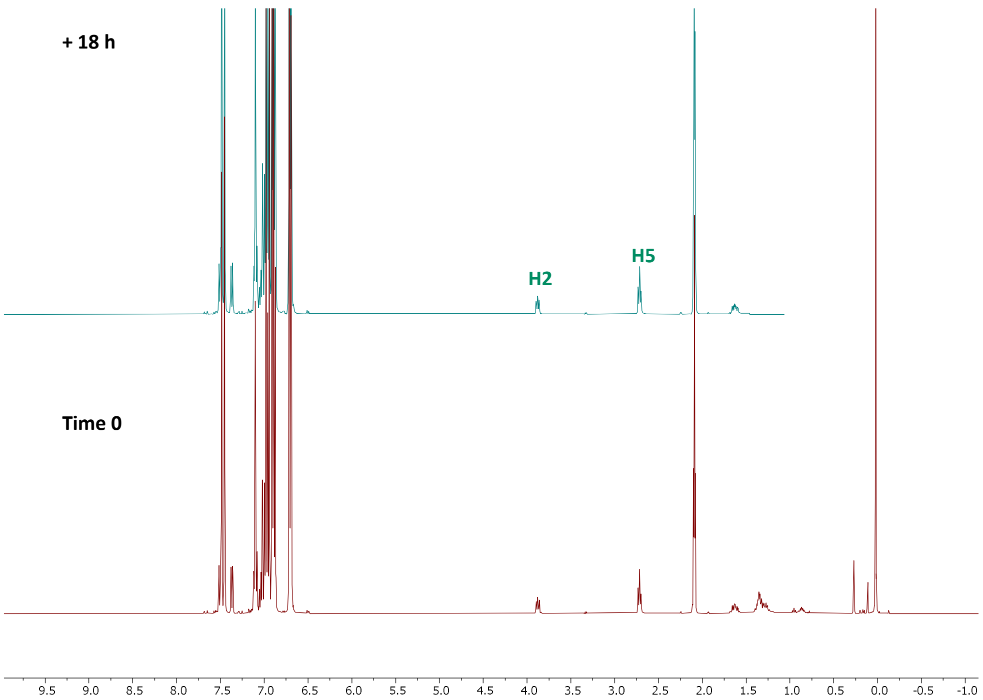

Figure S30. ^1H NMR (400 MHz, toluene-d8) spectra of reaction of catalyst **5** with *trans-β*-nitrostyrene (**4**) in the absence of AcOH immediately after mixing (time 0) and after 18 h.

These two reaction components did not react in toluene-d8 either in the absence or the presence of AcOH. Therefore, this is discarded as a deactivation pathway of the reaction.

- **Enamine of propanal + propanal (3) (self-condensation)**

Catalyst **5** (1.95 mg, 0.5 mol%) was pre-mixed during 5 h with propanal (**3**) (20.88 mg, 0.36 mmol) and AcOH (3.60 mg, 0.06 mmol) in toluene-d8 (0.6 mL).

Over time, products derived from the self-aldol reaction **16**, **17** and **10** were observed (**Figure S31**). The formation of **10** as a side-product of the aldol reaction was already mentioned by MacMillan *et al.* [5] (Check page **S-60** for the spectroscopic partial characterisation of **16**, **17** and **10**).

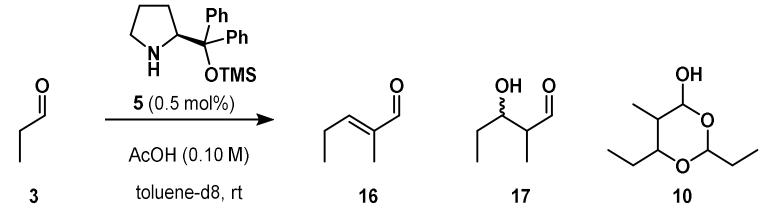

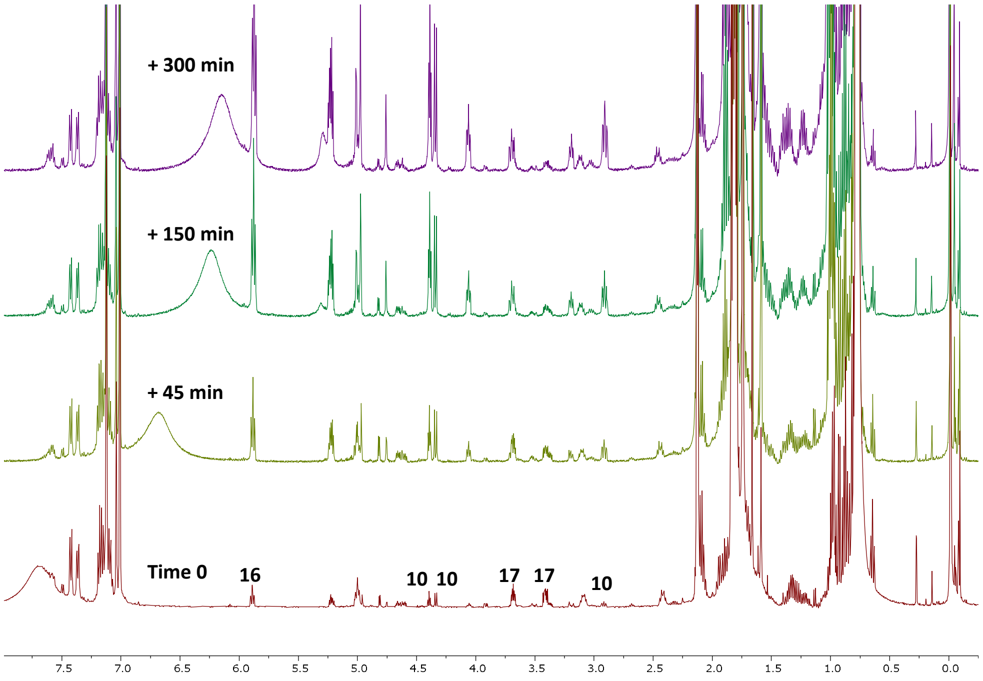

Figure S31. Evolution of the reaction of propanal (**3**) with catalyst **5** followed by ^{1}H NMR spectroscopy (500 MHz, toluene-d8).

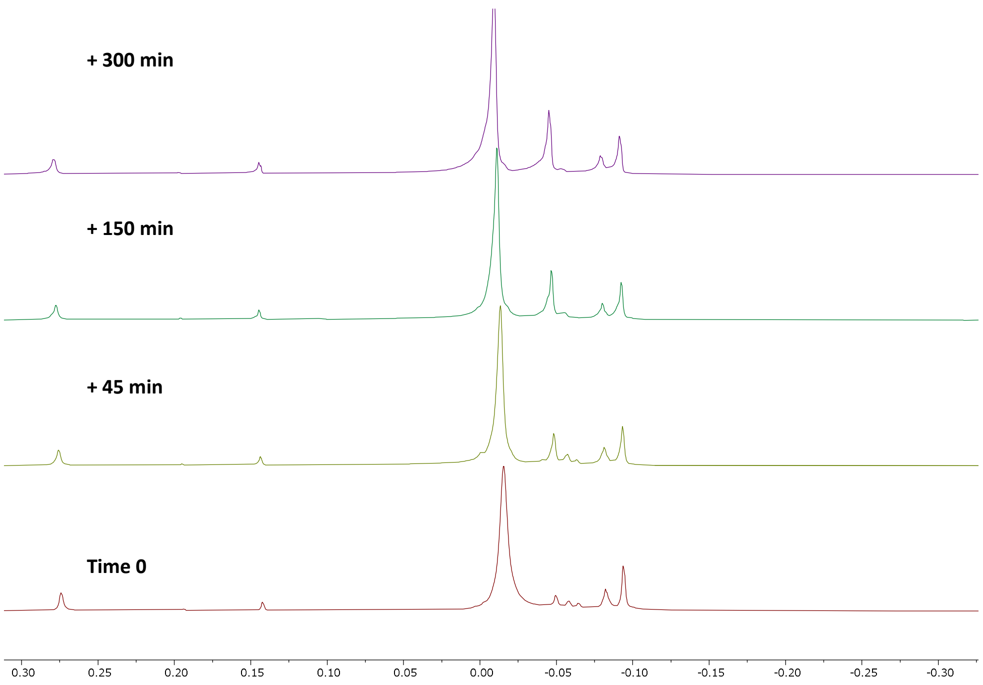

Figure S32. Evolution of the TMS signals in the reaction of propanal (**3**) with catalyst **5** followed by ^{1}H NMR spectroscopy (500 MHz, toluene-d8).

After 5 h of pre-mixing, the catalyst has been modified and new products are present (new TMS peaks increased over time **Figure S32**). Therefore, we checked the reactivity of the catalytic species under these reaction conditions by adding *trans-β*-nitrostyrene (**4**) (53.64 mg, 0.36 mmol) and the amount of propanal (**3**) consumed during self-condesation (10.30 mg, 0.18 mmol).

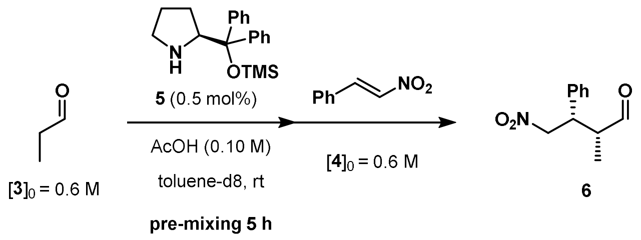

	$[3]_0$	$[4]_0$
standard reaction	2.0 M	2.0 M
same excess reaction	0.6 M	0.6 M
pre-mixing catalyst and propanal (3) (5 h)	0.6 M	0.6 M

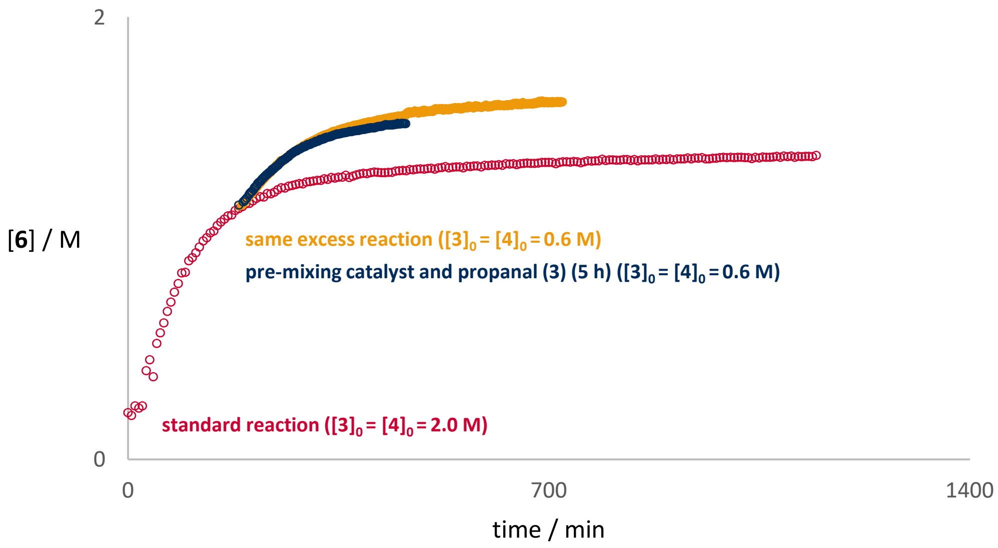

Figure S33. Kinetic profiles for the Michael reaction under different reaction conditions.

The **same excess reaction with pre-mixing propanal** (**3**) with catalyst **5** did not deactivate the catalyst due to curve overlap with the **same excess reaction** (**Figure S33**). Therefore, self-condensation side-products formation were discarded as a significant pathway of catalyst deactivation.

- **Enamine of propanal + product 6 (crossed aldol) and enamine of the product + propanal (3)**

Catalyst **5** (1.95 mg, 0.5 mol%) was pre-mixed with propanal (**3**) (20.88 mg, 0.36 mmol), product **6** (141.00 mg, 0.68 mmol) and AcOH (3.60 mg, 0.06 mmol) for 5 h in toluene-d8 (0.6 mL).

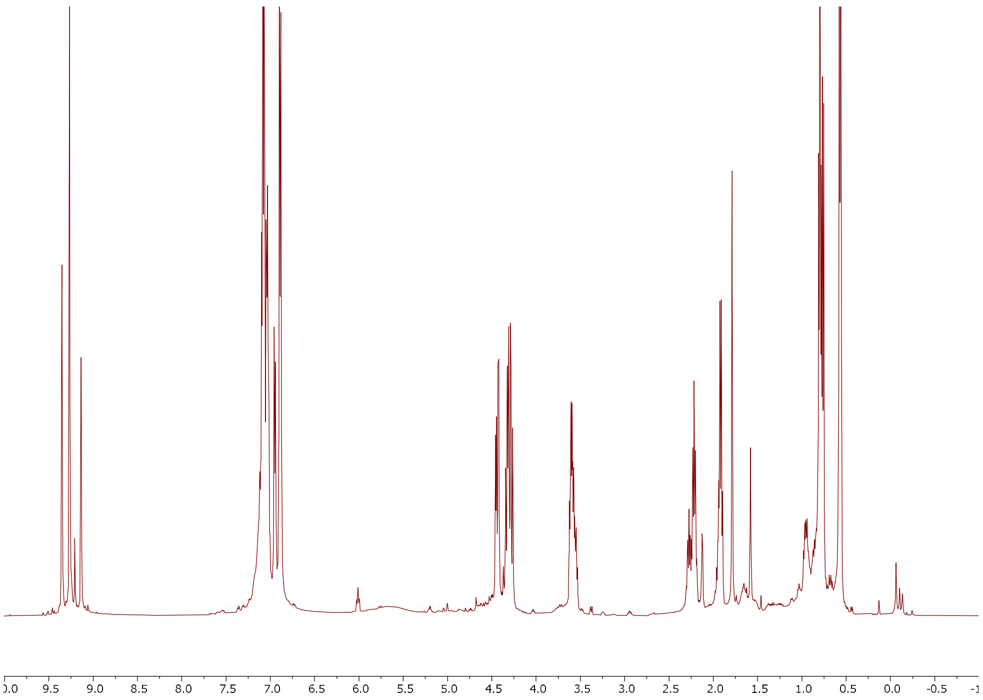

Figure S34. ^{1}H NMR spectrum (500 MHz, toluene-d8) of the reaction crude after mixing propanal (**3**) and product **6** with catalyst **5** for 5 h.

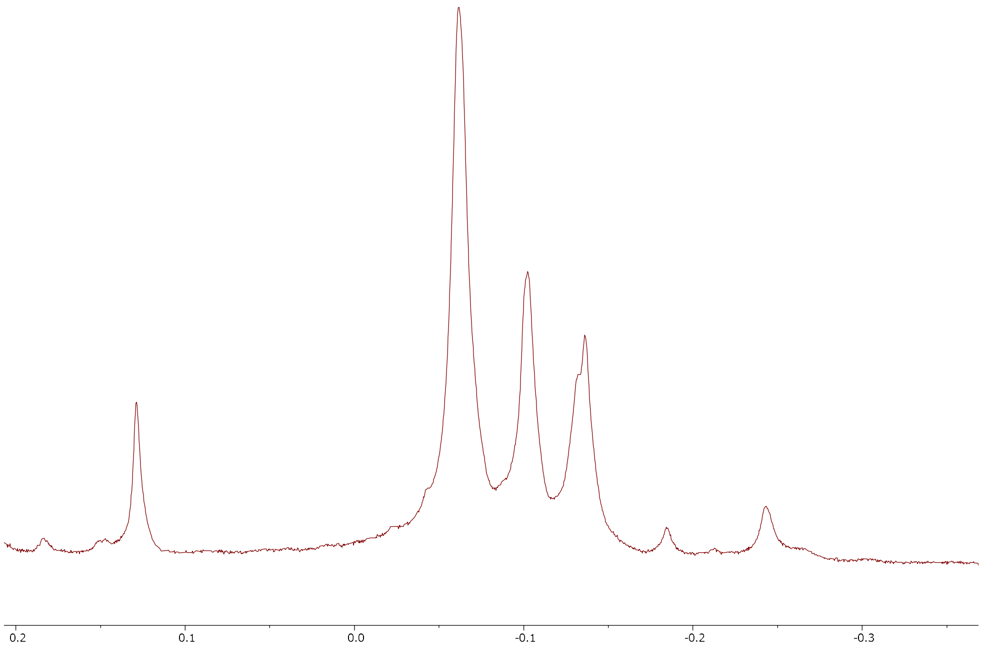

Figure S35. TMS region of the ^{1}H NMR spectrum (500 MHz, toluene-d8) of the reaction crude after mixing propanal (**3**) and product **6** with catalyst **5** for 5 h.

After 5 h of pre-mixing, the catalyst has been modified and new products are present (new TMS peaks increased over time **Figure S35**). Therefore, we checked the reactivity of the catalytic species under these reaction conditions by adding *trans-β*-nitrostyrene (**4**) (53.64 mg, 0.36 mmol).

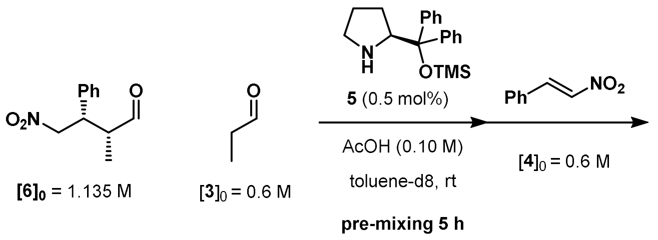

	$[\mathbf{3}]_0$	$[\mathbf{4}]_0$	$[\mathbf{6}]_0$
**standard reaction**	2.0 M	2.0 M	-
**same excess reaction**	0.6 M	0.6 M	-
**same excess reaction with product 6 added**	0.6 M	0.6 M	1.135 M
**pre-mixing catalyst, propanal (3) and product 6 (5 h)**	0.6 M	0.6 M	1.135 M

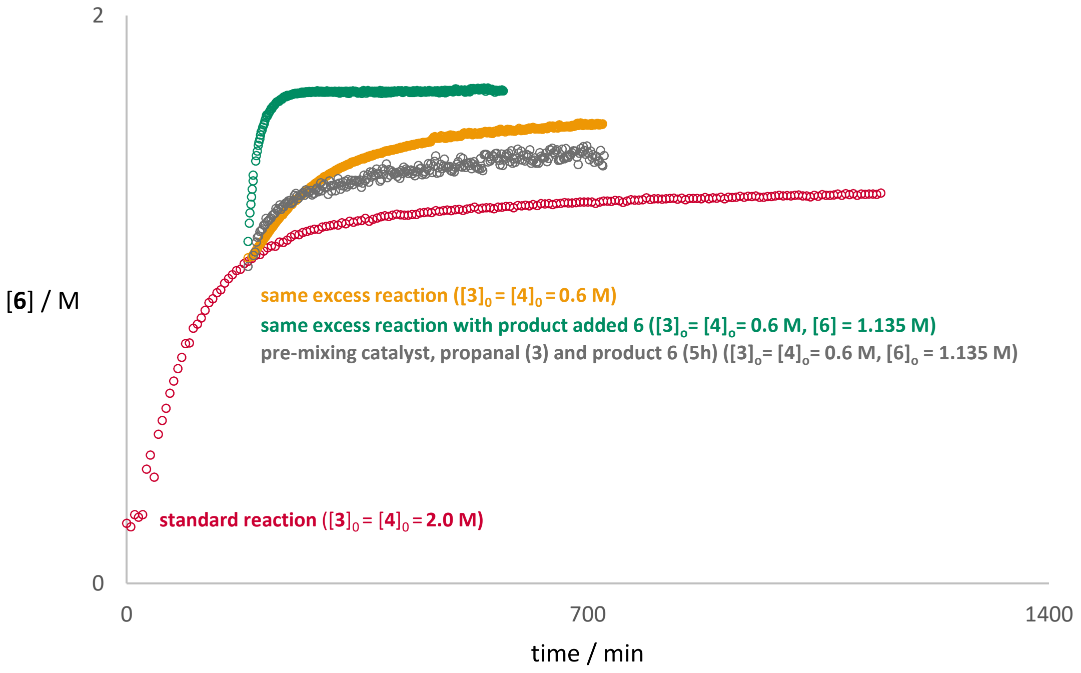

Figure S36. Kinetic profiles for the Michael reaction under different reaction conditions.

The rate of the reaction after **pre-mixing catalyst 5, propanal (3) and product 6** was clearly slower than the rate for the **same excess reaction with product added 6**, but quicker than the **standard reaction** at [**3**] = 0.6 M. These facts are consistent with a partial deactivation of the catalyst, which may be relevant during the reaction (**Figure S36**). Unfortunately, we were unable to characterise the inactive catalytic species by NMR spectroscopy.

- **Enamine of the product + *trans-β*-nitrostyrene (4) and enamine of the product + product 6**

Catalyst **5** (1.95 mg, 0.5 mol%) was mixed with product **6** (141.00 mg, 0.68 mmol), *trans-β*-nitrostyrene (**4**) (53.64 mg, 0.36 mmol) and AcOH (3.60 mg, 0.06 mmol) for 18 h in toluene-d8 (0.6 mL).

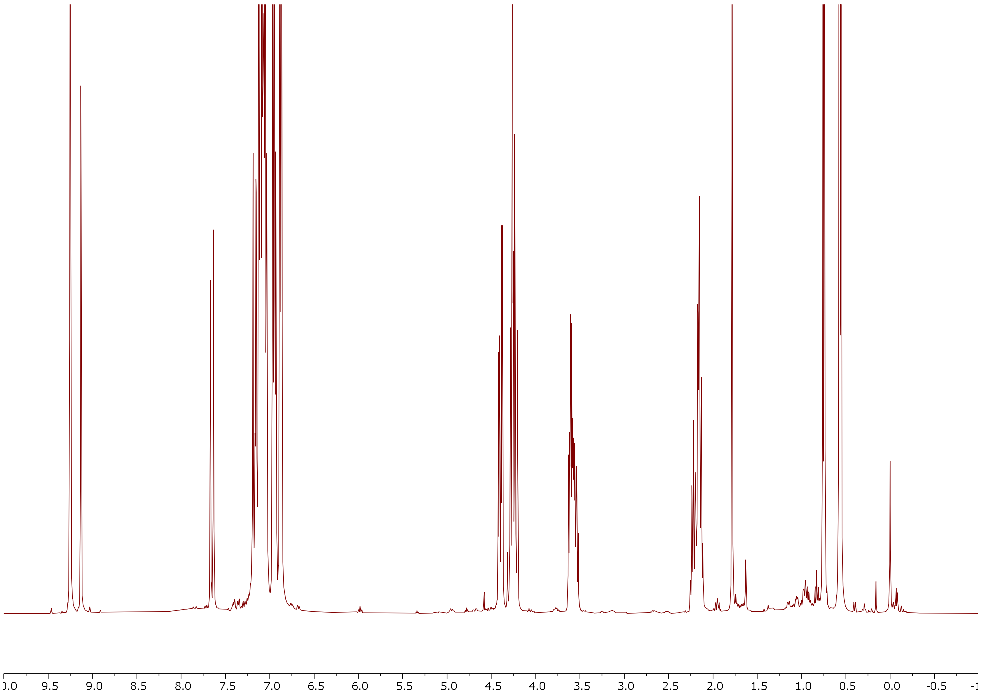

Figure S37. ^1H NMR spectrum (500 MHz, toluene-d8) of the reaction crude after mixing product **6** with *trans-β*-nitrostyrene (**4**) for 18 h.

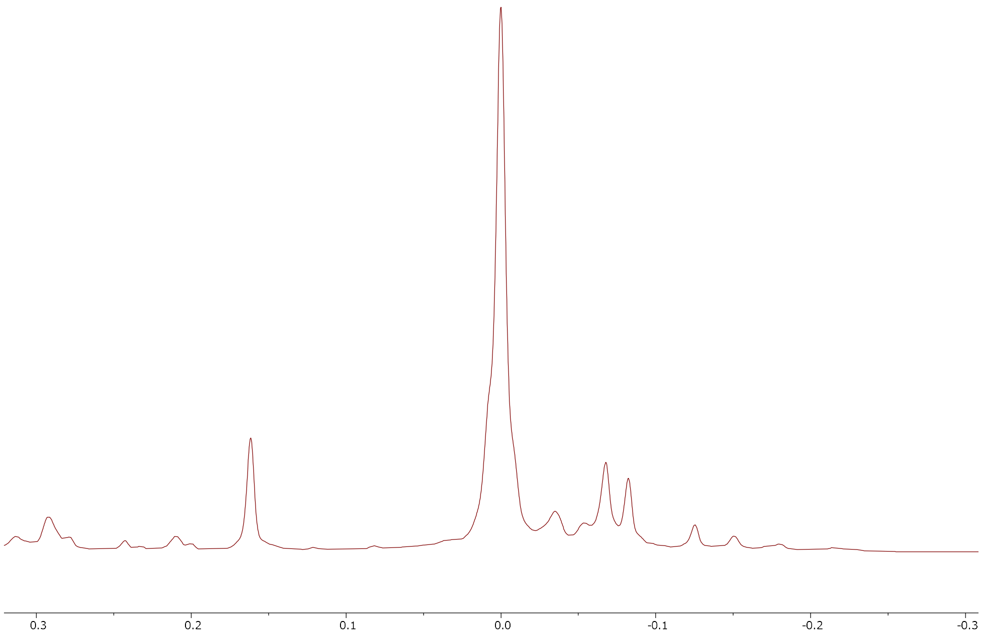

Figure S38. TMS region of ^1H NMR spectrum (500 MHz, toluene-d8) of the reaction crude after mixing product **6** with *trans-β*-nitrostyrene (**4**) for 18 h.

After 18 h of pre-mixing, it is clear that the catalyst has been modified and new products are present (new TMS peaks increased over time **Figure S38**). Therefore, we checked the reactivity of the catalytic species under these reaction conditions by adding propanal (**3**) (20.88 mg, 0.36 mmol).

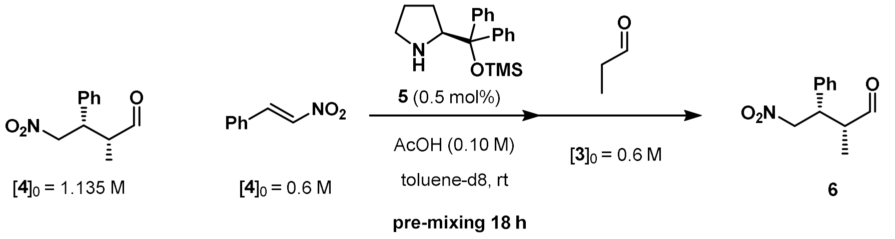

	$[3]_0$	$[4]_0$	$[6]_0$
standard reaction	2.0 M	2.0 M	-
same excess reaction with product 6 added	0.6 M	0.6 M	1.135 M
pre-mixing catalyst, *trans-β*-nitrostyrene (4) and product 6 (18 h)	0.6 M	0.6 M	1.135 M
pre-mixing catalyst, propanal (3) and product (5 h)	0.6 M	0.6 M	1.135 M

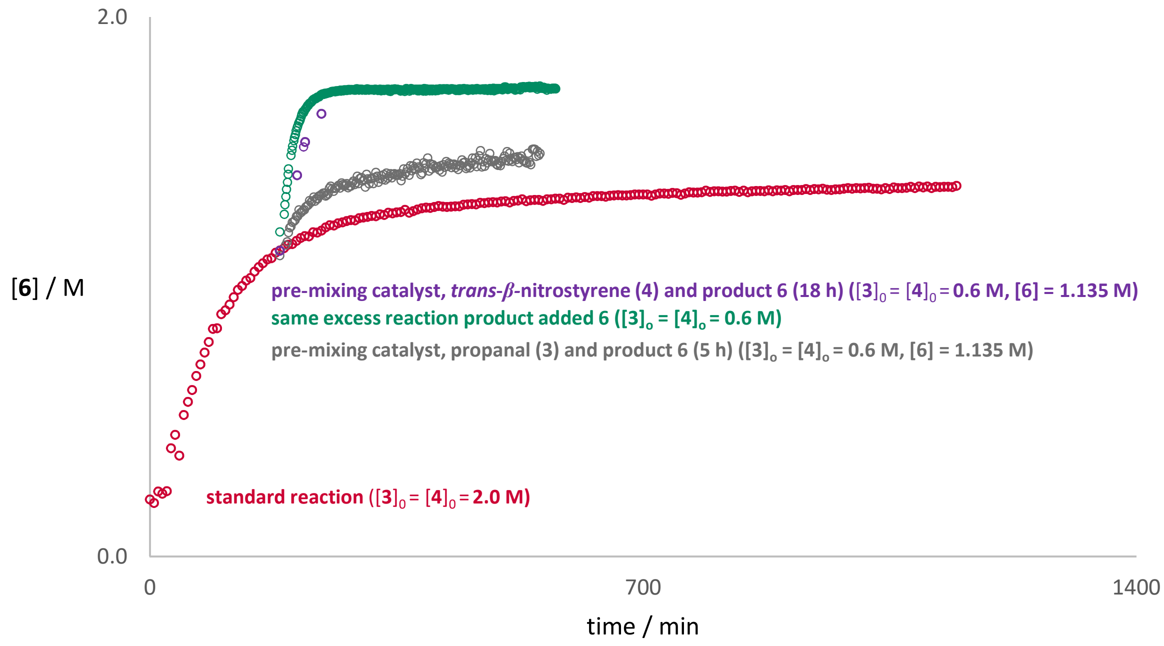

Figure S39. Kinetic profiles for the Michael reaction under different reaction conditions.

The rate of the reaction after **pre-mixing the catalyst 5 with product 6 and *trans-β*-nitrostyrene (4)** overlays with the rate of the reaction for the **same excess reaction with product added** (**Figure S39**). Therefore, the formation of side-products from the reaction of the enamine of product **6** with *trans-β*-nitrostyrene (**4**) or the reaction of the enamine of product **6** with product **6** were discarded as a significant pathway of catalyst deactivation.

Pre-mixing catalyst **5** with all the possible combinations of reaction components for 5 h did not deactivate the catalyst to the same degree as observed in the reaction. This observation suggests that the major pathways of deactivation, operating during the reaction under standard conditions, may also involve a catalytic intermediate different from the free catalyst, enamine of the propanal, and enamine of the product.

Another important catalytic intermediate in this reaction is the cyclobutane **18**, which is in equilibrium with the putative zwitterionic iminium nitronate **7**.[6] Intermediate **7** can only exist as a transient species during the reaction and it is very unlikely to be formed by mixing catalyst **5** and product **6**. Intermediate **7** could react with propanal (**3**), *trans-β*-nitrostyrene (**4**) and product **6** forming very stable 6-member ring cycles (**8**, **9** and **19**), which would trap the catalyst.

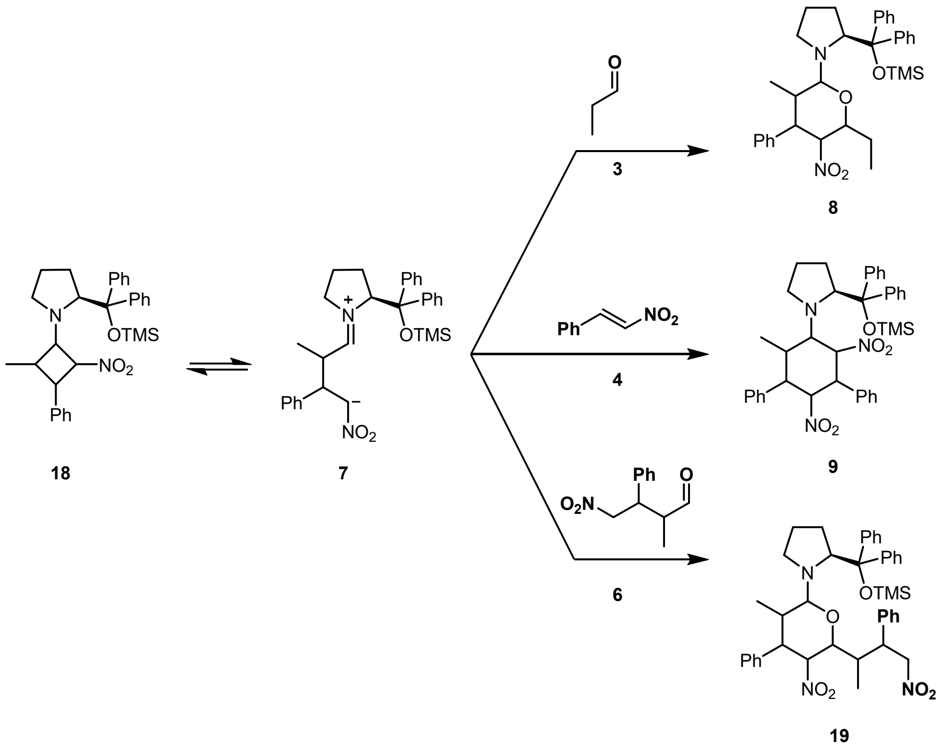

Due to the small amount of catalyst used in the standard reaction and the variety of possible diastereoisomers of products **8**, **9** and **19** we have been unable to confirm the presence of these intermediates by NMR (spectra were recorded on a Bruker AVIII 800 spectrometer equipped with a TCI helium-cooled cryoprobe). Therefore, we decided to monitor the reaction by accurate MS.

- **Reaction monitoring by accurate MS**

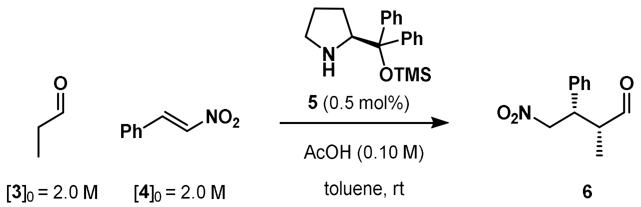

Catalyst **5** (6.50 mg, 0.5 mol%) was added to a solution of *trans-β*-nitrostyrene (**4**) (596.00 mg, 1.20 mmol) and propanal (**3**) (232.00 mg, 1.20 mmol) with AcOH (12.00 mg, 0.06 mmol) in toluene (2.0 mL). Over time, reaction samples of 100 μL were taken from the reaction flask followed by dilution with 1.7 mL of toluene. The vial was then placed in the HPLC plate and immediately transferring 10 μL from the diluted vial to the injection port of the HPLC to analyse accurate MS.

Relevant HESI method parameters

- Sheath gas flow rate: 6
- Aux gas flow rate: 2
- Sweep gas flow rate: 0
- Spray voltage (kV): 3.50
- Capillary temp.: 200 °C
- S-lens RF level: 50.0
- Aux gas heater temp.: 350 °C
- Flow rate: 0.2 mL/min

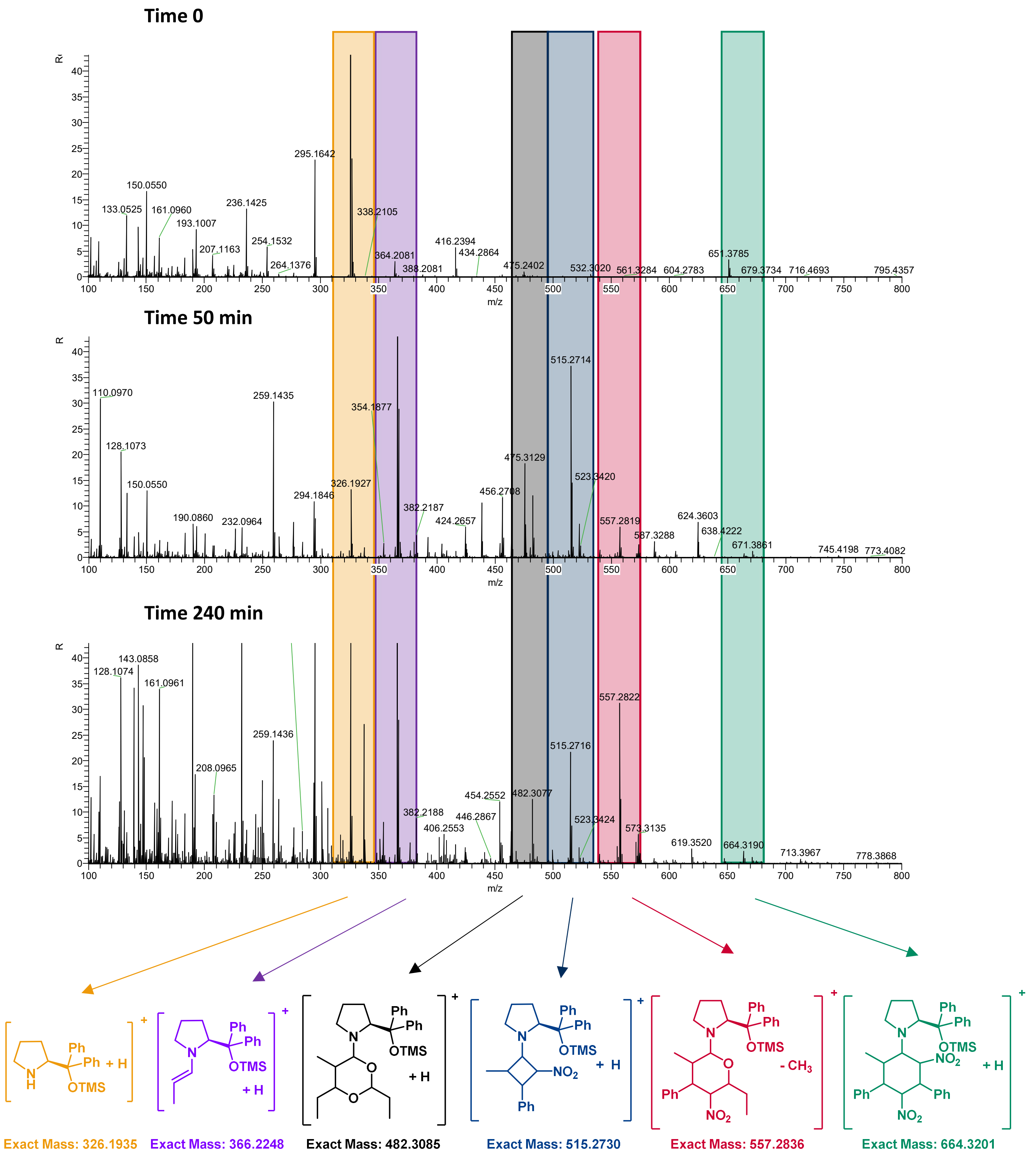
Time 0
295.1642
150.0550
133.0525
161.0960
193.1007
236.1425
338.2105
207.1163
254.1532
264.1376
364.2081
388.2081
416.2394
434.2864
475.2402
532.3020
561.3284
604.2783
651.3785
679.3734
716.4693
795.4357
m/z
Time 50 min
110.0970
128.1073
150.0550
190.0860
232.0964
259.1435
294.1846
326.1927
354.1877
382.2187
424.2657
456.2708
475.3129
515.2714
523.3420
557.2819
587.3288
624.3603
638.4222
671.3861
745.4198
773.4082
Time 240 min
143.0858
128.1074
161.0961
208.0965
259.1436
382.2188
406.2553
446.2867
454.2552
482.3077
515.2716
523.3424
557.2822
573.3135
619.3520
664.3190
713.3967
778.3868
Ph
OTMS
+ H
N
H
Ph
OTMS
NO2
- CH3
O
Exact Mass: 326.1935
Exact Mass: 366.2248
Exact Mass: 482.3085
Exact Mass: 515.2730
Exact Mass: 557.2836
Exact Mass: 664.3201

Time 0

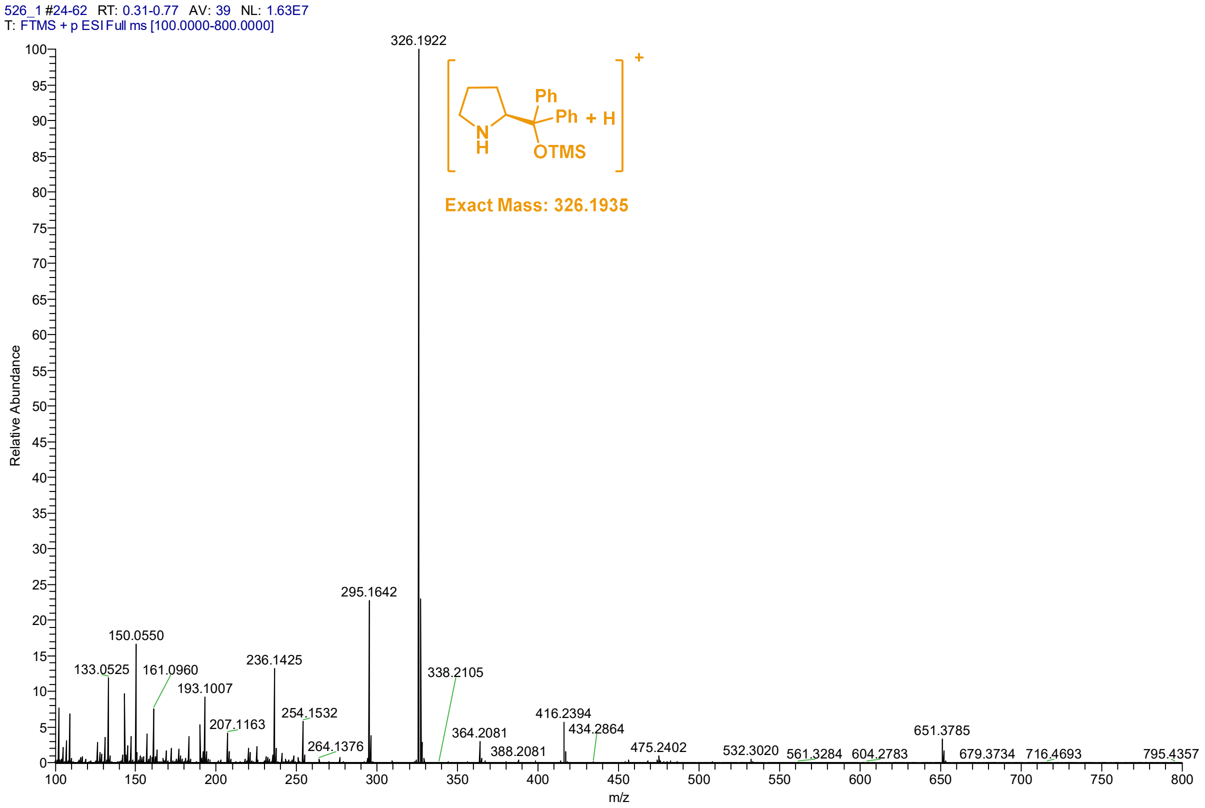

526_1 #24-62 RT: 0.31-0.77 AV: 39 NL: 1.63E7
T: FTMS + p ESI Full ms [100.0000-800.0000]
Relative Abundance
m/z
326.1922
295.1642
150.0550
133.0525
161.0960
193.1007
236.1425
338.2105
207.1163
254.1532
264.1376
364.2081
388.2081
416.2394
434.2864
475.2402
532.3020
561.3284
604.2783
651.3785
679.3734
716.4693
795.4357
Ph
Ph + H
N
H
OTMS
+
Exact Mass: 326.1935

Time 0 + Propanal (**3**)

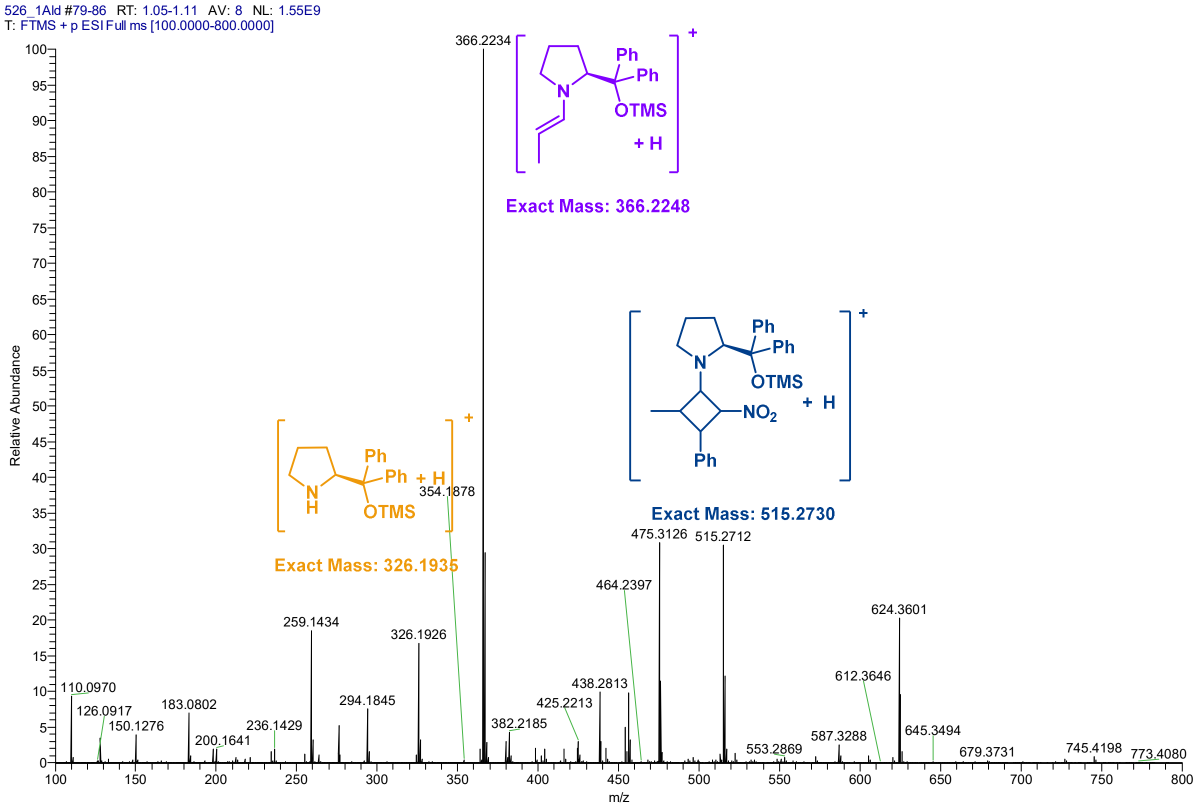

Time 10 min

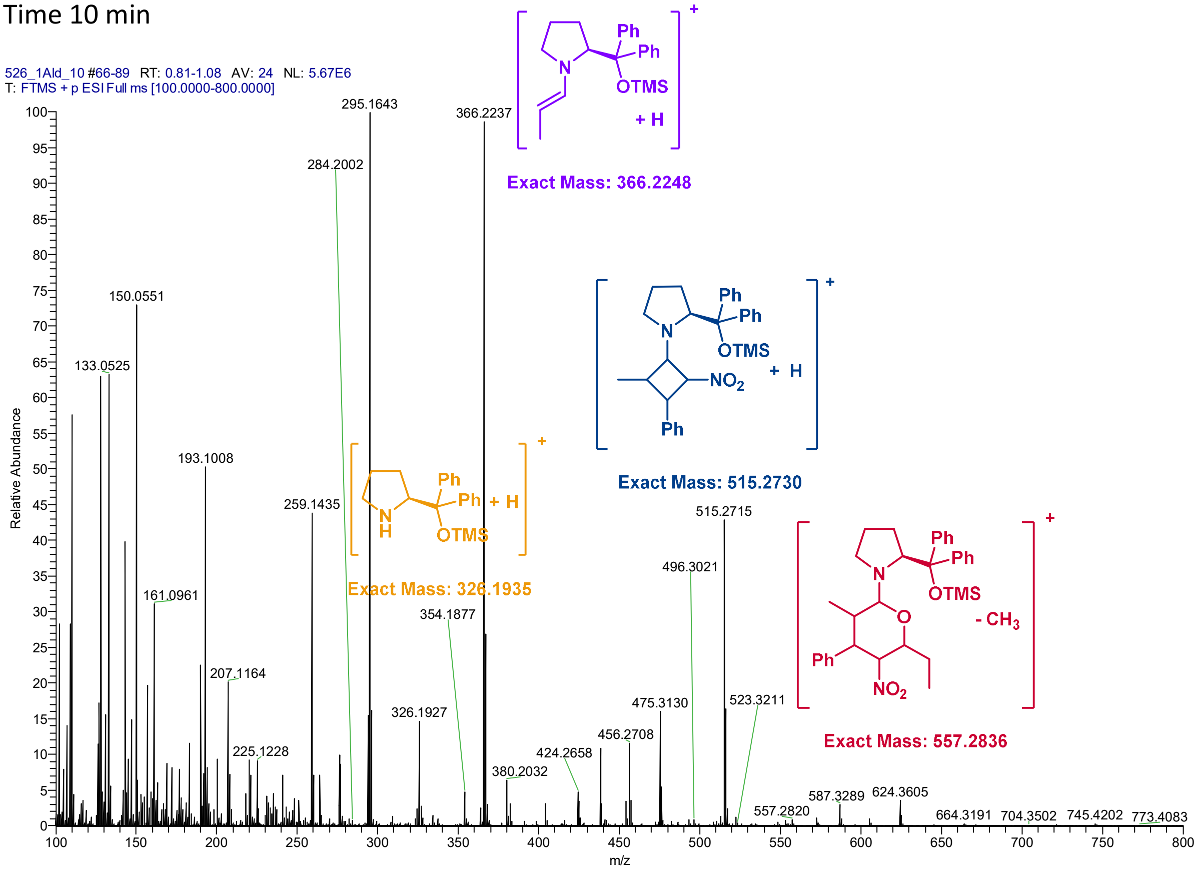
526_1Ald_10 #66-89 RT: 0.81-1.08 AV: 24 NL: 5.67E6
T: FTMS + p ESI Full ms [100.0000-800.0000]
Relative Abundance
m/z
295.1643
366.2237
284.2002
150.0551
133.0525
193.1008
161.0961
207.1164
225.1228
259.1435
326.1927
354.1877
380.2032
424.2658
456.2708
475.3130
496.3021
515.2715
523.3211
557.2820
587.3289
624.3605
664.3191
704.3502
745.4202
773.4083
Exact Mass: 366.2248
Exact Mass: 515.2730
Exact Mass: 326.1935
Exact Mass: 557.2836

Time 50 min

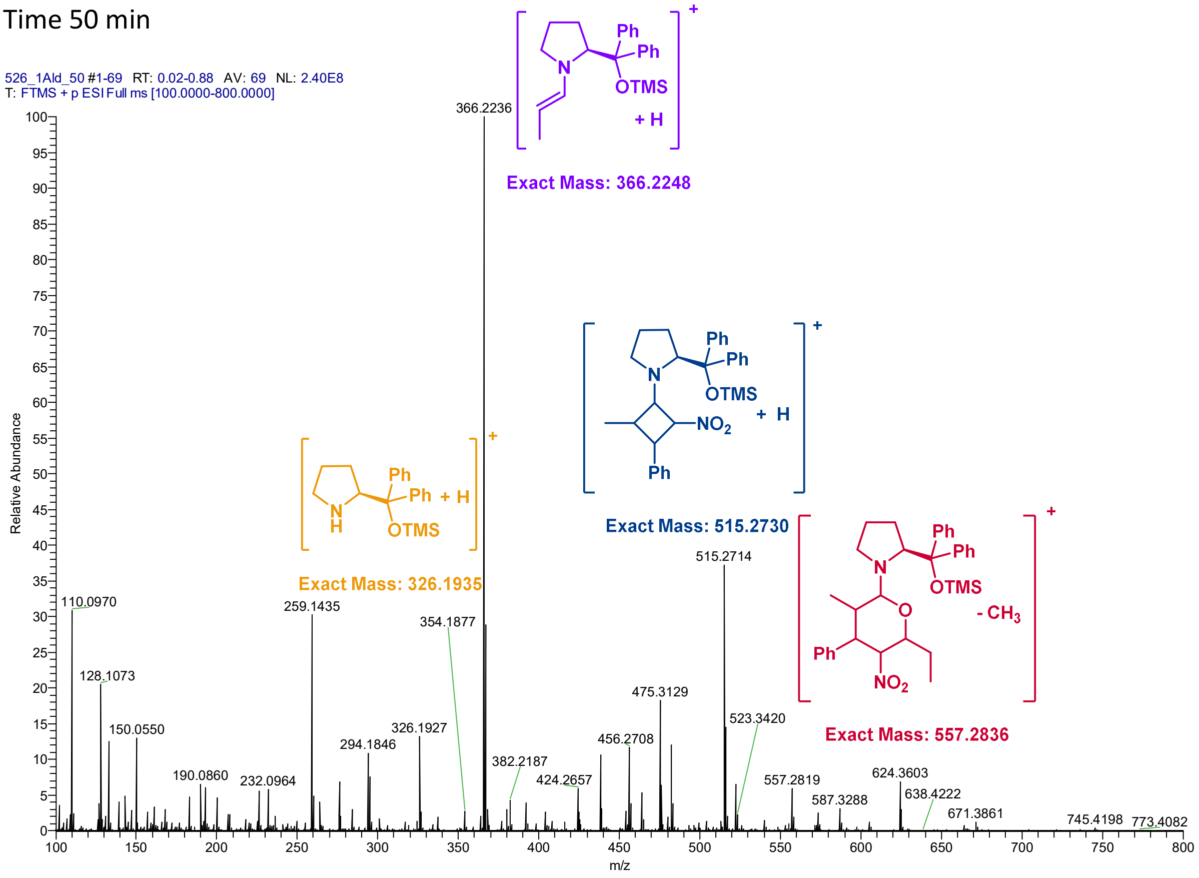
526_1Ald_50 #1-69 RT: 0.02-0.88 AV: 69 NL: 2.40E8
T: FTMS + p ESI Full ms [100.0000-800.0000]
Relative Abundance
m/z
366.2236
515.2714
475.3129
259.1435
110.0970
354.1877
128.1073
150.0550
326.1927
294.1846
456.2708
523.3420
382.2187
424.2657
190.0860
232.0964
557.2819
587.3288
624.3603
638.4222
671.3861
745.4198
773.4082
Ph
Ph
N
OTMS
+ H
+
Exact Mass: 366.2248
Ph
Ph
N
OTMS
NO_2
+ H
Ph
+
Exact Mass: 515.2730
Ph
Ph
N
H
OTMS
+ H
+
Exact Mass: 326.1935
Ph
Ph
N
OTMS
O
- CH_3
Ph
NO_2
+
Exact Mass: 557.2836

Time 120 min

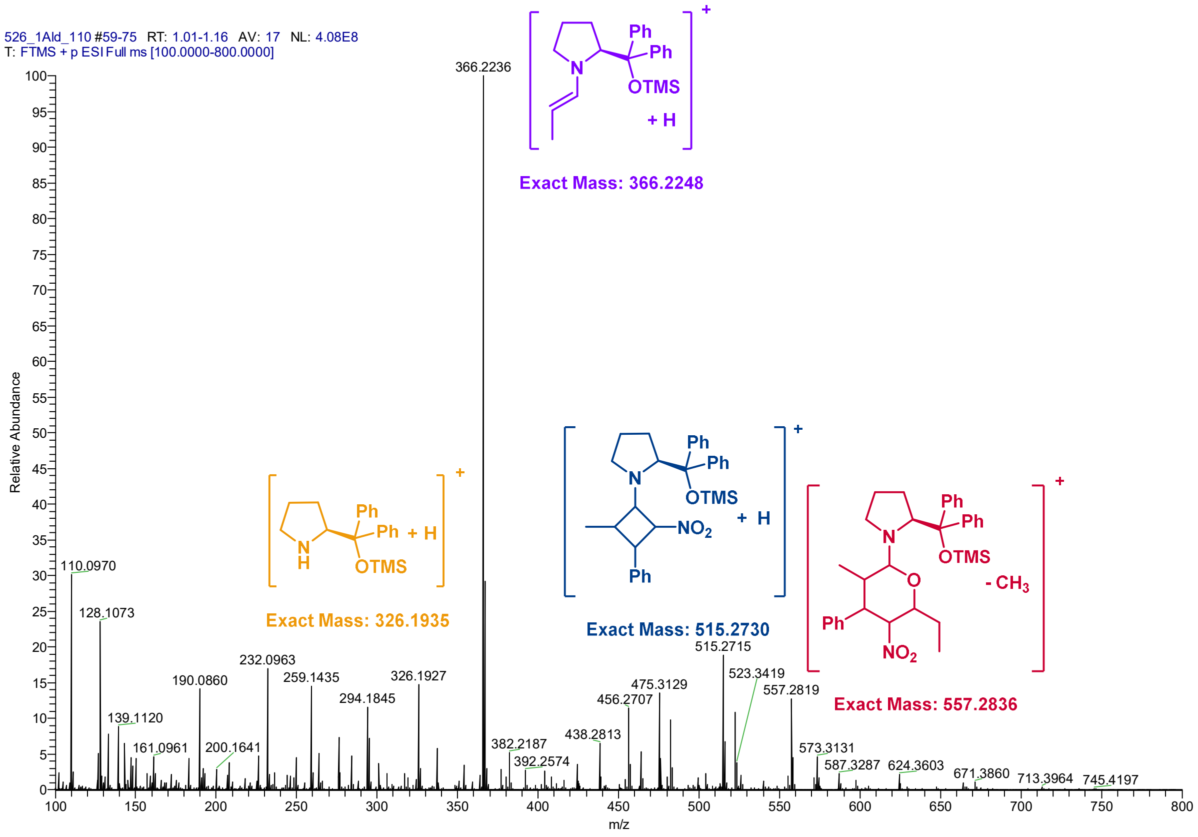

526_1Ald_110 #59-75 RT: 1.01-1.16 AV: 17 NL: 4.08E8
T: FTMS + p ESI Full ms [100.0000-800.0000]
Relative Abundance
m/z
366.2236
110.0970
128.1073
139.1120
161.0961
190.0860
200.1641
232.0963
259.1435
294.1845
326.1927
382.2187
392.2574
438.2813
456.2707
475.3129
515.2715
523.3419
557.2819
573.3131
587.3287
624.3603
671.3860
713.3964
745.4197
Exact Mass: 366.2248
Exact Mass: 326.1935
Exact Mass: 515.2730
Exact Mass: 557.2836

Time 180 min

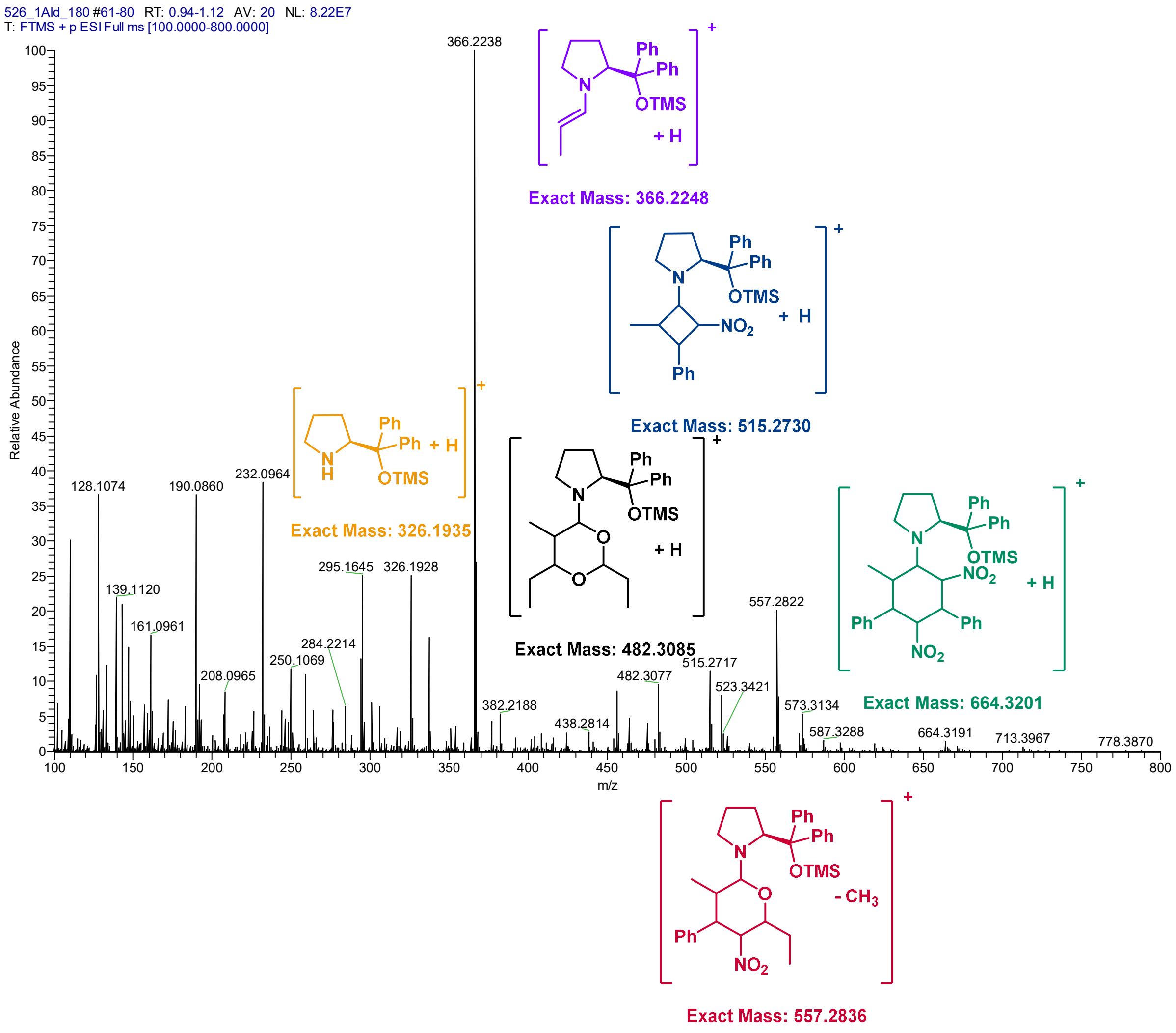

526_1Ald_180 #61-80 RT: 0.94-1.12 AV: 20 NL: 8.22E7
T: FTMS + p ESI Full ms [100.0000-800.0000]
Relative Abundance
m/z
366.2238
128.1074
190.0860
232.0964
139.1120
161.0961
208.0965
250.1069
284.2214
295.1645
326.1928
382.2188
438.2814
482.3077
515.2717
523.3421
557.2822
573.3134
587.3288
664.3191
713.3967
778.3870
Exact Mass: 366.2248
Exact Mass: 515.2730
Exact Mass: 326.1935
Exact Mass: 482.3085
Exact Mass: 664.3201
Exact Mass: 557.2836

Time 240 min

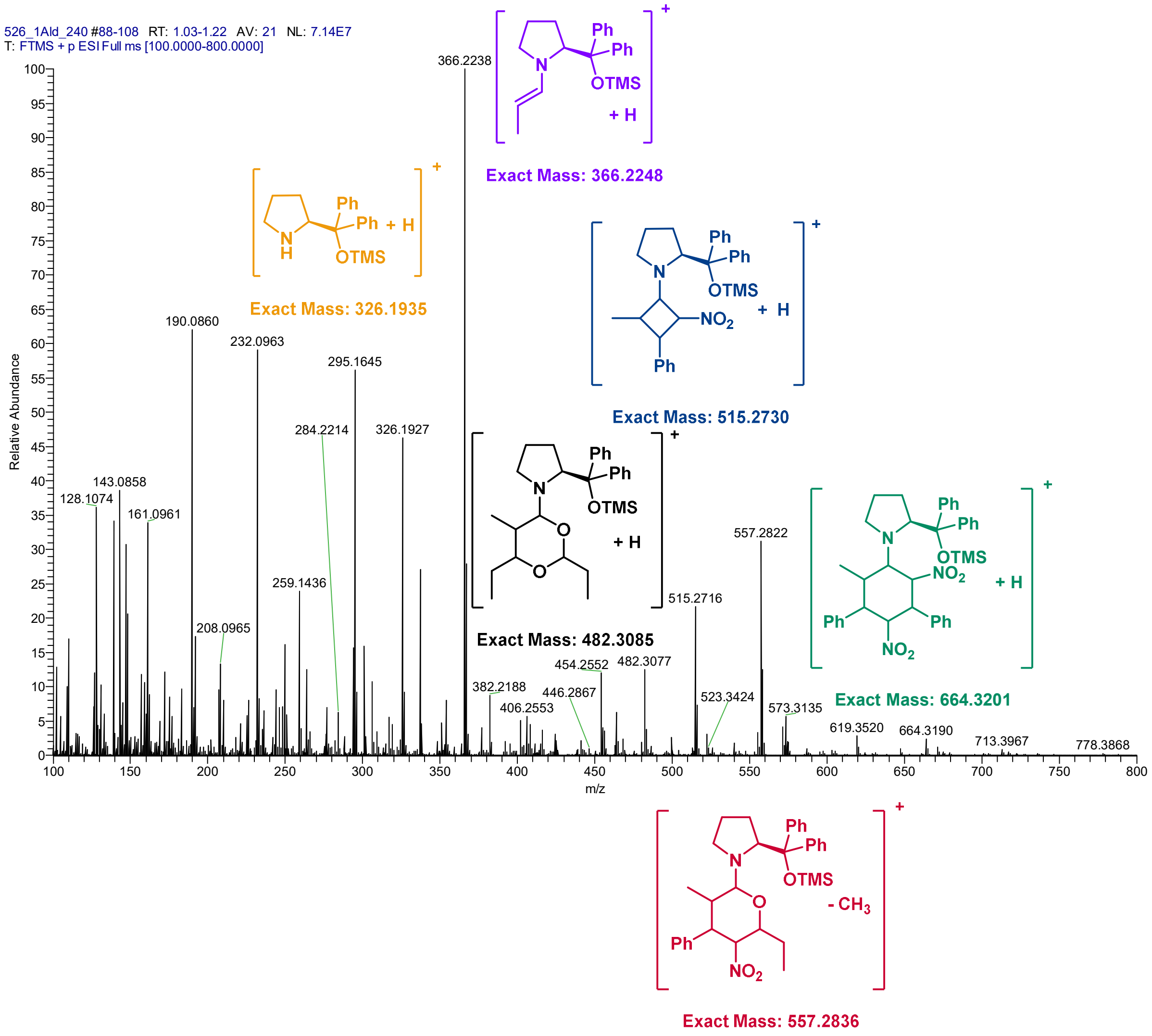
526_1Ald_240 #88-108 RT: 1.03-1.22 AV: 21 NL: 7.14E7
T: FTMS + p ESI Full ms [100.0000-800.0000]
Relative Abundance
m/z
366.2238
190.0860
232.0963
295.1645
284.2214
326.1927
143.0858
128.1074
161.0961
259.1436
208.0965
382.2188
406.2553
446.2867
454.2552
482.3077
515.2716
523.3424
557.2822
573.3135
619.3520
664.3190
713.3967
778.3868
Exact Mass: 366.2248
Exact Mass: 326.1935
Exact Mass: 515.2730
Exact Mass: 482.3085
Exact Mass: 664.3201
Exact Mass: 557.2836

Time 18 h

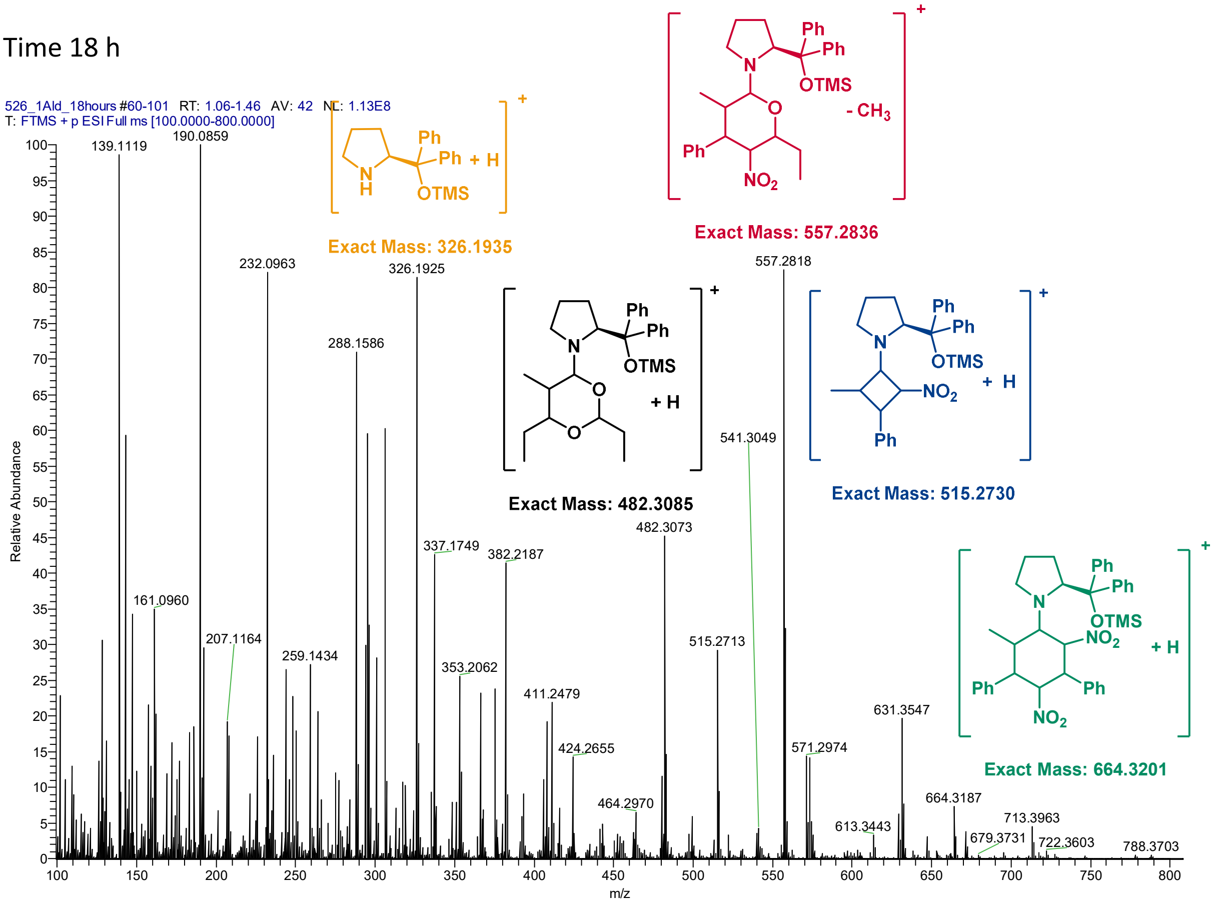
526_1Ald_18hours #60-101 RT: 1.06-1.46 AV: 42 NL: 1.13E8
T: FTMS + p ESI Full ms [100.0000-800.0000]
Relative Abundance
m/z
139.1119
190.0859
232.0963
326.1925
288.1586
557.2818
541.3049
482.3073
337.1749
382.2187
161.0960
207.1164
259.1434
353.2062
411.2479
424.2655
464.2970
515.2713
571.2974
631.3547
613.3443
664.3187
679.3731
713.3963
722.3603
788.3703
Exact Mass: 326.1935
Exact Mass: 557.2836
Exact Mass: 482.3085
Exact Mass: 515.2730
Exact Mass: 664.3201
OTMS
- CH3
+ H

Partial characterisation of side products 16, 17 and 10

- **Aldehyde 16**[7]

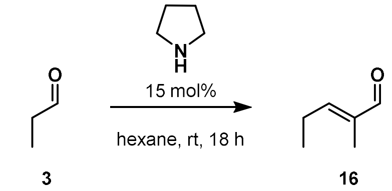

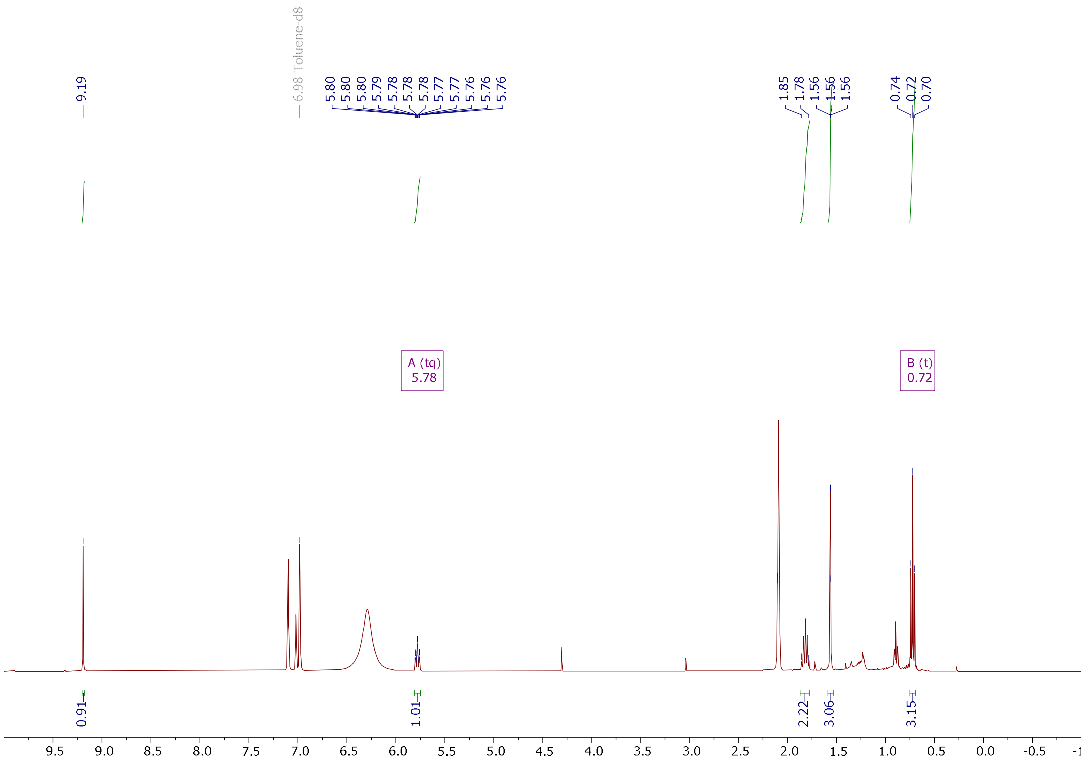

Figure S40. ^{1}H NMR spectrum (400 MHz, toluene-d8) of aldehyde **16**.

- **Aldol 17**[8]

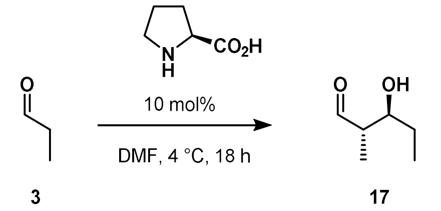

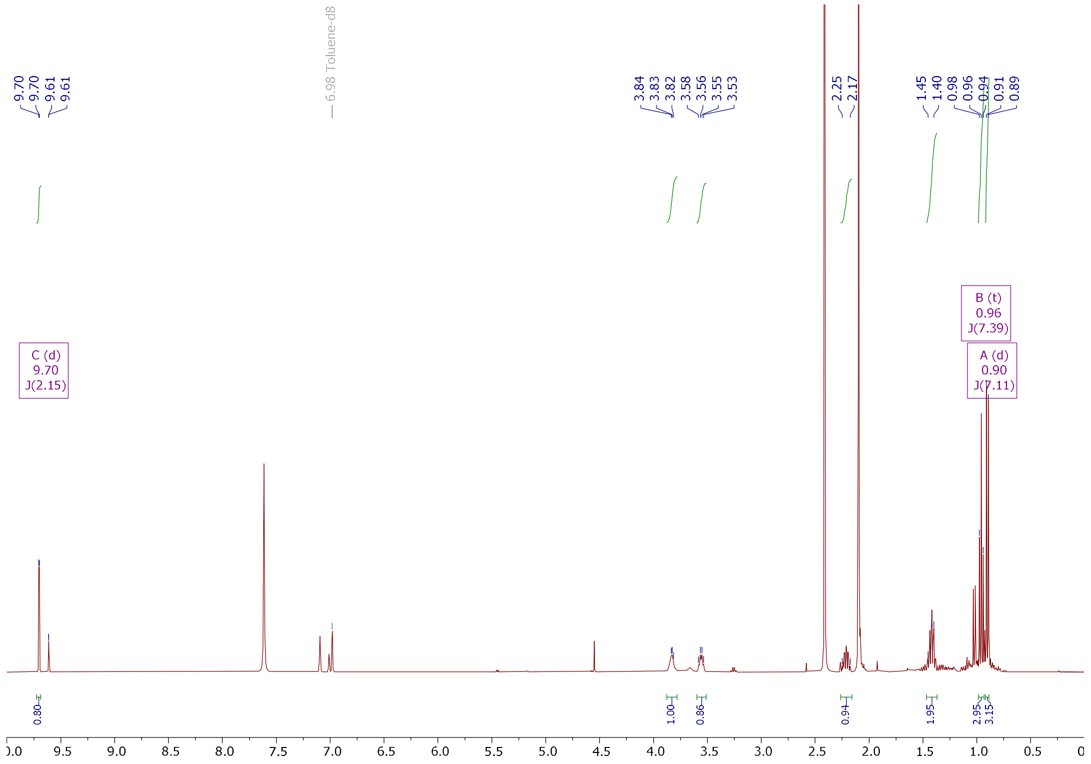

Figure S41. ^{1}H NMR spectrum (400 MHz, toluene-d8) of aldol **17**.

- **1,3-Dioxane 10**

1,3-Dioxane **10** was formed by mixing aldol **17** (18.00 mg, 0.16 mmol) with an excess of propanal (**3**) (46.40 mg, 0.80 mmol) followed by dropwise addition of glacial AcOH to accelerate formation of the 6-membered ring **10**.

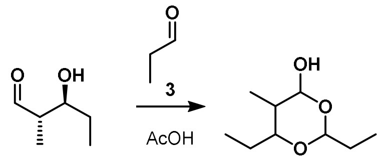

17 **10**

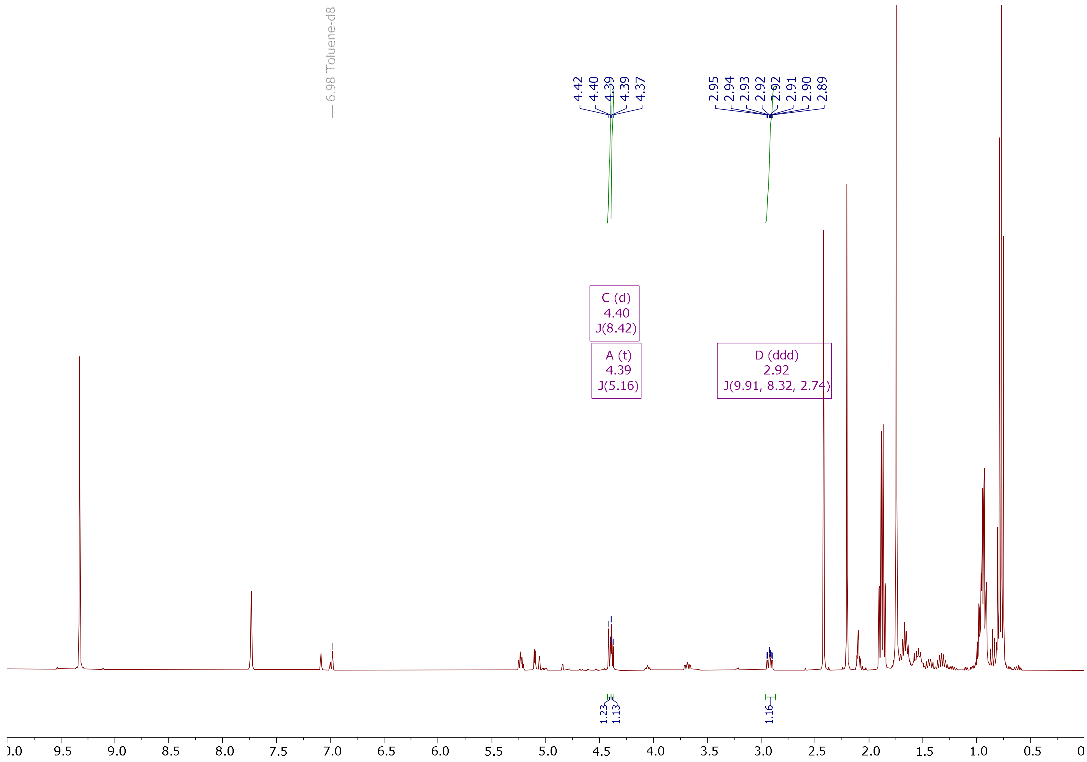

Figure S42. ^{1}H NMR spectrum (400 MHz, toluene-d8) of 1,3-dioxane **10**.

1,3-Dioxane **10** is formed during the Michael reaction. Catalyst **5** (1.95 mg, 0.5 mol%) was added to a solution of propanal (**3**) (69.60 mg, 1.20 mmol) with AcOH (3.60 mg, 0.06 mmol) in toluene-d8 (0.6 mL) in an NMR tube. The solution was then passed through silica with the aim of removing proline species, leaving **10** as a major side-product. 1,3-Dioxane **10** was characterised by 2D NMR, including COSY, HSQC and HMBC.

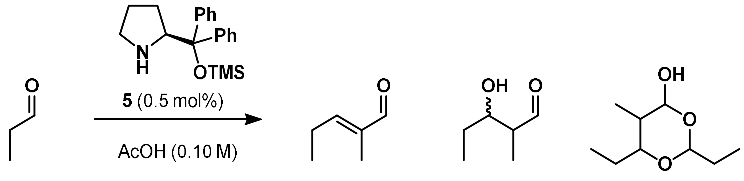

3 **16** **17** **10**

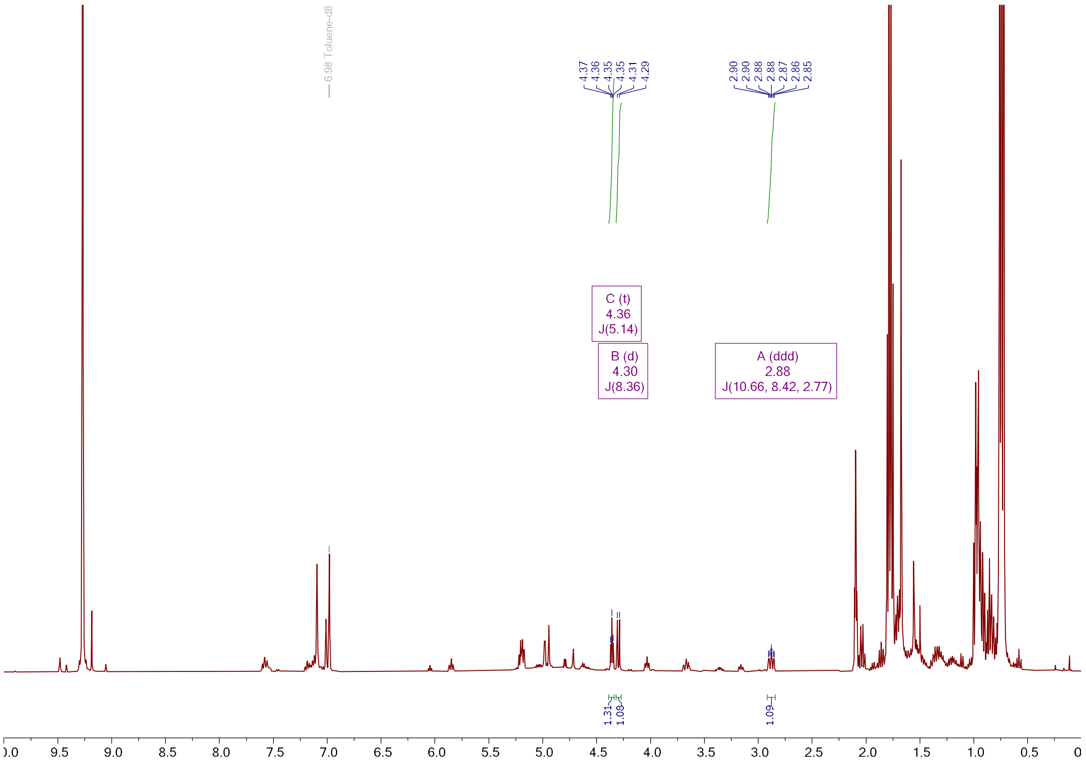

Figure S43. ^{1}H NMR spectrum (400 MHz, toluene-d8) of 1,3-dioxane **10**.

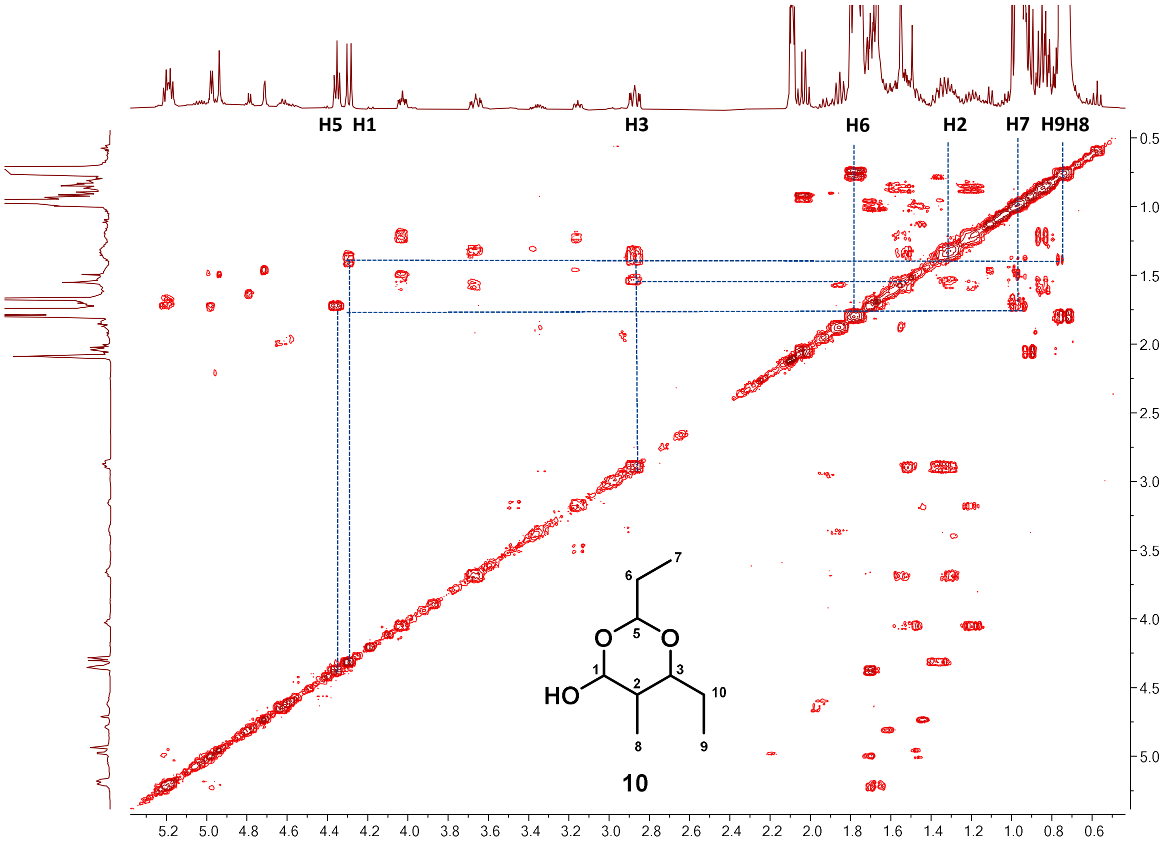

Figure S44. COSY spectrum (400 MHz, toluene-d8) of 1,3-dioxane **10**.

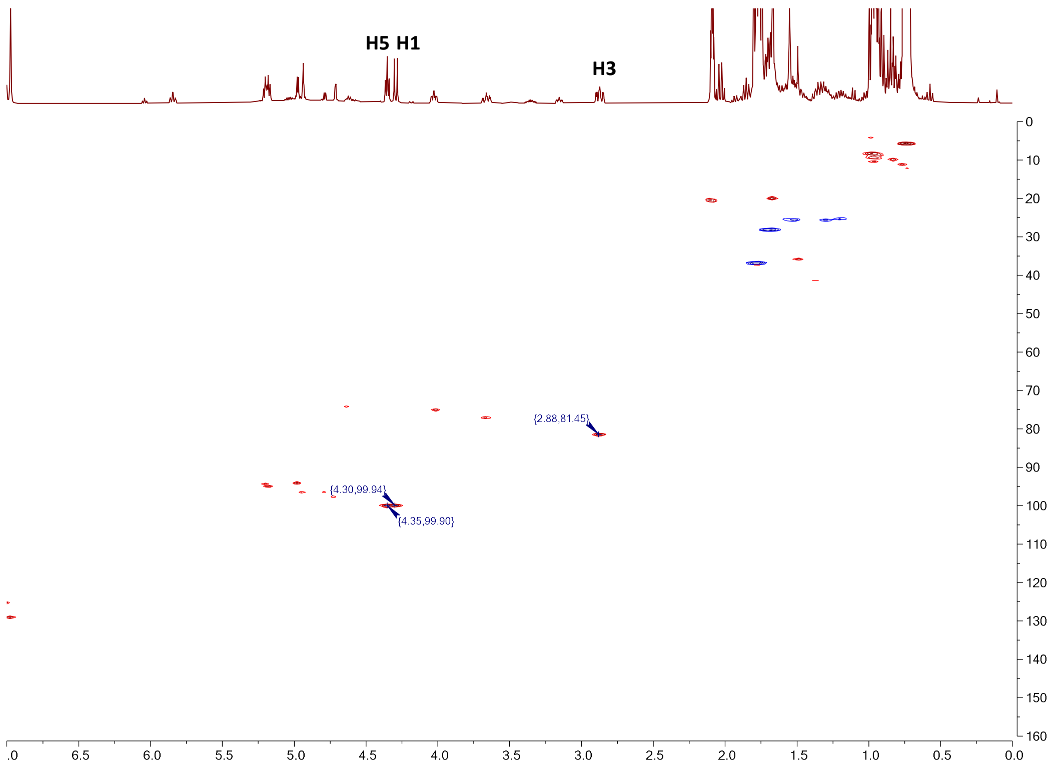

Figure S45. HSQC spectrum (400 MHz, toluene-d8) of 1,3-dioxane **10**.

References

[1] A. Vidal-Ferran, I. Mon, A. Bauzá, A. Frontera, L. Rovira, *Chem.- Eur. J.* **2015**, *21*, 11417.

[2] L. Carreras, L. Rovira, M. Vaquero, I. Mon, E. Martin, J. Benet-Buchholz, A. Vidal-Ferran, *RSC Adv.* **2017**, *7*, 32833.

[3] M. H. Haindl, M. B. Schmid, K. Zeitler, R. M. Gschwind, *RSC Adv.* **2012**, *2*, 5941.

[4] M. B. Schmid, K. Zeitler, R. M. Gschwind, *J. Am. Chem. Soc.* **2011**, *133*, 7065.

[5] I. K. Mangion, A. B. Northrup, D. W. C. MacMillan, *Angew. Chem. Int. Ed.* **2004**, *43*, 6722; *Angew. Chem.* **2004**, *116*, 6890.

[6] J. Burés, A. Armstrong, D. G. Blackmond, *J. Am. Chem. Soc.* **2012**, *134*, 6741.

[7] E. Dibello, G. Seoane, D. Gamenara, *Synthetic Commun.* **2015**, *45*, 975.

[8] A. B. Northrup, D. W. C. MacMillan, *J. Am. Chem. Soc.* **2002**, *124*, 6798.
